# Unveiling the Diversity and Modifications of Short Peptides in *Buthus martensii* Scorpion Venom through Liquid Chromatography-High Resolution Mass Spectrometry

**DOI:** 10.3390/toxins16030155

**Published:** 2024-03-16

**Authors:** Ling Zeng, Cangman Zhang, Mingrong Yang, Jianfeng Sun, Jingguang Lu, Huixia Zhang, Jianfeng Qin, Wei Zhang, Zhihong Jiang

**Affiliations:** State Key Laboratory of Quality Research in Chinese Medicine, Macau University of Science and Technology, Macau 999078, China; zengling0619@163.com (L.Z.); cmzhang@must.edu.mo (C.Z.); mryang@must.edu.mo (M.Y.); sjianf@126.com (J.S.); jglu@must.edu.mo (J.L.); zhanghuixia0809@163.com (H.Z.); jf_qin@163.com (J.Q.); wzhang@must.edu.mo (W.Z.)

**Keywords:** scorpion venom, short peptides, de novo sequencing, post-translational modifications, LC-MS^n^

## Abstract

More recently, short peptides in scorpion venom have received much attention because of their potential for drug discovery. Although various biological effects of these short peptides have been found, their studies have been hindered by the lack of structural information especially in modifications. In this study, small peptides from scorpion venom were investigated using high-performance liquid chromatography high-resolution mass spectrometry followed by de novo sequencing. A total of 156 sequences consisting of 2~12 amino acids were temporarily identified from *Buthus martensii* scorpion venom. The identified peptides exhibited various post-translational modifications including N-terminal and C-terminal modifications, in which the N-benzoyl modification was first found in scorpion venom. Moreover, a short peptide Bz-ARF-NH_2_ demonstrated both N-terminal and C-terminal modifications simultaneously, which is extremely rare in natural peptides. In conclusion, this study provides a comprehensive insight into the diversity, modifications, and potential bioactivities of short peptides in scorpion venom.

## 1. Introduction

In traditional Chinese medicine, the scorpion and its venom have been a longstanding remedy for various diseases and syndromes including stroke, rheumatic conditions, convulsions, epilepsy, and more [1]. Scorpion venom comprises a diverse array of elements such as peptides, enzymes, biogenic amines, mucoproteins, and non-protein inclusions such as inorganic salts. Notably, the peptidic fraction emerges as a valuable source of lead compounds for drug development [2]. These peptides are broadly classified into two groups: disulfide bridge-containing peptides (DBPs) and non-disulfide bridge-containing peptides (NDBPs). The main component DBPs, typically ranging from 3000 to 8000 Da, predominantly target membrane-bound ion channels [2]. On the other hand, NDBPs, with most of their molecular weights below 3000 Da, constitute a distinct group of short peptides exhibiting versatile activities such as antimicrobial, anticancer, hemolytic, anti-inflammatory, antihypertensive, and so on [3,4]. Due to their biological activities and expeditious synthesis, these short peptides are highly promising candidates for medicinal development [5].

So far, the investigation of small peptides derived from scorpion venom remains inadequate. Although a comprehensive analysis of short peptides has been carried out on Brazilian *Tityus obscurus* venom, only 27 have been assigned sequences [6]. Similarly, limited research has been conducted on *Buthus martensii* short peptides [7], leaving a significant number of short peptides in scorpion venom with undetermined sequences. The post-translational modification of peptides significantly influences their structural stability and biological activity [8]. This alteration acts as a protective measure against carboxypeptidase degradation and plays a crucial role in the function of peptides [9]. Despite the recognition of over 300 distinct post-translational modifications (PTMs), only four modifications of scorpion venom proteins and peptides have been reported [6,9,10]. The exploration of modified peptides in scorpion venom shows promise and holds significant importance for the development of structurally stable and highly active polypeptide drugs.

Common methods for peptide identification encompass Edman degradation, de novo sequencing, and database searches. Although Edman degradation technology was once a milestone in the development of protein research, liquid chromatography with tandem mass spectrometry (LC-MS/MS) gradually replaced the Edman technique due to its high speed, sensitivity, and applicability to complex peptide mixtures [11]. Database searches serve as powerful tools for protein identification; however, they may not encompass the entirety of available sequences, thereby limiting the discovery of new peptides and often exhibiting constraints when confronted with peptides containing PTMs [12]. The predominant approach employed in recent years for studying scorpion venom has been database searching, yet no novel modifications have been identified in scorpion venom since 2000 [13]. In such cases, de novo sequencing, which is independent of existing data, becomes indispensable. However, de novo sequencing faces challenges in identifying the isomers of isoleucine (I) and leucine (L). Some mass spectrometry-based techniques have been employed to resolve such ambiguities including higher-energy collision-induced dissociation (HCD)-MS^n^, and electron-transfer dissociation (ETD)-HCD-MS^3^ [14,15].

In consideration of the significance and the limited understanding of scorpion venom short peptides, the sequence and PTMs of these derived short peptides should be analyzed. To achieve this objective, 3 kDa ultrafiltration membranes were employed to separate short peptides from long peptides, followed by LC - MS/MS and MS^n^ analysis. HCD MS^n^, ETD-HCD MS^3^ and Mascot search were also implemented to distinguish isomeric I/L residues in peptide sequences. This study could enhance our understanding of scorpion venom and offer new insights for drug research and development.

## 2. Results and Discussion

### 2.1. Characterization of the Sequences of Short Peptides

**Figure 1:**
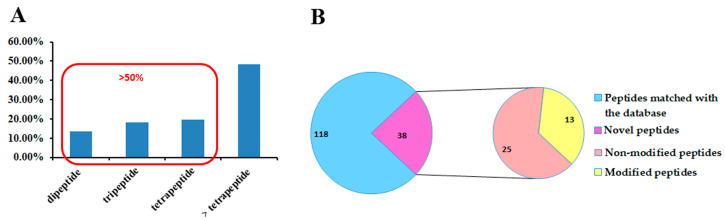
Characterization of de novo sequenced peptides. (**A**) The distribution of identified short peptides in length. (**B**) The classification of the short peptides.

A subset of MS/MS spectra displaying robust signals and a diverse array of sequence-related fragment ions was carefully selected to ensure unequivocal sequence assignment. The mass differences between successive b-ions and y-ions were calculated according to the method for the precise determination of peptide sequences [6]. The peptide sequences were validated by comparing their molecular weights, retention time, and fragmentation information with established standards. The results yielded a comprehensive set of 156 short peptide sequences, as detailed in Supplementary Table S1. Out of the 156 short peptides, 38 were found in venom for the first time, comprising 25 non-modified and 13 modified peptides, as depicted in Figure 1B.

Within the set of 156 short peptides, the briefest consists of merely two amino acid residues, while the most extended sequence encompassed 12 amino acid residues. Figure 1A reveals the distribution of peptide lengths, with dipeptides (13.5%), tripeptides (19.9%), and tetrapeptides (21.1%), collectively making up more than half of the overall count. These types of short peptides demonstrated the potential ability to inhibit ACE activity [16], slow down blood coagulation [17], reflect the expression of proteolytic activity [18], and inhibit the entry of store-operated calcium [19], among other functions. The findings suggest that the size of peptides significantly influences their biological activity. Moreover, in the majority of cases, it is the sequence rather than the size of the peptide that predominantly determines their specific biological activity.

### 2.2. Identification of Leucine/Isoleucine in Short Peptides

Notably, a significant proportion of the total (73%) consists of isoleucine (I) or leucine (L) residues, with 114 out of 156 short peptides containing these amino acids. Some sequences exhibit a high proportion of I or L residues reaching up to 71% of the sequence length. Differences in L and I at the same site result in sequences forming different spatial conformations, which alter the binding affinity and specificity of the sequence [15,20]. The sequences with consecutive occurrences of I or L, such as “I/LI/LP” and “I/LEI/LI/LI/LP” could cause more complex structure modifications and identification processes. In this study, ETD-HCD MS^3^ was employed to identify I/L by utilizing ion fragments of the d- or w-type involving the loss of isopropyl (-C_3_H_7_) or ethyl (-C_2_H_5_) from the side chain of specific a- or z-ions. The presence of L was indicated by the loss of -C_3_H_7_ (43 Da) in a- or z-ions, while a loss of -C_2_H_5_ (29 Da) suggested the presence of I. With this method, three short peptides were fully identified, and part of the multiple I/L sequences in 10 other short peptides were identified. The primary factors contributing to this outcome are the frequent occurrence of I/L residues at the terminus of these exceptionally short peptides (more than 50% shorter than tetrapeptides), as well as their propensity to form multiple connections. These characteristics lead to the misidentification of w_n_ ions, insufficient generation of z-ions [15], and hydrogen extraction phenomenon [21].

The HCD MS^n^ technique serves as a supplementary approach for the identification of short peptides that exclusively contain either I or L residues, exhibiting limited capacity to form more than two charges. The method relies on the breakdown of the 86-Da immonium ion found in I or L, resulting in a distinct fragmentation pattern. Fragmentation of the 86-Da immonium ion derived from I will yield a significant abundance of diagnostic ions weighing 69-Da, while the same ion originating from L will produce minimal (<10%) or no 69-Da ions [15]. Eleven short peptides were identified by this method.

Despite the implementation of these two methods, a significant portion of I/L differentiations within the sequences remained unresolved. To address this challenge, the Mascot search method was employed as a supplementary tool for I/L identification. The sequences were compared with those identified by the Mascot search. This comparison resulted in a total of 32 sequences that were confirmed (Supplementary Table S2). Finally, a total of 83 sequences were successfully identified following I/L verification.

### 2.3. Novel Peptides

The novel peptides in the filtrate of the scorpion venom are presented in Supplementary Table S1, encompassing both modified and non-modified variants. Modified peptides are generally shorter than non-modified peptides, with 9 out of the 13 modified peptides having a length equal to or less than 4 amino acids, whereas non-modified peptides typically range from 4 to 10 amino acids in length. It is worth noting that such abbreviated modified peptides are infrequent in toxins. These short peptides are available in five modified forms, encompassing three N-terminal modifications, one C-terminal modification, and one dual modification of both the N-terminus and the C-terminus.

The C-terminal amidation modification is prevalent in scorpion venom [22]. This section reveals the presence of C-terminal amidation in seven short peptides, constituting over half of the modified peptides. These peptides were also characterized by their short length, typically consisting of 3–8 amino acids. Previous studies on scorpion venom peptides have reported complete sequences of amidated peptides obtained from cDNA libraries, which were longer than 14 residues [3]. Additionally, fragments derived from larger proteins or peptides, such as the 48–58 fragment of the antigen 5-like protein (NIALGQDQSGR-NH_2_) [5], were found to be more than 10 amino acids in length [6]. Amidation is considered as part of the toxin maturation process, playing an important role in their biological activities and significantly enhancing the stability of the toxin [6].

#### 2.3.1. N-Terminal Modified Peptides

Among the peptides with N-terminal modifications, three peptides were benzoylated, one was carboxybenzoylated, and two underwent pyro-Glu modification.

**Figure 2:**
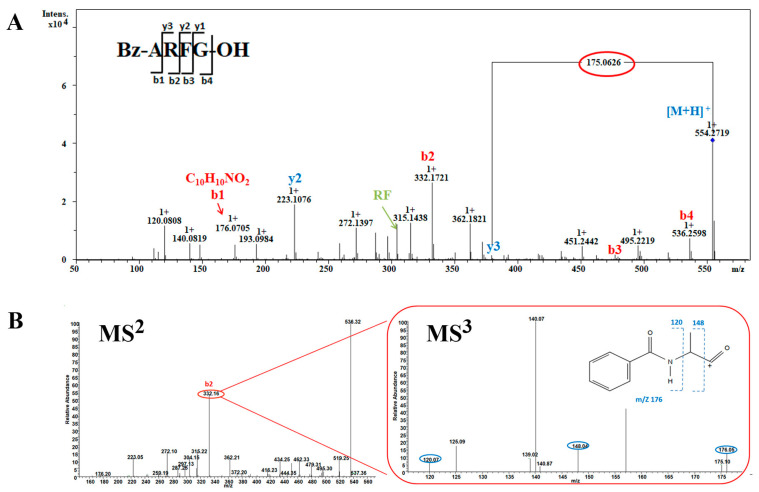
MS analysis of peptide Bz-ARFG. (**A**) Bruker QTOF-CID MS^2^ spectrum of the single charged precursor ion at *m*/*z* 554.2719 Da; (**B**) LTQ-Orbitrap-CID MS ^2^ spectrum of the precursor ion at *m*/*z* 554.2719 Da and MS^3^ spectrum of the b2 ion at *m*/*z* 332.16 Da (in red circles). The blue circle demonstrates the associated fragment ions of the Bz-A residue.

**Figure 3:**
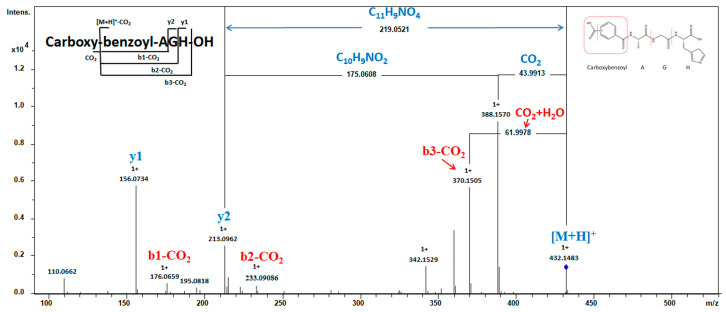
MS analysis of peptide Carboxybenzoyl-AGH. MS^2^ spectra were obtained under CID conditions by selecting the precursor ion of *m/z* 432.1483 as [M + H]^+^ for fragmentation, showing the fragment ions used to assign the peptide sequence.

Three peptides Bz-ARF, Bz-ARFG, and Bz-AFGH, all containing N-terminal benzoyl modification with a mass of 176.0705 Da, were identified. To elucidate the process, the peptide Bz-ARFG with *m*/*z* 554.2719 Da was selected for demonstration (Figure 2). The series of ‘b’ and ‘y’ ions were meticulously analyzed, yielding the observation of a majority of fragment ions crucial for sequence determination. This enabled the estimation of three C-terminal residues, while N-terminal residues with a mass of *m*/*z* 176.0705 Da remained uncharacterized. Based on its precise mass, the molecular formula of *m*/*z* 176.0705 Da was initially determined to be [C_10_H_10_NO_2_]^+^ (Figure 2A) corresponding to the two most plausible candidates: phenylacetylglycine or benzoylalanine (Bz-A). To elucidate the structure of ion *m*/*z* 176.0705, the highly abundant b2-ion (*m*/*z* 332.16 Da) was selected for analysis using Orbitrap MS^n^ spectrometry (Figure 2B). In the MS^3^ step, the *m*/*z* 332.16 Da ion effectively generated characteristic fragments with *m*/*z* values of 176.05 Da (b1), 148.04 Da (a1), and 120.07 Da (a1-C_2_H_4_), respectively (Figure 3B). According to the loss fragments, the modified peptide was determined as Bz-ARFG.

Benzoylation of amino acids is frequently observed in histones [23,24,25]. Nevertheless, the significance of benzoyl-modified peptides in animal venom has not been previously documented. The N-terminal modification of peptides is recognized for its ability to enhance stability and to either maintain or even improve peptide activity [26].

The current investigation elucidates the identification of an N-terminal modified peptide, specifically Carboxybenzoyl-AGH, with a precursor ion *m*/*z* of 432.1483 Da (Figure 3). The sequence can be determined by analyzing the necessary fragment ions (b- and y-related ions) except for the N-terminal residue, which could not be preliminarily identified. To elucidate the structure of the N-terminal, a comprehensive examination was conducted on the spectrum. The y2 ion exhibited a mass loss of 219.0521 Da (C_11_H_9_NO_4_) from [M + H]^+^, which can be attributed to the removal of two groups. One was the carboxylic acid group (COOH) cleaved from the short peptide, resulting in a reduction of 43.9913 Da in [M + H]^+^ and yielding an ion with *m*/*z* 388.1570. The second was Bz-A, detached from the ion at *m*/*z* 388.1570, leading to a decrease of 175.0608 Da (C_10_H_9_NO_2_). This hypothesis is further confirmed by b-series ions. A neutral loss of 61.9978 Da occurred from the mass of *m*/*z* 432.1483 ([M + H]^+^) to *m*/*z* 370.1505, corresponding to CO_2_ and H_2_O release, suggesting that COOH in the peptide possesses lower bond energy and dissociates first within the sequence. The Bz-A residue corresponds to the b-series ion with an *m*/*z* value of 176.0659. Therefore, the N-terminal structure was determined as a carboxyl benzoylalanine residue, with the precise location of the carboxyl group within the phenyl ring of the benzoyl group yet to be ascertained. Previous research showed that compounds derived from carboxybenzoyl of the amino acid series exhibit promising therapeutic potential by effectively inhibiting the growth of the MDA-MB-231 cell line [27] and the carboxybenzoyl-AGH peptide may have potential as an anticancer agent.

The amino acid sequences of two pyro-Glu (pE) peptides, pEDWI/LP and pEDWI/LPS, were determined. Both peptides exhibit N-terminal modification with pE. The peptide pEDWI/LP was found to have a significant overlap with pEDWI/LPS, suggesting that the former might be generated through the hydrolysis of the latter. Revealing the structures of both peptides was achieved through an analysis of characterized fragments using MS and MS^2^ data (Supplementary Figures S5 and S6). The b- and y- ions of both peptides were observed, allowing for the determination of amino acids in the sequence, except for the N-terminal residues. It should be noted that the N-terminal group has a mass of 226.0605 Da, which is 18.0100 Da less than the Glu-Asp (ED) residue and is presumed to be pED [28].

Pyroglutamate peptides have been identified in some animal venoms [8,13,28,29,30,31,32,33]. The prevalence of pyroglutamate-modified peptides is higher in conotoxin [8] and snake venom [34], with fewer occurrences in scorpion venoms [13,33]. The generation of pyroglutamic acid at the N-terminus can greatly enhance the blocking effect of toxins for their target channels or stabilize against N-terminal degradation [9]. The occurrence of N-terminal pyroglutamate peptides from scorpion venom has been predominantly reported in K^+^ channel-specific toxins, which are composed of 30 to 40 amino acids and possess three disulfide bonds [9,13,33,35,36,37,38,39]. These toxins are derived from DBPs. Interestingly, our research focuses on modified peptides that are NDBPs found in scorpion venoms with a significantly reduced length of only 5–6 amino acids. The origin of these short peptides, whether they are derived from hydrolyzed fragments of longer DBPs or naturally present in the venom with unique functionality akin to BPPs in snake venom, remains uncertain. Further investigations are warranted to elucidate their precise role within venomous systems.

#### 2.3.2. N- and C-Terminal Modified Peptides

**Figure 4:**
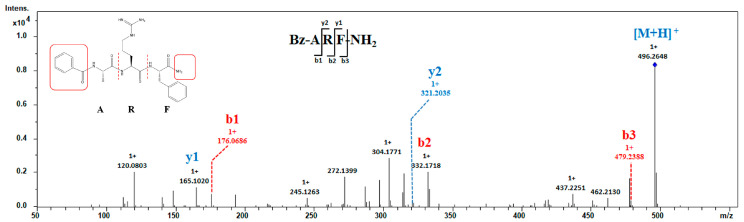
MS^2^ analysis of peptide Bz-ARF-NH_2_. MS^2^ spectra was obtained under CID conditions, selecting the precursor-ion of *m*/*z* 496.2648 as [M + H]^+^ for fragmentation. The resulting MS^2^ spectra displayed the b- and y- series of fragment ions, facilitating the assignment of the peptide sequence. Both the N-terminal and C-terminal of this peptide are modified.

A tripeptide exhibiting both N- and C-terminal modifications was identified. The MS^2^ spectrum of Bz-ARF-NH_2_ (precursor ion: *m*/*z* 432.1483 Da) is depicted in Figure 4. The mass-to-charge ratio of b1 is 176.0686, indicating a correlation with the residue of benzoyl-A, suggesting that the N-terminal of this peptide is modified by benzoyl. The mass of b3 is 17.026 Da lower than [M + H]^+^, implying a modification at the C-terminal with amide. The presence of an ion of 165.1020 Da corresponding to y1 further substantiated this observation, suggesting the amidation of the C-terminal amino acid phenylalanine. The presence of other crucial fragment ions (b- and y-ions) necessary for sequence determination was also observed, thereby enabling the identification of the peptide sequence as Bz-ARF-NH_2_. Simultaneous modification of the N- and C-termini in short peptides is exceptionally rare in natural peptides. Only a limited number of toxins have been identified to possess disulfide bonds spanning approximately 17 amino acids, with modifications at both the C- and N-terminals involving pyroglutamic acid at the C-terminus and amide at the N-terminus [40]. Kuzmin *et al*. synthesized tachyplesin I with N-terminal acetylation and C-terminal amidation modifications, subsequently comparing its activity to that of unmodified tachyplesin I. Their findings demonstrated that tachyplesin I, with concurrent modifications at both the C-terminal and N-terminal regions, exhibited enhanced cytotoxicity against cancer cells and displayed improved pharmacokinetic properties. Consequently, it represents a more promising prototype for the development of anticancer drugs [41]. These findings provide valuable insights for further investigation of the activity of the short peptide Bz-ARF-NH_2_.

An intriguing observation was made regarding the peptides Bz-ARFG and Bz-ARF, as they exhibited significant overlap with the modified peptide Bz-ARF-NH_2_, suggesting a potential rational transformation pathway for these peptides. The transformation of Bz-ARFG to Bz-ARF could be attributed to the action of carboxypeptidase, which excises the “G” at the C-terminus, resulting in Bz-ARF. On the other hand, the conversion of Bz-ARFG to Bz-ARF-NH_2_ involved a two-step enzymatic process. Initially, a peptidylglycine α-hydroxylating monooxygenase (PHM) catalyzes the hydroxylation of the glycine residue. Subsequently, a peptidyl-α-hydroxyglycine α-amidating lyase (PAL) cleaves the hydroxyglycine residue, producing the amidated product [42].

#### 2.3.3. Non-Modified Peptides

**Figure 5:**
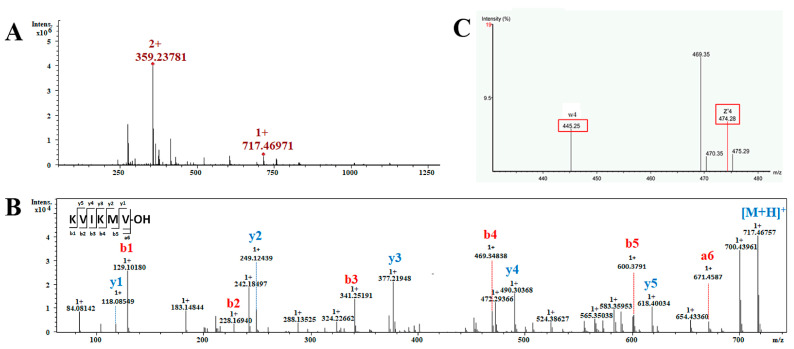
MS analysis of the peptide KVIKMV. (**A**) ESI-MS spectrum obtained in positive mode for the peptide, showing the ion of *m*/*z* 358.23781 as [M + 2H]^2+^ and *m*/*z* 717.4697 as [M + H]^+^; (**B**) MS^2^ spectra obtained under CID conditions obtained by selecting the precursor-ion of *m*/*z* 358.23781 as [M + 2H]^2+^ for fragmentation, showing the b and y series of fragment ions used to assign the peptide sequence; (**C**) MS^3^ spectra obtained under ETD conditions, showing the w-ion used to diagnostically eliminate the ambiguity between the isobaric residues I/L. The w4 ion was detected and lost a mass of 29.03 Da from z4, thus the I/L residue can be identified as Ile.

This section encompasses the analysis of a total of 25 short non-modification peptides, with lengths ranging from 4 to 10 amino acids. The peptide KVIKMV was chosen as an exemplar to elucidate the process of sequence determination (Figure 5). The MS^1^ spectrum of this peptide in Figure 5A displays two prominent peaks: one at *m*/*z* 358.23781 corresponding to the [M + 2H]^2+^ ion and another at *m*/*z* 717.4697 indicating the presence of the [M + H]^+^ ion. The intense precursor ion with an *m*/*z* of 358.23781 as [M + 2H]^2+^ was employed for *de novo* sequencing. The analysis of the MS^2^ data revealed a comprehensive array of b-type and y-type fragment ions, facilitating the confident assignment of the peptide’s sequence as KVI/LKMV (Figure 5B). Based on this sequence and the aforementioned molecular mass, it is suggested that the peptide’s C-terminal residue exists in its acidic form. To distinguish between the isobaric residues I and L within the sequence, the ion *m*/*z* 358.23781 was analyzed using an Obitrap mass spectrometer with ETD to obtain d or w ions for differentiation. The w4 ion detected in the MS^3^ spectrum exhibited a mass loss of 29.03 Da (-C_2_H_5_) from z4 (Figure 5C). Then, the isobaric residue unambiguously confirmed the residue as I. Consequently, the complete sequence of the peptide was determined as KVIKMV-OH.

Among the 25 short peptides (Supplementary Table S1), the peptide KVI/LKM (No. 15) undergoes oxidation at the M amino acid residue (No. 16). Seven of these short peptides with the No. of 38, 37, 35, 31, 24, 23, and 19 exhibit sequence overlap, suggesting potential cleavage of the peptide No. 38 and resulting in the generation of six shorter peptides. It is noteworthy that the proteolytic activity of scorpion venom has been demonstrated to be significantly influenced by pH values below a certain threshold while remaining undetectable at pH 4 [10]. In contrast, our venom separation was conducted under acidic conditions (pH~2) using 0.1% formic acid. Furthermore, the majority of peptides in the filtrate obtained through the 3K Da filter were less than 3K Da and thus devoid of enzymatic activity. Consequently, it is improbable that the observed protein hydrolysis fragments in our study are attributable to sample treatment.

This phenomenon of overlapping peptides resulting from cleavage events is not uncommon in scorpion venom research [6,10]. The cleavage can take place in the C-terminal, N-terminal, or both simultaneously. It is hypothesized that proteolysis is a kind of post-translational processing of the toxins [43]. Therefore, proteolysis appears to be a common PTM in scorpion venom, potentially diversifying the molecular repertoire and leading to various distinct molecular targets and modes of action [10]. The post-splitting mechanism observed in scorpions reflects an evolutionary strategy that has led to the development of an impressively diverse venom repertoire [44]. This diversity not only contributes to the adaptability of scorpions in their ecological niches but also presents opportunities for further investigation into the biological activities and therapeutic potentials of these peptides.

### 2.4. Activity Analysis In Silico

The antihypertensive Peptide Database (AHTPDB) was searched to determine ten dipeptides with antihypertensive properties (Table 1). Most of these peptides exhibited high scores on the Peptide Ranker server, with over 50% achieving a score above 0.9. Antihypertensive peptides have been widely identified in plant and animal sources such as soybean and milk, but rarely found in animal toxins [28,29,30,31]. The first antihypertensive peptide isolated from *Tityus serrulatus* scorpion venom contained 13 residues [45], then another antihypertensive peptide found in *Tityus stigmurus* venom consisted of 25 amino acid residues [46]. Remarkably, all ten antihypertensive peptides discovered in this research contain only two residues. The existence of such a considerable number of antihypertensive dipeptides in scorpion venom had not been reported previously.

The subsequent search conducted in the BIOPEP database revealed that the ten peptides also exhibited other potential functionalities such as stimulation and inhibition of dipeptidyl peptidase IV and dipeptidyl peptidase III in addition to their ACE-inhibitory activity. This comprehensive analysis enhances our understanding of the potential multifunctionality of scorpion venom short peptides, expanding our knowledge of their biological roles.

## 3. Conclusions

The study utilized a series of sophisticated mass spectrometry techniques and detailed sequence allocation methods to successfully identify 156 peptides, varying in length from 2 to 12 amino acids. Ten dipeptides exhibiting antihypertensive properties were discerned. Furthermore, 38 novel short peptides were uncovered in scorpion venom. Particularly noteworthy is the identification of five kinds of modified short peptides, three of which had not been previously reported in scorpion venom. In summary, this investigation presents a comprehensive characterization of short peptides in scorpion venom, elucidating their diversity, modifications, and potential biological activities. These findings not only advance our comprehension of scorpion venom short peptides but also provide fresh insights for drug development.

## 4. Materials and Methods

### 4.1. Source of Scorpion and Venom Extraction

The scorpions of the *Buthus martensii* Karsch species were obtained from Licheng Scorpion Farm, located in Dengfeng City, Henan Province. To collect crude venom, electrical stimulation of the scorpion’s telson was employed. The venom was collected in a 1.5 mL Eppendorf tube placed on ice. Subsequently, the collected venom was lyophilized and stored at −80 °C until it was ready for use.

### 4.2. Sample Preparation

For sample preparation, 1 mg of the crude venom was initially dissolved in 200 µL of ultrapure water containing 0.1% formic acid (FA). Following this, the solution was centrifuged at 14,000 rpm for 5 min. The resulting supernatant was carefully collected in a 1.5 mL Eppendorf tube and subsequently subjected to filtration using a 3K Centrifugal Filter (Amicon^®^ Ultra; 0.5 mL). The filtrate was collected and then further purified using an HLB solid-phase extraction cartridge. After purification, the sample was lyophilized and subsequently dissolved in 100 µL of mobile phase A (composed of ultrapure water with 0.1% FA) in preparation for LC-MS/MS analysis.

### 4.3. LC–MS^n^ Analysis

The sequencing of peptides was conducted using a Bruker Q-TOF spectrometer (Bruker Daltoniks, Bremen, Germany), which was coupled to an Agilent 1290 Infinity Binary LC system (UHPLC, Santa Clara, CA, USA). A column of ACQUITY HSS C-18 (Waters), measuring 150 mm × 2.1 mm with a particle size of 1.8 μm, was employed for chromatographic separation. The system operated at a flow rate of 300 μL/min. Solvent A consisted of water containing 0.1% formic FA, while solvent B comprised acetonitrile with 0.1% FA. The elution process followed this gradient: 0–5 min, 0–8% solvent B; 5–15 min, 8–20% solvent B; 15–30 min, 20–50% solvent B; 30–35 min, 50–95% solvent B; and 35–40 min, 95% solvent B. The column temperature was maintained at 30 °C. Mass spectra were acquired in positive mode, covering a mass range of 200–2500 *m*/*z*. The data acquisition parameters included an absolute threshold of 800, an end plate offset of 500 V, a capillary voltage set at 4000 V, a nebulizer pressure of 2.5 bar, a dry gas flow rate of 8.0 L/min, and a dry temperature of 180 °C. For MS^2^ fragmentation, the top three ions from each MS spectrum were selected as precursors.

To identify isomers Leucine (L) and Isoleucine (I) within the sequence, an additional separation using the Orbitrap Fusion Lumos mass spectrometer (Thermo Fisher Scientific, San Jose, CA, USA) was conducted. Samples were loaded onto a nanoViper C18 pre-column (3 μm, 100 A) with a volume of 2 μL and subsequently desalted with a 20 μL rinse. The separation system utilized was an Easy nLC 1200 nanoliter liquid phase system (ThermoFisher, San Jose, CA, USA), coupled with the ThermoFisher Orbitrap Fusion Lumos mass spectrometer. The desalted samples were retained on the pre-column and then separated using an analytical column, the C18 reverse-phase chromatography column (Acclaim PepMap RSLC, 75 μm × 25 cm, C18-2 μm, 100 A). The mobile phase B gradient (80% acetonitrile, 0.1% formic acid) increased from 5% to 40% over 60 min. The spray voltage was set at 1.9 kV, and the ion transport tube was heated to 275 °C. Mass spectrometry was performed in Data Dependent Analysis (DDA) mode, with a primary mass spectrometry scanning resolution of 70,000, a scanning range of 350–2000 *m*/*z*, and a maximum injection time of 100 ms. In each DDA cycle, up to 20 secondary spectra were collected, with charge states ranging from 2+ to 5+. The maximum injection time for secondary mass spectrometry ions was set to 50 ms. For all precursor ions, the impact chamber energy (high-energy collision-induced dissociation, HCD, or electron-transfer/higher-energy collision dissociation, EThCD) was fixed at 28 eV, and a dynamic exclusion period of 25 s was applied.

The data obtained from the Bruker Q-TOF spectrometer were processed using the Bruker DataAnalysis software (ver. 4.1). The data generated by the ThermoFisher Orbitrap Fusion Lumos mass spectrometer were analyzed using the Thermo Xcalibur Qual Browser tool 4.0.27.13 programs (Thermo Scientific, San Jose, CA, USA).

### 4.4. Mascot Search

The MS/MS spectra were converted into the Mascot generic format (.mgf) using the Bruker DataAnalysis software. Subsequently, Mascot Daemon v. 2.4.1 (Matrix Science) was employed for conducting database searches against the database (taxid: 34649), which had been downloaded from the NCBI website. For protein and peptide identification, the protein sequences from the scorpion venom deposited in the NCBI database were utilized. The obtained MS/MS spectra were subjected to analysis through an MS/MS Ion Search against this database, utilizing the following parameters: no enzyme specificity selected, allowing for zero missed cleavages, modification oxidation (M) and Amidated (C-term), a peptide mass tolerance of 50 ppm, and a fragment mass tolerance of 0.02 Da. To enhance the accuracy of the search results, an automatic decoy search was performed by selecting the Decoy checkbox on the search form. Identifications of proteins or peptides that matched with at least one red and bold peptide were considered correct identifications, and hits with a *p*-value less than 0.05 were deemed significant.

### 4.5. Activity Prediction In Silico

To identify short peptides exhibiting antihypertensive properties, a comprehensive search was conducted on the Antihypertensive Peptide Database (AHTPDB) available at https://webs.iiitd.edu.in/raghava/ahtpdb/pep.php (accessed on 2 January 2024). The potential activity of peptides was predicted using the BIOPEP database https://biochemia.uwm.edu.pl/biopep-uwm/ (accessed on 2 January 2024) [47]. The bioactivities of the peptides were predicted using the Peptide Ranker software, available at http://distilldeep.ucd.ie/PeptideRanker/ (accessed on 3 January 2024). The PeptideRanker model was trained using a threshold of 0.5, where peptides predicted above this threshold are considered to possess bioactivity. Additionally, peptides with a Score > 0.9 are classified as highly bioactive. Sequences containing I/L (where I or L have not been definitively determined) are subjected to prediction for all possible sequences expressed within a given range.

## Figures and Tables

**Table 1:** Potential bioactivities of peptides from the filtrate of the scorpion venom.

No.	Sequence	Peptide Ranker Score	Bioactivities Analysis In Silico	
			AHTPDB Database	BIOPEP Database
1	I/LV	0.06~0.10	ACE inhibitor	stimulating
2	I/LI/L	0.22~0.62	ACE inhibitor	stimulating, dipeptidyl peptidase IV inhibitor
3	FI/L	0.95~0.99	ACE inhibitor	no record
4	IF	0.95	ACE inhibitor	ACE inhibitor
5	DF	0.94	ACE inhibitor	ACE inhibitor
6	FQ	0.86	ACE inhibitor	ACE inhibitor, dipeptidyl peptidase IV inhibitor
7	VW	0.80	ACE inhibitor	ACE inhibitor, antioxidative, alpha-glucosidase inhibitor, dipeptidyl peptidase IV inhibitor
8	FF	1.00	ACE inhibitor	ACE inhibitor, dipeptidyl peptidase IV inhibitor
9	I/LW	0.94~0.99	ACE inhibitor	ACE inhibitor, dipeptidyl peptidase IV inhibitor
10	FR	0.99	ACE inhibitor	ACE inhibitor, dipeptidyl peptidase IV inhibitor, dipeptidyl peptidase IV inhibitor

## Data Availability

The venom sequences of scorpion *B. martensii* Karsch were retrieved from the NCBI database (https://www.ncbi.nlm.nih.gov/protein/?term=txid34649[Organism:noexp], accessed on 10 November 2023), with Taxid accession: 34649.

## Supplementary Materials

The following supporting information can be downloaded at: https://www.mdpi.com/article/10.3390/toxins16030155/s1, Figure S1–S156: Mass spectra and *de novo* sequencing analysis of the peptides; Table S1: List of *de novo* sequencing peptides in the filtrate of scorpion Buthus martensii Karsch venom; Table S2: *De novo* sequence peptides matching with the sequences of MASCOT research.
