# Supplementary material for: Unveiling the Diversity and Modifications of Short Peptides in Buthus martensii Scorpion Venom through Liquid Chromatography-High Resolution Mass Spectrometry
Source: Toxins (Basel). 2024 Mar 16;16(3):155. doi: 10.3390/toxins16030155 (PMC10975176; doi:10.3390/toxins16030155)
Supplement: Supplementary file 1 [file toxins-16-00155-s001.zip › toxins-2893015-supplementary.pdf]

Unveiling the Diversity and Modifications of Short Peptides in *Buthus martensii* Scorpion Venom through Liquid Chromatography-High Resolution Mass Spectrometry

Ling Zeng, Cangman Zhang, Mingrong Yang, Jianfeng Sun, Jingguang Lu, Huixia Zhang, Jianfeng Qin, Wei Zhang and Zhihong Jiang

Supplementary Figures and Tables

Supplementary Table 1……………………………………………………………………2~5
Supplementary Table 2………………………………………………………………………6
Supplementary Figures 1~156……………………………… …………………………..7~54

Supplementary Table 1. List of de novo sequencing peptides in the filtrate of scorpion *Buthus martensii* Karsch venom.

	No.	Ion for MS/MS (m/z) (charge)	RT	MS observed	Delta (Da)	sequence
Novel peptides	N-terminal modification					
	1	432.1483(+1)	9.32	431.1405	0.0036	Carboxybenzoyl-AGH
	2	497.2501(+1)	21.70	496.2423	0.0011	Bz-ARF
	3	554.2719(+1)	19.92	553.2641	0.0008	Bz-ARFG
	4	535.2359(+1)	20.63	534.2281	0.0059	Bz-A FGH
	5	641.2937(+1)	22.71	640.2859	0.0007	pEDWI/LP
	6	728.3301(+1)	21.05	727.3223	-0.0051	pEDWI/LPS
	7	335.2074(+1)	14.13	334.1996	0.0009	FGI- NH_2◆
	C-terminal modification					
	8	431.2364(+1)	10.92	430.2286	0.0043	WVGA- NH_2
	9	438.2336(+1)	6.68	437.2258	0.0016	YVTG- NH_2
	10	562.2607(+1)	8.20	561.2529	0.0018	DKWD- NH_2
	11	569.3072(+1)	19.91	568.2994	0.0015	SFGFI/L - NH_2
	12	321.1942 (+3)	2.08	960.5592	0.0215	I/LFQAI/LKNE-NH_2
	N-terminal and C-terminal modification					
	13	496.2648(+1)	20.22	495.2570	0.0019	Bz-ARF-NH_2
	Non-modification					
	14	507.2428(+1)	12.81	506.2350	0.0076	FFGH
	15	309.7041 (+2)	7.84	617.3926	0.0002	KVI/LKM
	16	317.6992(+2)	1.73	633.3828	0.0045	KVI/LKM(oxidation)
	17	320.1854(+2)	19.74	638.3552	0.0022	FRFGI/L
	18	321.1544(+2)	16.86	640.2932	0.0069	FRFGD
	19	321.73 60(+2)	13.14	641.4564	0.0014	I/LR I/LI/LK
	20	337.1757 (+2)	17.75	672.3358	0.0015	FFRFG
	21	337.1757 (+1)	19.63	672.3358	0.0015	FRFGF
	22	359.2374(+2)	11.73	716.4592	0.0016	KVIKMV #
	23	364.2619(+2)	10.40	726.5082	0.0023	I/LVKVI/LR
	24	371.2672 (+2)	13.18	740.5188	0.0074	VI/LRI/LI/LK
	25	389.704 (+2)	9.47	777.3924	0.0026	TPSYI/LPT
	26	785.4411(+1)	18.36	784.4333	0.0008	I/LI/LPDAI/LSG
	27	413.2751 (+2)	18.04	824.5346	0.0015	I/LLEI/LIPK
	28	413.2713 (+2)	21.35	824.5270	0.0091	I/LEI/LKI/LI/LP
	29	430.265 (+2)	9.78	858.5144	0.0020	KIVDGI/LKS
	30	431.2126 (+2)	18.37	860.4096	0.0074	WLDKWN
	31	290.5471 (+3)	9.64	868.6179	0.0027	VI/LRI/LI/LKK
	32	435.7661 (+2)	17.65	869.5166	0.0044	I/LAEKI/LLSP
	33	438.7600 (+2)	15.07	875.5044	0.0062	FATKLI/LPS
	34	306.1918 (+3)	9.90	915.5520	0.0006	I/LKKVWKD
	35	477.3446 (+2)	17.82	952.6736	-0.0064	I/LVKVI/LRI/LI/L

36	477.3304(+2)	18.74	952.6452	0.0029	KI/LNKI/LI/LPK
37	361.2627 (+3)	10.82	1080.7647	0.0084	I/LVKVI/LRI/LI/LK
38	303.2243 (+4)	12.55	1208.8660	0.0016	I/LVKVIRI/LIKK
Peptides matching with the database					
Modified peptides					
39	278.186(+1)	10.36	277.1782	0.0003	IF-NH_2◆
40	278.1883(+1)	9.67	277.1805	0.0020	FI/L-NH_2
41	293.1612(+1)	5.87	292.1534	0.0004	FGA-NH_2
42	293.1951(+1)	2.05	292.1873	0.0021	FK-NH_2
43	294.1452(+1)	6.05	293.1374	0.0004	EF-NH_2
44	319.1429(+1)	6.78	318.1351	0.0028	WD-NH_2
45	391.2695(+1)	18.07	390.2617	0.0009	I/LI/LF-NH_2
46	425.251(+1)	19.55	424.2432	0.0037	FI/LF-NH_2
47	429.281(+1)	9.44	428.2732	0.0009	IGAVA-NH_2*
48	468.272(+1)	14.22	467.2642	0.0002	FRF-NH_2
49	576.3456(+1)	13.74	575.3378	0.0048	FI/LGAGI/L-NH_2
50	576.3528(+1)	15.89	575.3450	0.0024	FIGAVA-NH_2*
51	291.1788(+1)	16.36	580.3420	0.0055	I/LFRF-NH_2
52	339.2201 (+1)	12.91	676.4246	0.0015	LISAFK-NH_2*
53	699.4399(+1)	16.93	698.4321	0.0000	LIPSAIS-NH_2*
Non-modification					
54	231.1684(+1)	6.33	230.1606	0.0019	I/LV
55	245.184(+1)	12.04	244.1762	0.0020	I/LI/L
56	253.116(+1)	8.98	252.1082	0.0023	FS
57	279.173(+1)	16.14	278.1652	0.0027	FI/L
58	279.1732(+1)	16.33	278.1654	0.0029	IF◆
59	281.1107(+1)	3.48	280.1029	0.0025	FD
60	281.1109(+1)	4.46	280.1031	0.0023	DF
61	294.1421(+1)	3.27	293.1343	0.0027	FQ
62	295.1647(+1)	11.75	294.1569	0.0005	YI/L
63	304.1656(+1)	13.66	303.1578	0.0000	VW
64	313.1593(+1)	16.84	312.1515	0.0046	FF
65	318.1778(+1)	15.69	317.1700	0.0034	WI/L
66	318.1778(+1)	15.92	317.1700	0.0034	I/LW
67	318.1778(+1)	17.15	317.1700	0.0034	I/LW
68	320.1279(+1)	7.86	319.1201	0.0038	WD
69	322.1905(+1)	3.66	321.1827	0.0031	FR
70	332.2187(+1)	8.68	331.2109	0.0007	LLS◆
71	336.1888(+1)	12.58	335.1810	0.0030	FI/LG
72	342.2393(+1)	14.66	341.2315	0.0006	I/LI/LP
73	360.1635(+1)	12.23	359.1557	0.0031	FGH
74	360.213(+1)	11.94	359.2052	0.0001	I/LI/LD
75	366.1989(+1)	13.76	365.1911	0.0034	I/LFS
76	366.1989(+1)	18.27	365.1911	0.0034	I/LSF
77	366.2000(+1)	23.33	365.1944	0.0001	FSI/L

78	366.2017(+1)	9.02	365.1939	0.0006	YI/LA
79	377.1816(+1)	11.24	376.1738	0.0003	FNP
80	378.1667(+1)	11.02	377.1589	0.0007	FDP
81	392.2173(+1)	13.43	391.2095	0.0007	YI/LP
82	392.2527(+1)	21.76	391.2449	0.0017	I/LFI/L
83	400.1903(+1)	18.78	399.1825	0.0036	FSF
84	403.259(+1)	11.55	402.2512	0.0039	I/LI/LSA
85	407.2331(+1)	14.14	406.2253	0.0042	FIGA◆
86	407.2641(+1)	16.13	406.2563	0.0012	FI/LK
87	415.2385(+1)	17.45	414.2307	0.0045	WI/LP
88	420.2245(+1)	8.16	419.2167	0.0004	WKS
89	426.2440(+1)	22.46	425.2362	0.0053	FLF◆
90	429.2700(+1)	12.48	428.2628	0.0002	I/LI/LPS
91	431.2516(+1)	13.78	430.2438	0.0016	I/LI/LDA
92	433.2087(+1)	16.46	432.2009	0.0005	WI/LD
93	435.2712(+1)	10.24	434.2634	0.0002	LFR◆
94	441.2485(+1)	19.56	440.2407	0.0011	FKF
95	445.3008(+1)	20.64	444.2930	0.0013	I/LI/LSI/L
96	227.6713(+2)	19.54	453.3270	0.0016	I/LFKF
97	469.2563(+1)	16.65	468.2485	0.0005	FRF
98	469.2611(+1)	15.66	468.2533	0.0053	FFR
99	479.2845(+1)	21.92	478.2767	0.0019	I/LFSI/L
100	479.2850(+1)	20.66	478.2772	0.0014	I/LI/LSF
101	479.2800(+1)	19.3	478.2767	0.0019	I/LI/LFS
102	483.2086(+1)	8.54	482.2008	0.0000	FTTD
103	493.2657(+1)	11.23	492.2579	0.0000	YI/LPT
104	494.2300(+1)	5.15	493.2281	0.0001	GRFD
105	500.3072(+1)	14.05	499.2994	0.0013	LIPSA*
106	502.2668(+1)	15.53	501.2590	0.0008	WLPS◆
107	506.2949 (+1)	17.76	505.2871	0.0024	FIGAV*
108	513.2692(+1)	23.87	512.2614	0.0016	FI/LSF
109	513.2731(+1)	21.03	512.2653	0.0023	FLFS◆
110	513.275(+1)	22.36	512.2672	0.0042	FSI/LF
111	513.2755(+1)	21.34	512.2677	0.0047	I/LFSF
112	260.1513(+2)	8.17	518.2870	0.0022	VWSK
113	520.3459(+1)	19.14	519.3381	0.0034	I/LFI/LK
114	522.3276(+1)	10.75	521.3198	0.0010	I/LVYK
115	526.276(+1)	15.23	525.2682	0.0012	FRFG
116	529.2715(+1)	17.17	528.2637	0.0058	YI/LFS
117	541.2648(+1)	21.44	540.2570	0.0009	FI/LFD
118	541.2648(+1)	24.67	540.2570	0.0009	FI/LDF
119	545.3302(+1)	10.14	544.3224	0.0009	IIQTA*
120	550.3227(+1)	20.08	549.3149	0.0009	LISAF*
121	554.2716(+1)	20.68	553.2638	0.0107	FPSFG

122	554.3302(+1)	19.34	553.3224	0.0035	LFFK◆
123	279.1857(+2)	11.32	556.3558	0.0016	KIIPS*
124	296.1894(+2)	6.75	590.3632	0.0010	I/LRFR
125	307.1578 (+2)	13.26	612.3000	0.0009	FRFGS*
126	307.2200(+2)	16.84	612.4286	0.0026	VI/LRI/LI/L
127	309.6851(+2)	12.06	617.3546	0.0013	IMKNI*
128	634.3594(+1)	16.23	633.3516	0.0035	FIGAVAG*
129	321.7094(+2)	12.65	641.4032	0.0080	ATKIIP*
130	324.1973(+2)	6.97	646.3790	0.0002	KVWKS*
131	326.1894 (+2)	12.56	650.3632	0.0003	LLKDY*
132	337.2224(+2)	12.74	672.4292	0.0021	LKKVW*
133	697.4512(+1)	23.17	696.4434	0.0018	LEILIP*
134	365.2312(+2)	10.94	728.4468	0.0036	ATKIIPS*
135	378.2532(+2)	13.48	754.4908	0.0035	LATKIIP*
136	379.2250(+1)	14.34	756.4344	0.0027	ATKI/LI/LPD
137	757.4463(+1)	17.23	756.4385	0.0009	LIPSAISG*
138	380.6880(+2)	18.97	759.3620	0.0039	FGVFGSF
139	380.6923(+2)	20.23	759.3690	0.0004	FRFGSF*
140	391.2316(+2)	10.84	780.4476	0.0007	IIQTAVH*
141	785.4413(+1)	18.68	784.4335	0.0010	I/LI/LPSAI/LDG
142	416.257(+2)	11.75	830.4988	0.0020	VWKSKLA*
143	421.7700(+2)	15.67	841.5404	0.0019	LATKIIPS*
144	844.4780(+1)	19.98	843.4702	0.0005	SLIPSALSG*
145	292.5047(+3)	8.08	874.4907	0.0022	IMKNIKE*
146	296.1683(+3)	6.84	885.4815	0.0009	GLREKHF*#
147	444.7862 (+2)	5.36	887.5568	0.0014	LKKVWKS*#
148	303.8552 (+3)	7.87	908.5422	0.0007	IIQTAVKH*
149	478.3125 (+2)	18.96	954.6094	0.0009	LATKIIPSL*#
150	991.5486(+1)	22.84	990.5408	0.0028	FSLIPSAISG*#
151	338.8542(+3)	2.06	1013.5392	0.0002	GLREKHFQ
152	546.2400(+2)	21.56	1090.4768	0.0040	DNDIEEPFI/L
153	553.7953(+2)	15.57	1105.5750	0.0006	YAVPEGTLRT*
154	410.9301(+3)	19.75	1229.7669	0.0062	LFKLATKIIPS*
155	439.5852(+3)	11.57	1315.7322	0.0010	NIKEKLTEVKD*
156	354.2274(+4)	6.55	1412.8784	0.0021	KLRSKGKQLLKD*

I/Lmeans Leucine or Isolucine. I/L refers to Leucine or Isoleucine. The identified I/L in the sequence confirmed by ETD-HCD-MS3 are indicated with #, those confirmed by HCD-MSn are indicated with ◆, and those identified through Mascot search are denoted with *.

Supplementary Table 2. De novo sequence peptides matching with the sequences of MASCOT research.

Mass	De novo sequence	MASCOT sequence	Range	Matched Toxins
650.3698	I/LI/LKDY	LLKDY	47-52	BmKbpp
672.452	I/LKKVW	LKKVW	29-33	BmKbpp
830.5062	VWKSKI/LA	VWKSKLA	42-48	BmKbpp
887.5524	LKKVWKS	LKKVWKS	29-35	BmKbpp
1412.887	KI/LRSKGKQI/LI/LKD	KLRSKGKQLLKD	40-51	BmKbpp
556.3673	KI/LI/LPS	KIIPS	34-38	marcin-18
641.411	ATKI/LI/LP	ATKIIP	32-37	marcin-18
728.4546	ATKI/LI/LPS	ATKIIPS	32-38	marcin-18
754.4922	I/LATKI/LI/LP	LATKIIP	31-37	marcin-18
841.5322	I/LATKI/LI/LPS	LATKIIPS	31-38	marcin-18
954.6122	I/LATKI/LIPSI/L	LATKIIPSL	31-39	marcin-18
1229.775	I/LFKLATKI/LI/LPS	LFKLATKIIPS	28-38	marcin-18
499.3072	I/LI/LPSA	LIPSA	27-31	toxin peptide
549.3227	I/LI/LSAF	LISAF	35-39	toxin peptide
676.4122	I/LI/LSAFK-NH_2	LISAFK-NH_2	35-40	toxin peptide
756.4463	I/LI/LPSAI/LSG	LIPSAISG	27-34	toxin peptide
843.478	SI/LI/LPSAI/LSG	SLIPSAISG	26-34	toxin peptide
698.4399	I/LI/LPSAI/LS-NH_2	LIPSAIS-NH_2	27-33	toxin peptide
990.5486	FSLIPSAI/LSG	FSLIPSAISG	25-34	toxin peptide
428.281	I/LGAVA-NH_2	IGAVA-NH_2	25-29	BmKn1
505.2924	FI/LGAV	FIGAV	24-28	BmKn1
575.3528	FI/LGAVA-NH_2	FIGAVA-NH_2	24-29	BmKn1
633.3594	FI/LGAVAG	FIGAVAG	24-30	BmKn1
544.3302	I/LI/LQTA	IIQTA	42-46	BmTXK-beta-2
780.4554	I/LLQTAVH	IIQTAVH	42-48	BmTXK-beta-2
885.4893	GLREKHF	GLREKHF	20-27	BmTXK-beta-2
908.5548	I/LI/LQTAVKH	IIQTAVHK	42-49	BmTXK-beta-2
1013.534	GI/LREKHFQ	GLREKHFQ	20-28	BmTXK-beta-2
1105.583	YAVPEGTI/LRT	YAVPEGTLRT	32-41	BmTXK-beta-2
617.3624	I/LMKNI/L	IMKNI	28-32	BmTXK-beta
874.509	I/LMKNI/LKE	IMKNIKE	28-34	BmTXK-beta
1315.741	NI/LKEKI/LTEVKD	NIKEKLTEVKD	31-41	BmTXK-beta

I/L: Leucine or Isolucine

Supplementary Figure 1~156

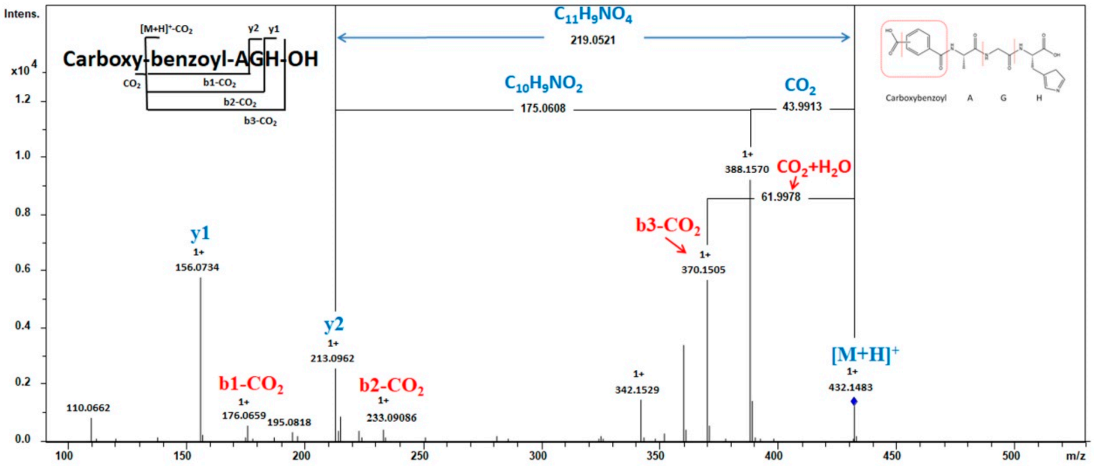

Supplementary Figure 1. Mass spectra and de novo sequencing analysis of carboxybenzoyl-AGH.

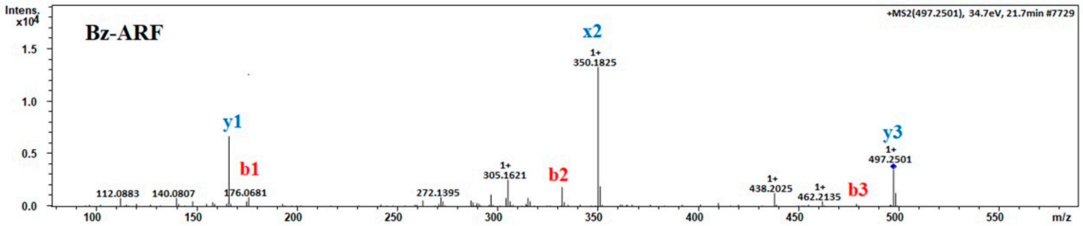

Supplementary Figure 2. Mass spectra and de novo sequencing analysis of Bz-ARF-NH2

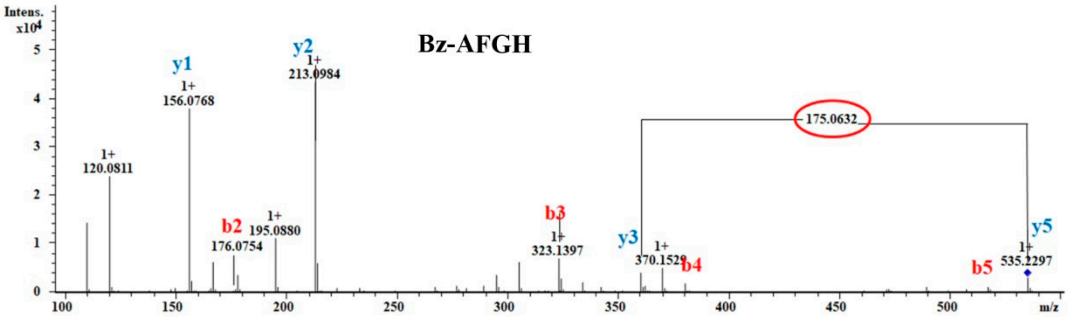

Supplementary Figure 3. Mass spectra and de novo sequencing analysis of Bz-AFGH

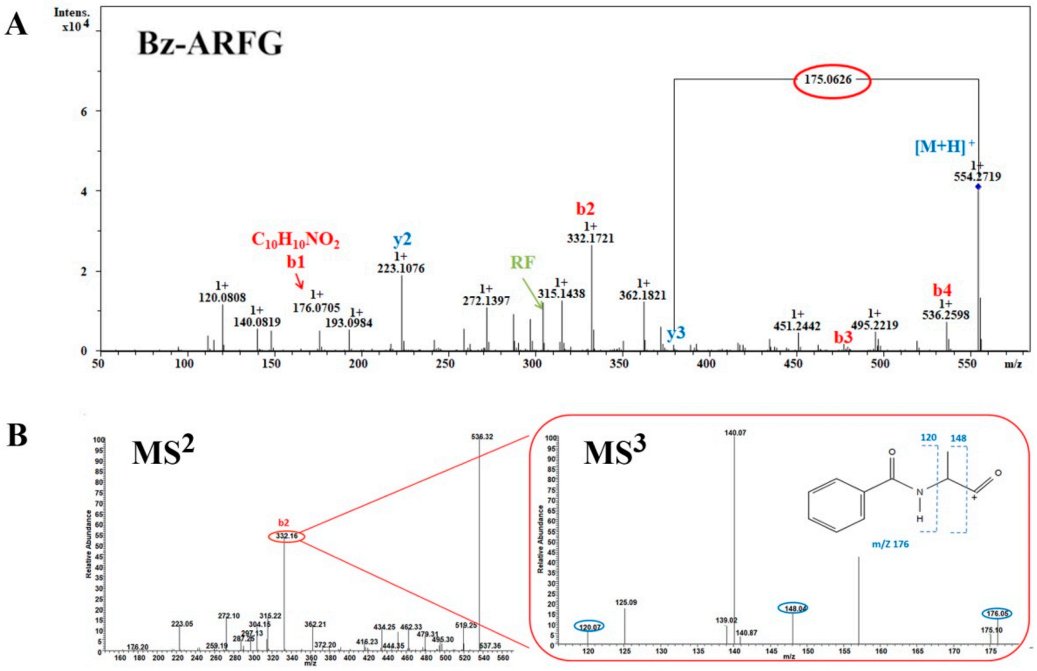

Supplementary Figure 4. Mass spectra and de novo sequencing analysis of Bz-ARFG

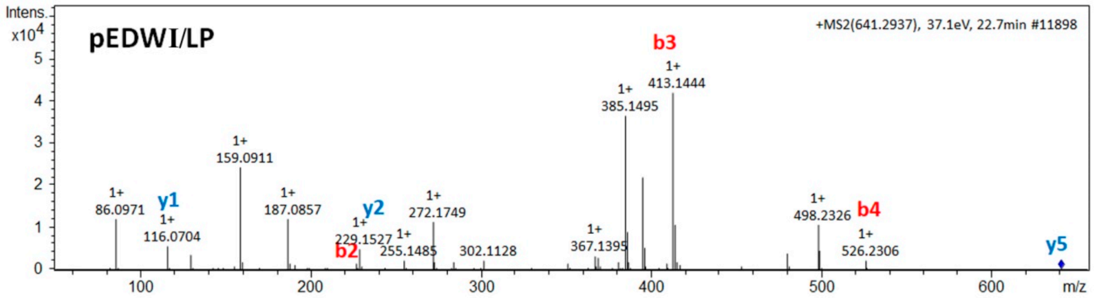

Supplementary Figure 5. Mass spectra and de novo sequencing analysis of pEDWI/LP

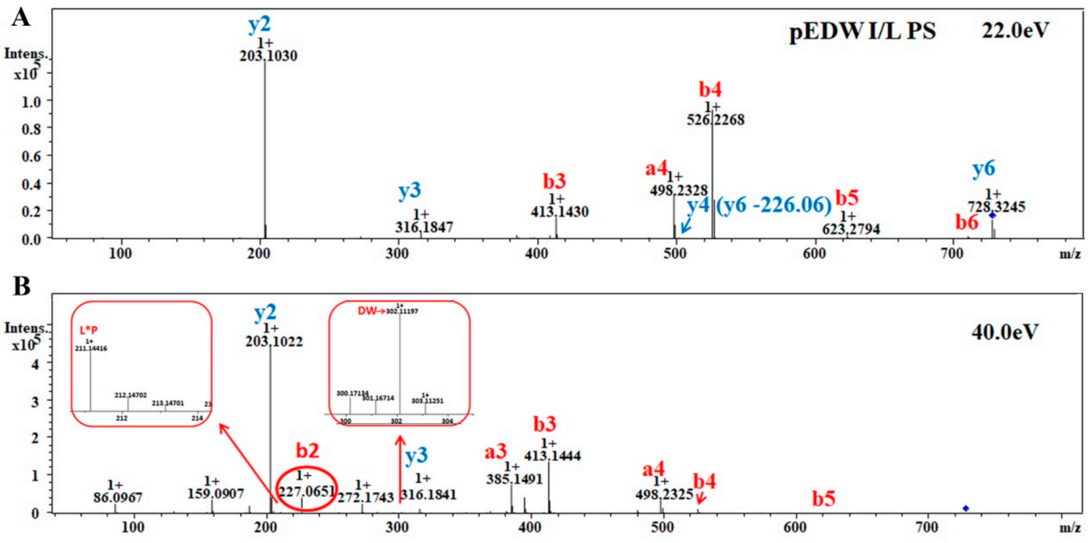

Supplementary Figure 6. Mass spectra and de novo sequencing analysis of pEDWI/LPS

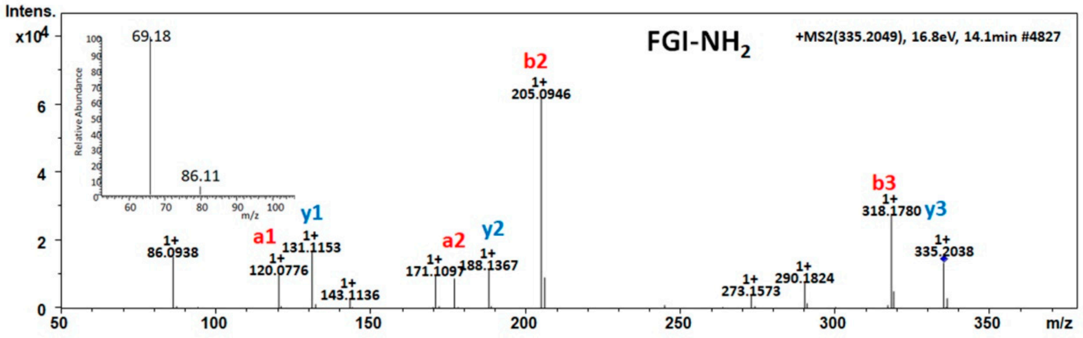

Supplementary Figure 7. Mass spectra and de novo sequencing analysis of FGI-NH2

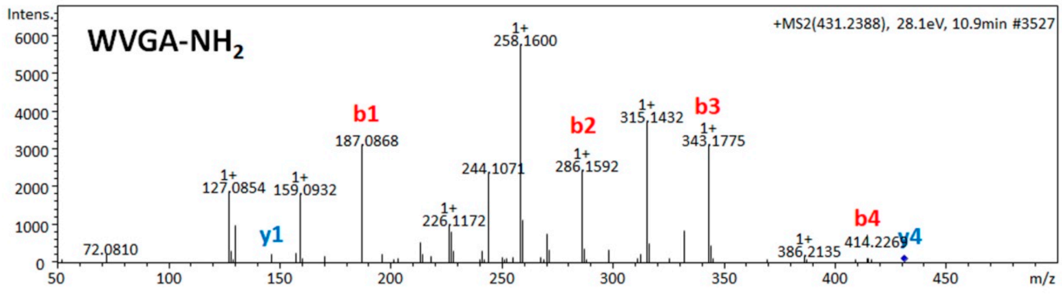

Supplementary Figure 8. Mass spectra and de novo sequencing analysis of WVGA-NH2

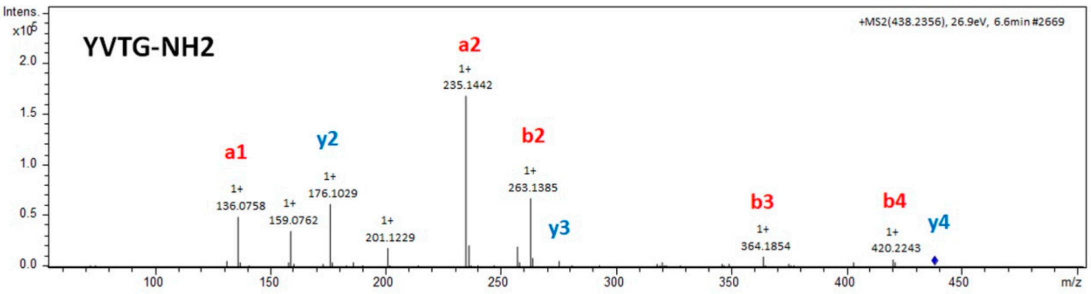

Supplementary Figure 9. Mass spectra and de novo sequencing analysis of

YVTG-NH2

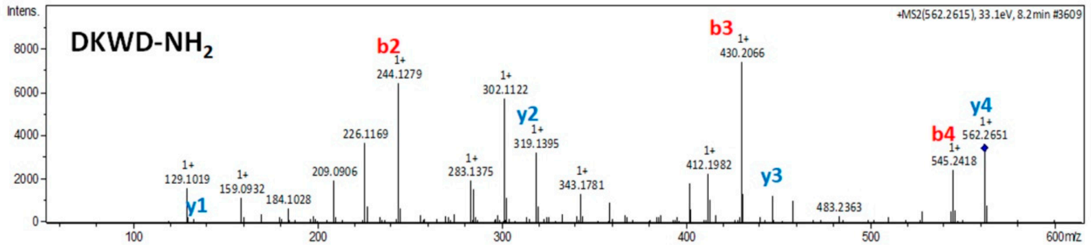

Supplementary Figure 10. Mass spectra and de novo sequencing analysis of

DKWD-NH2

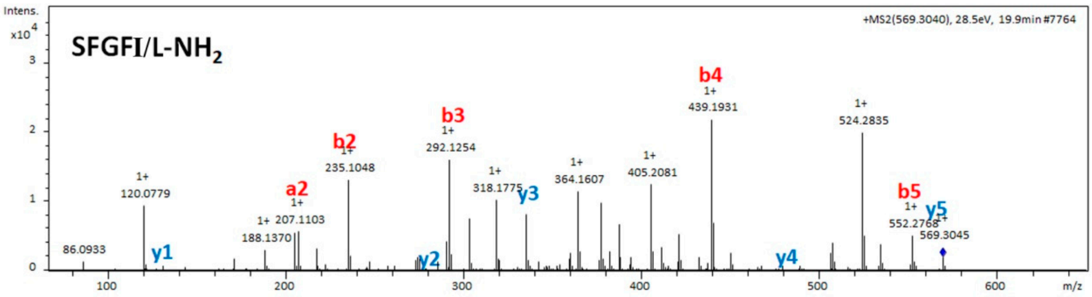

Supplementary Figure 11. Mass spectra and de novo sequencing analysis of

SFGFI/L-NH2

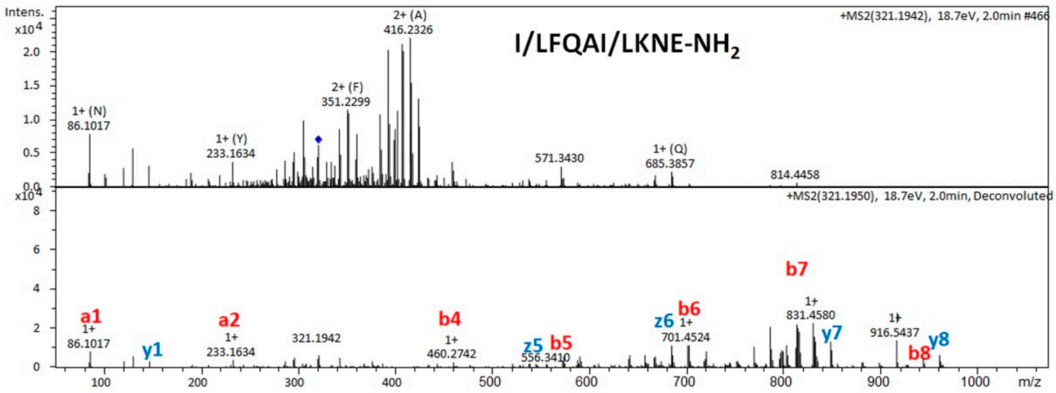

Supplementary Figure 12. Mass spectra and de novo sequencing analysis of

I/LFQAI/LKNE-NH_2

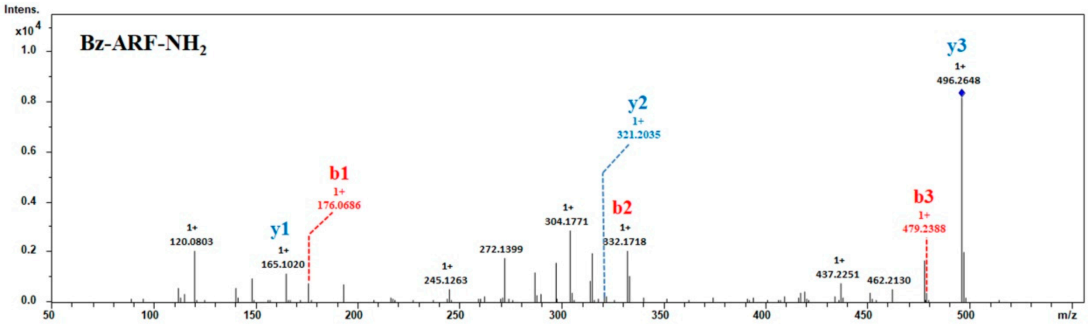

Supplementary Figure 13. Mass spectra and de novo sequencing analysis of

Bz-ARF-NH_2

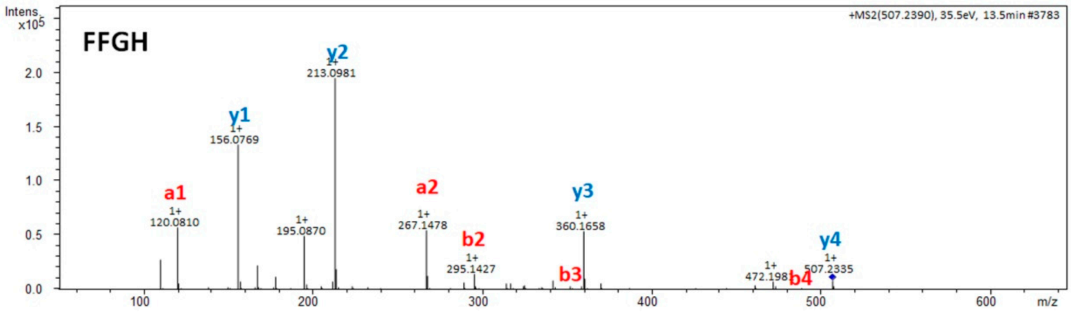

Supplementary Figure 14. Mass spectra and de novo sequencing analysis of

FFGH

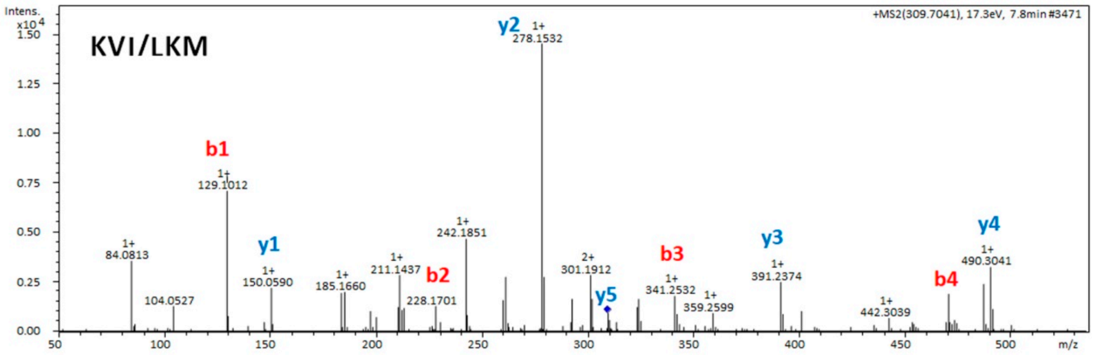

Supplementary Figure 15. Mass spectra and de novo sequencing analysis of KVI/LKM

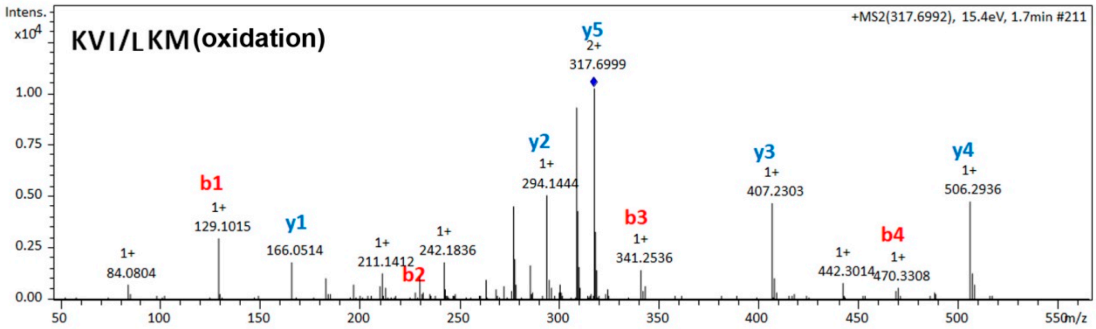

Supplementary Figure 16. Mass spectra and de novo sequencing analysis of KVI/LKM(oxidation)

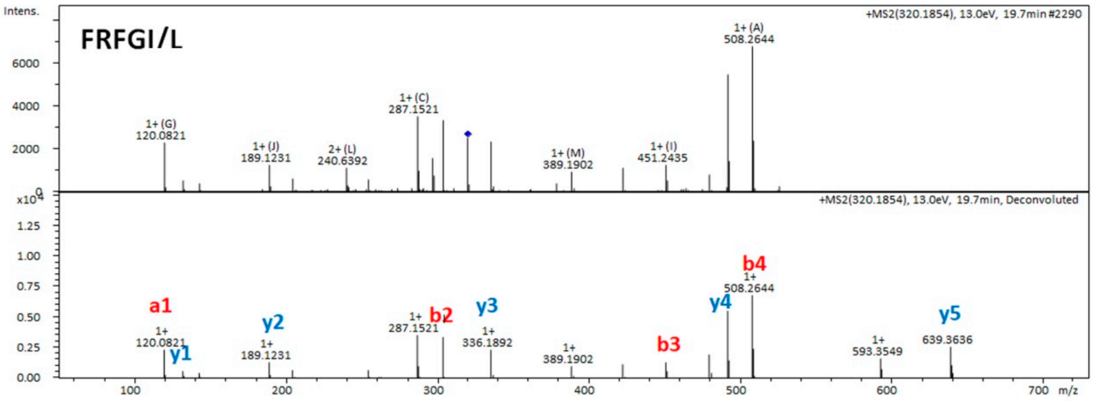

Supplementary Figure 17. Mass spectra and de novo sequencing analysis of

FRFGI/L

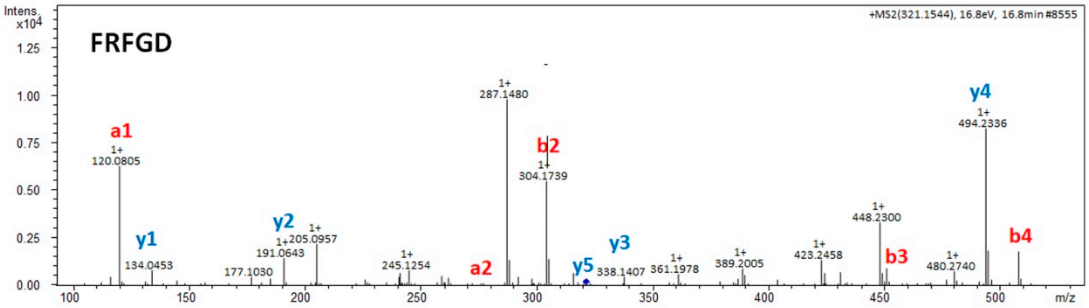

Supplementary Figure 18. Mass spectra and de novo sequencing analysis of FRFGD

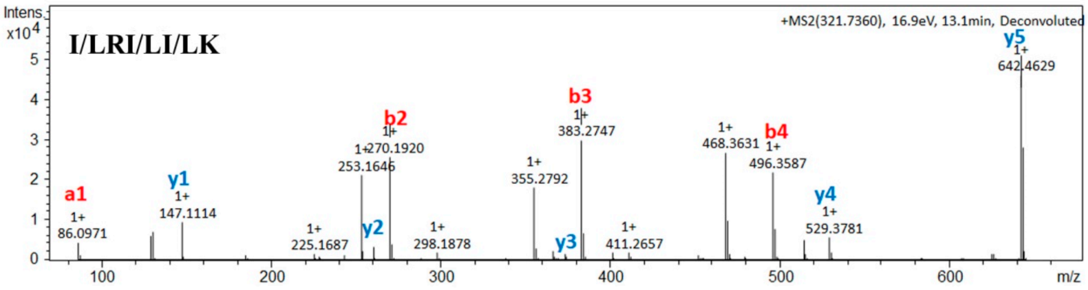

Supplementary Figure 19. Mass spectra and de novo sequencing analysis of I/LRI/LI/LK

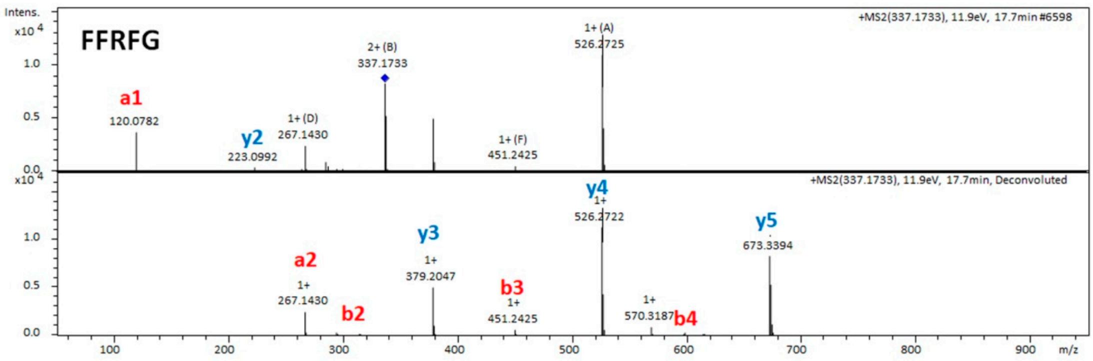

Supplementary Figure 20. Mass spectra and de novo sequencing analysis of

FFRFG

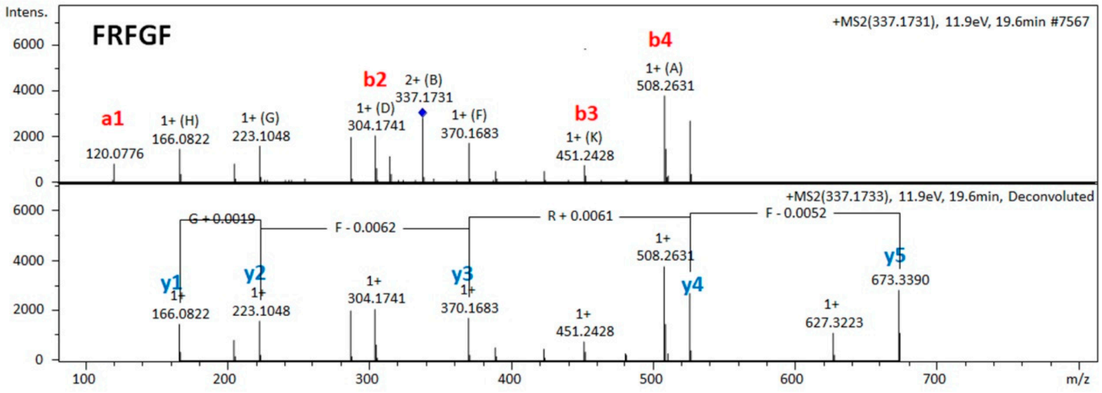

Supplementary Figure 21. Mass spectra and de novo sequencing analysis of

FRFGF

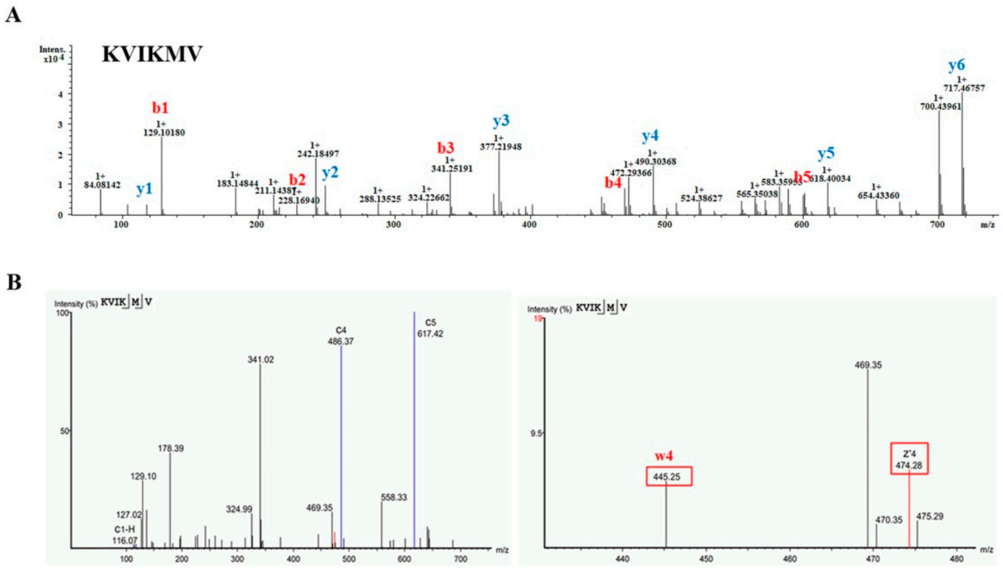

Supplementary Figure 22. Mass spectra and de novo sequencing analysis of

KVIKMV

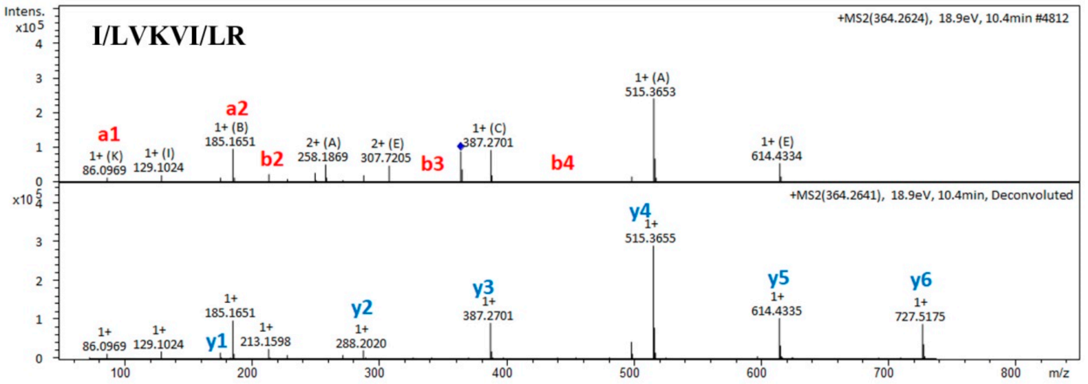

Supplementary Figure 23. Mass spectra and de novo sequencing analysis of I/LVKVI/LR

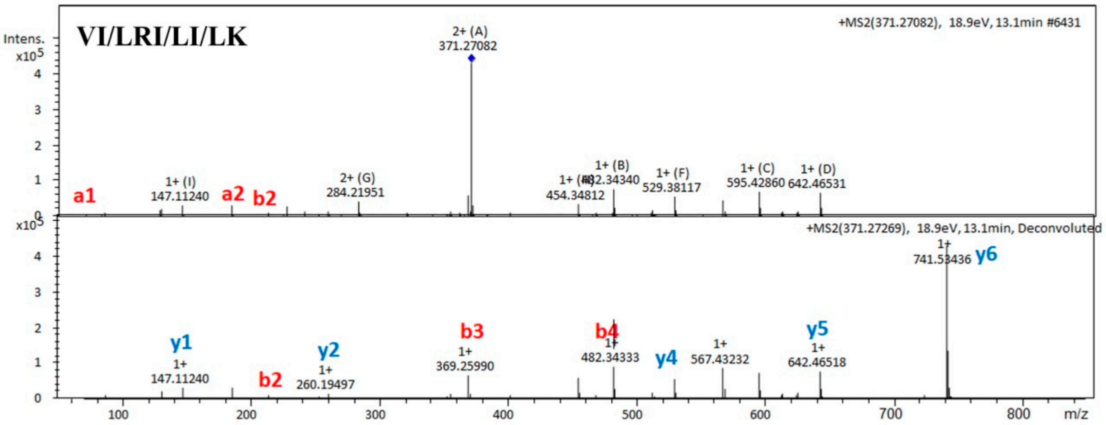

Supplementary Figure 24. Mass spectra and de novo sequencing analysis of VI/LRI/L/I/LK

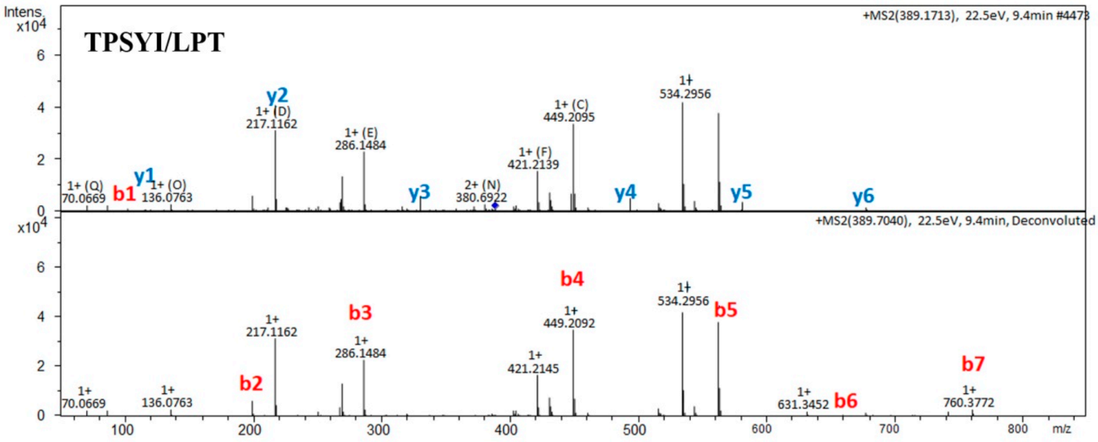

Supplementary Figure 25. Mass spectra and de novo sequencing analysis of

TPSYI/LPT

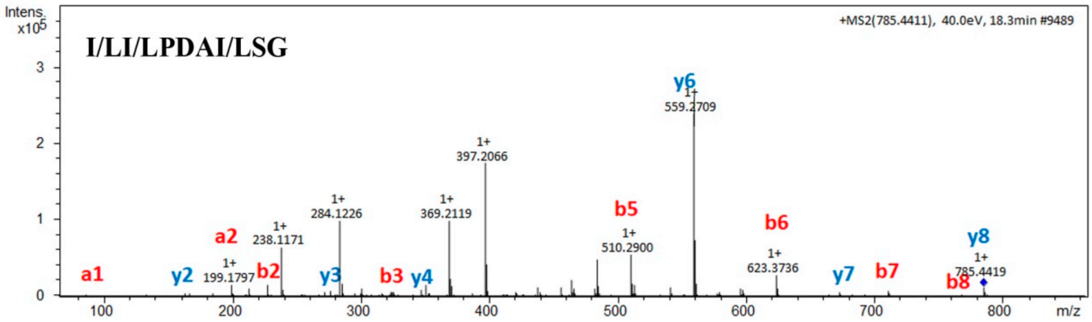

Supplementary Figure 26. Mass spectra and de novo sequencing analysis of

I/LI/LPDAI/LSG

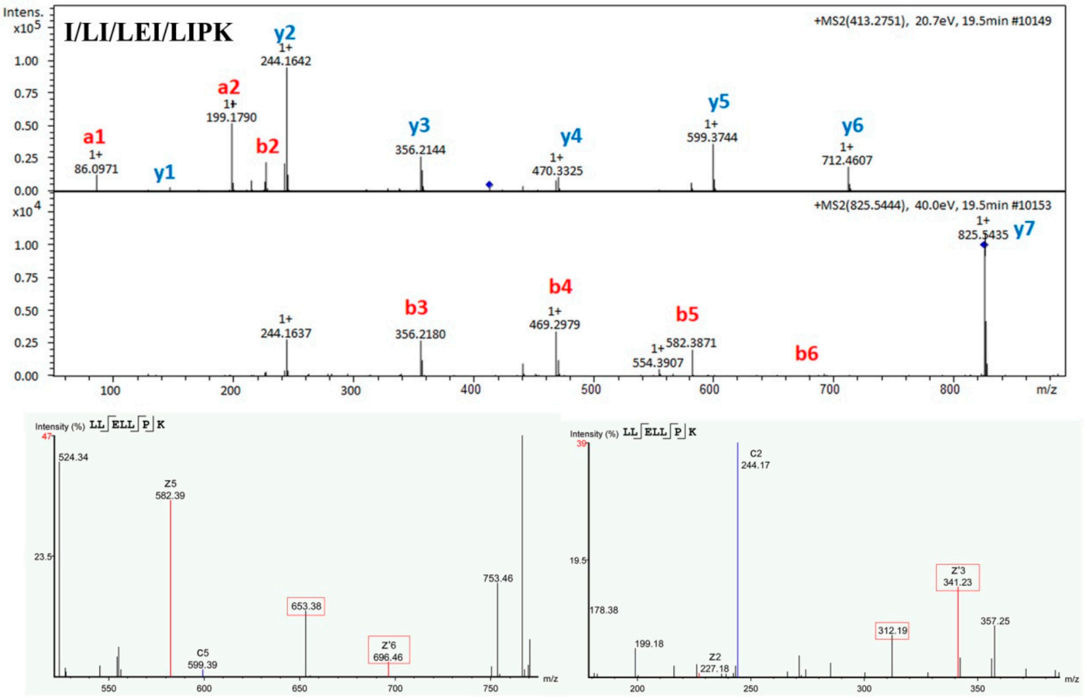

Supplementary Figure 27. Mass spectra and de novo sequencing analysis of I/LI/LEI/LIPK

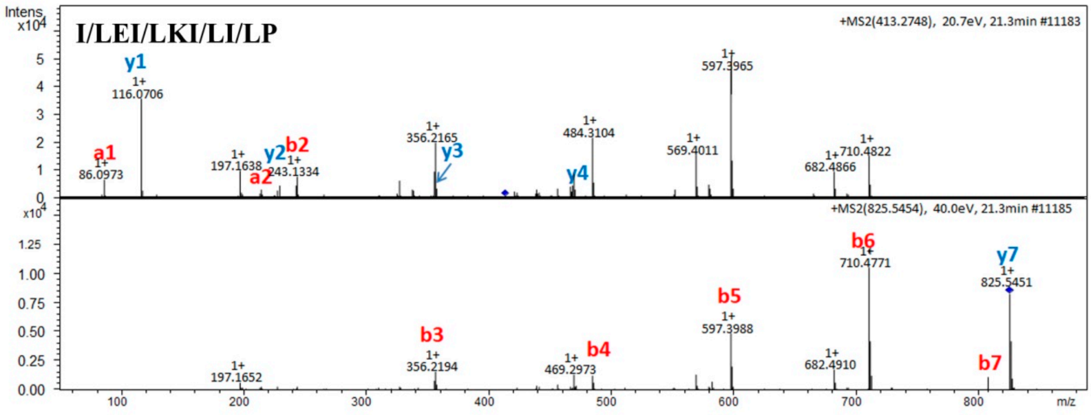

Supplementary Figure 28. Mass spectra and de novo sequencing analysis of I/LEI/LKI/LI/LP

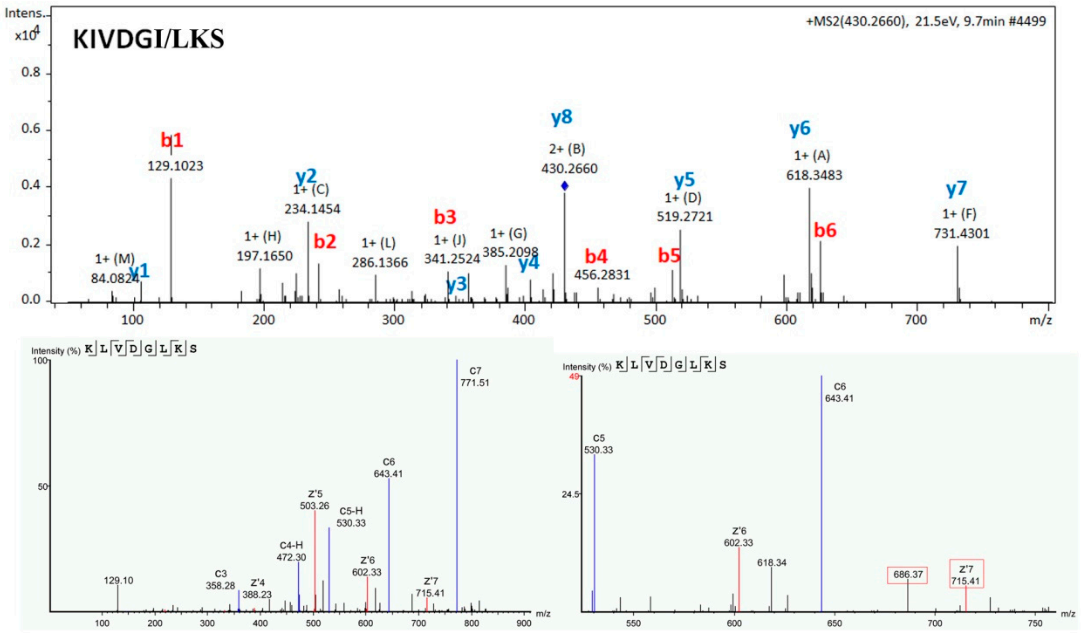

Supplementary Figure 29. Mass spectra and de novo sequencing analysis of KIVDGI/LKS

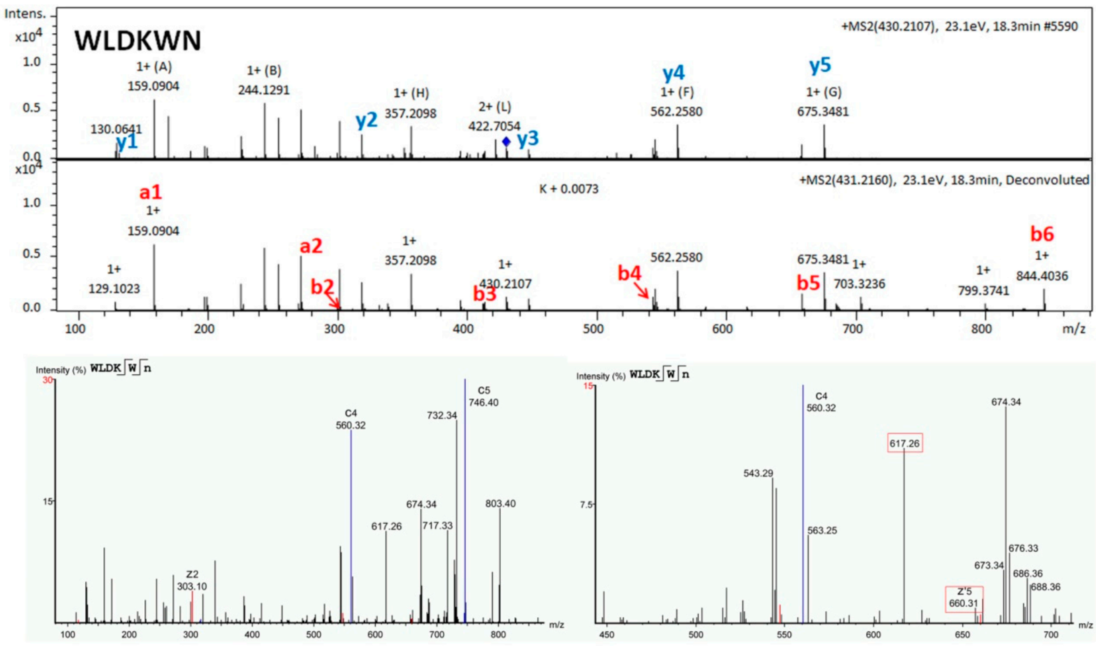

Supplementary Figure 30. Mass spectra and de novo sequencing analysis of WLDKWN

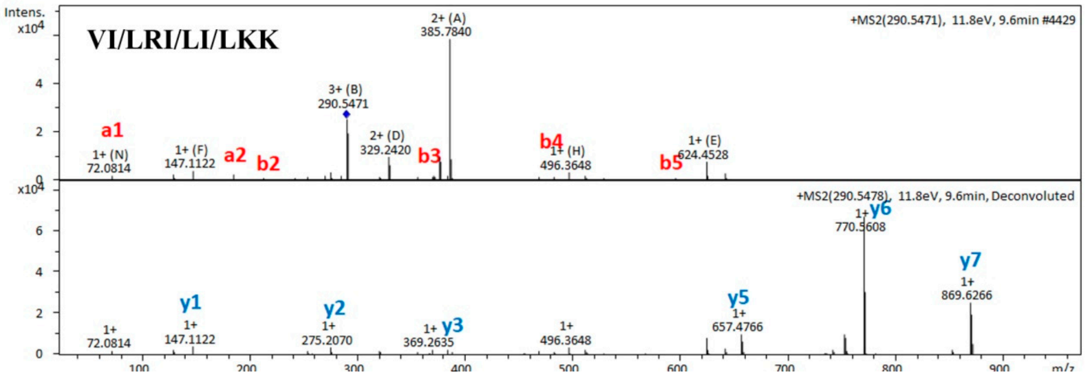

Supplementary Figure 31. Mass spectra and de novo sequencing analysis of VI/LRI/LI/LKK

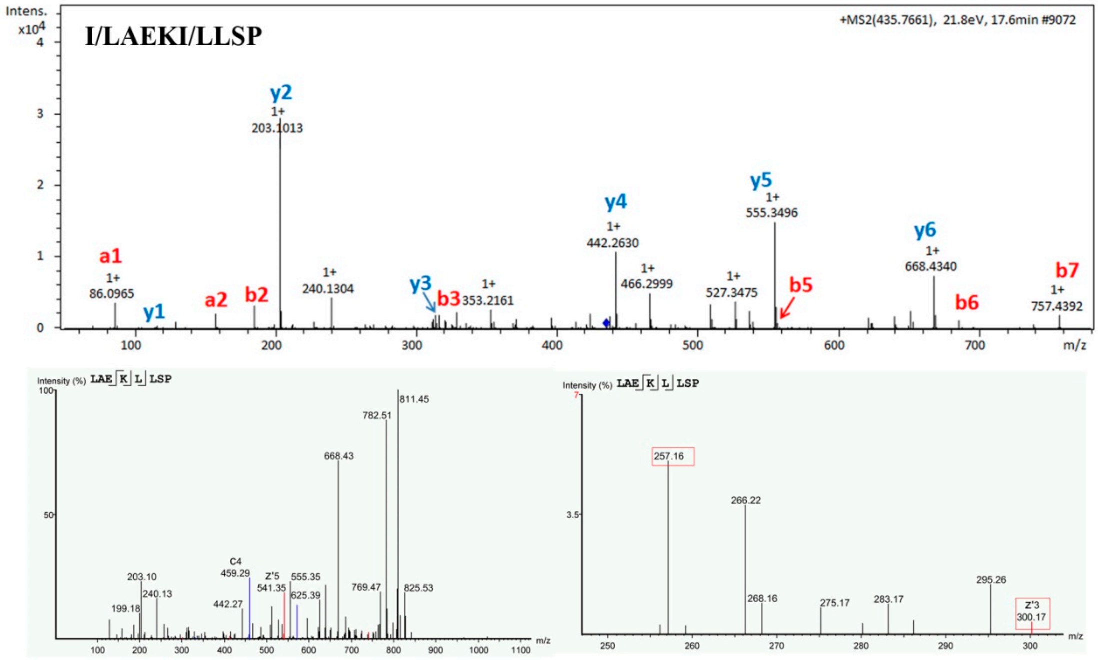

Supplementary Figure 32. Mass spectra and de novo sequencing analysis of I/LAEKI/LLSP

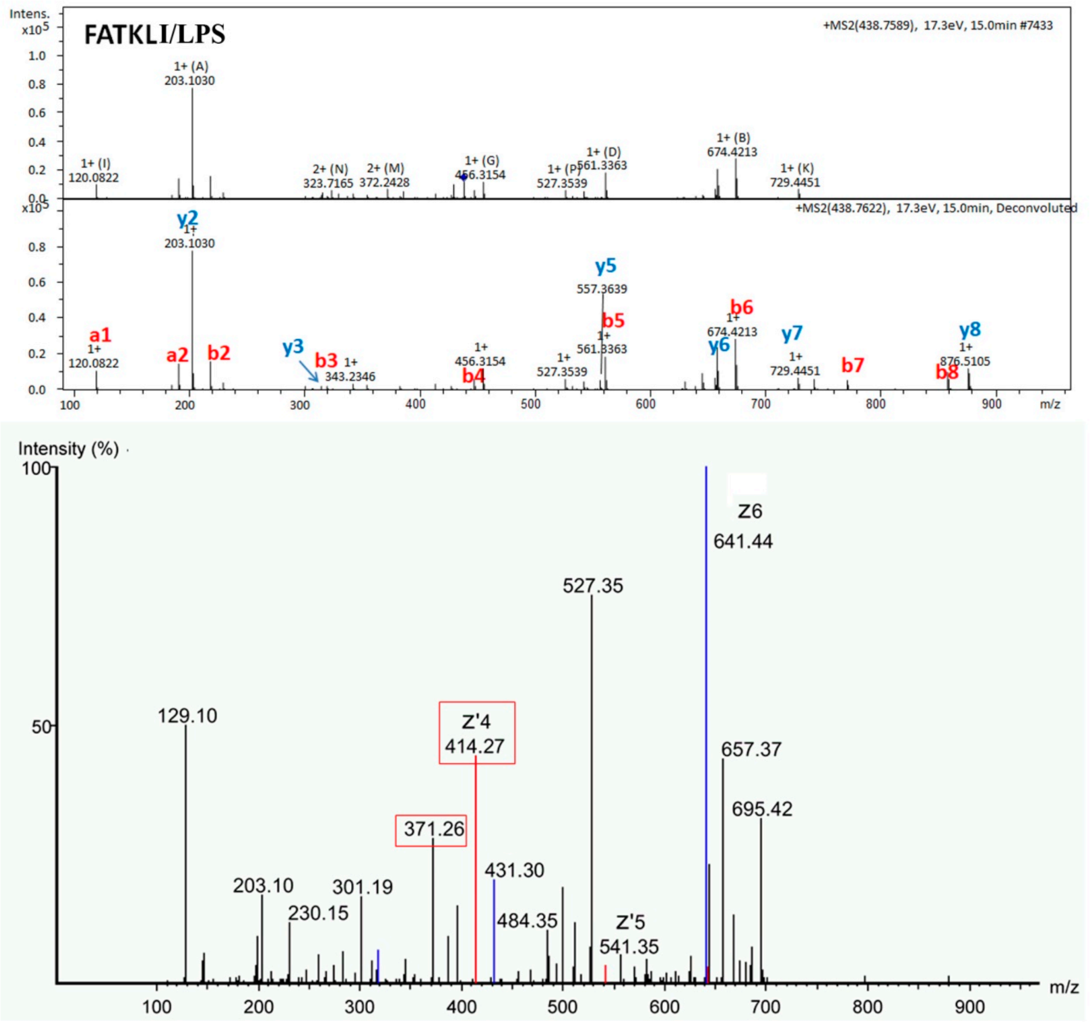

Supplementary Figure 33. Mass spectra and de novo sequencing analysis of FATKLI/LPS

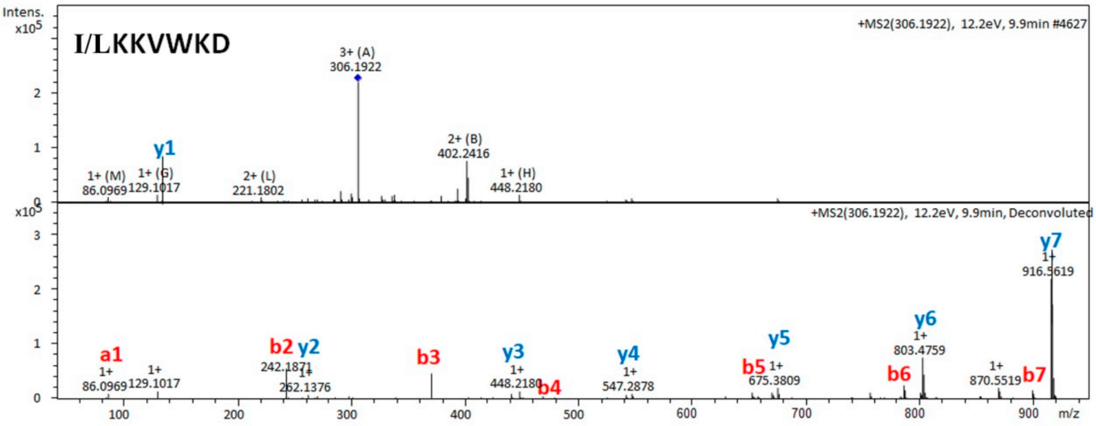

Supplementary Figure 34. Mass spectra and de novo sequencing analysis of

I/LKKVWKD

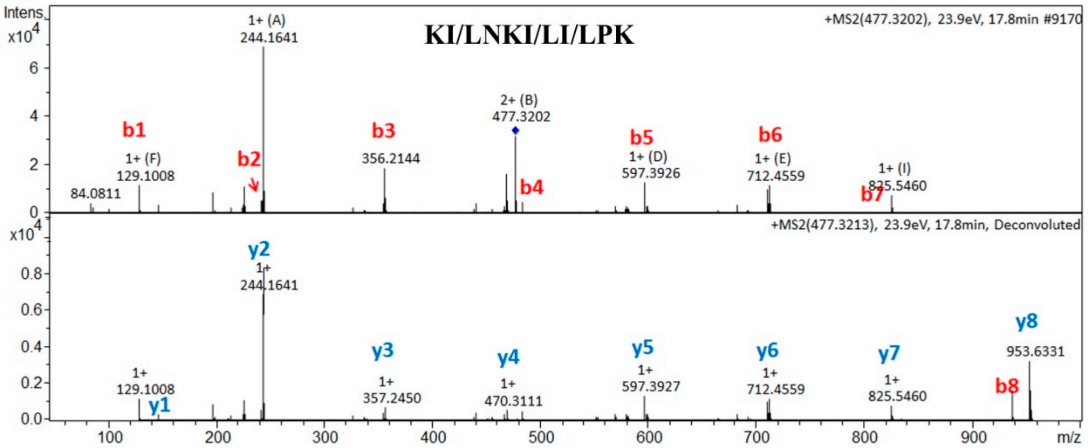

Supplementary Figure 35. Mass spectra and de novo sequencing analysis of KI/LNKI/LI/LPK

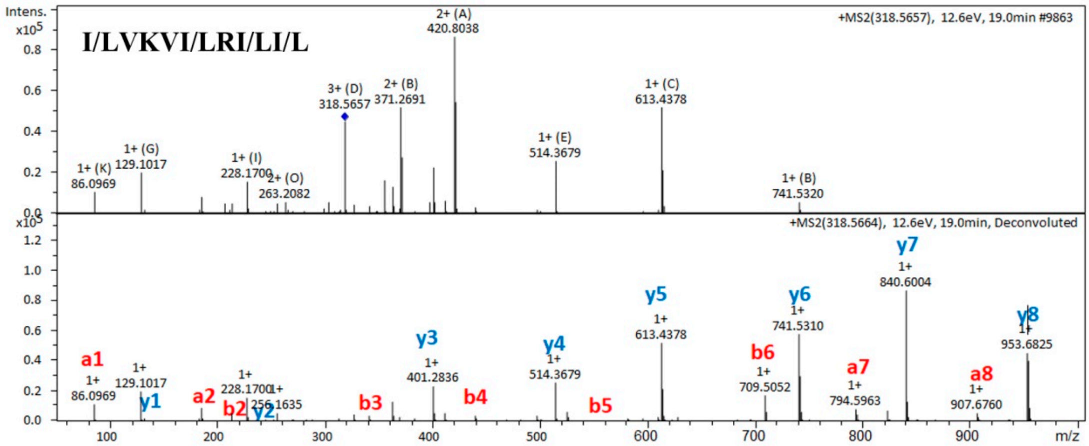

Supplementary Figure 36. Mass spectra and de novo sequencing analysis of I/LVKVI/LRI/LI/L

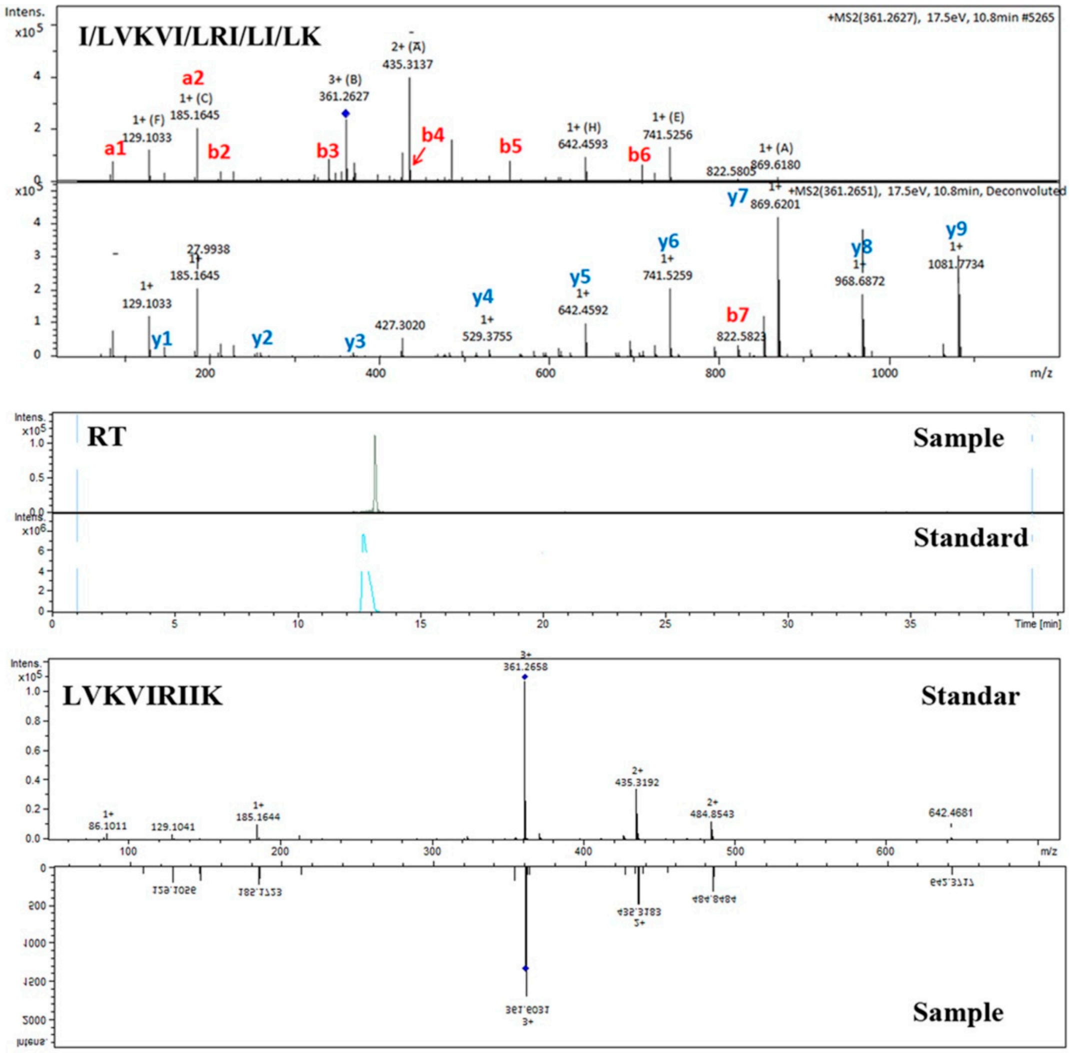

Supplementary Figure 37. Mass spectra and de novo sequencing analysis of I/LVKVI/LRI/LI/LK

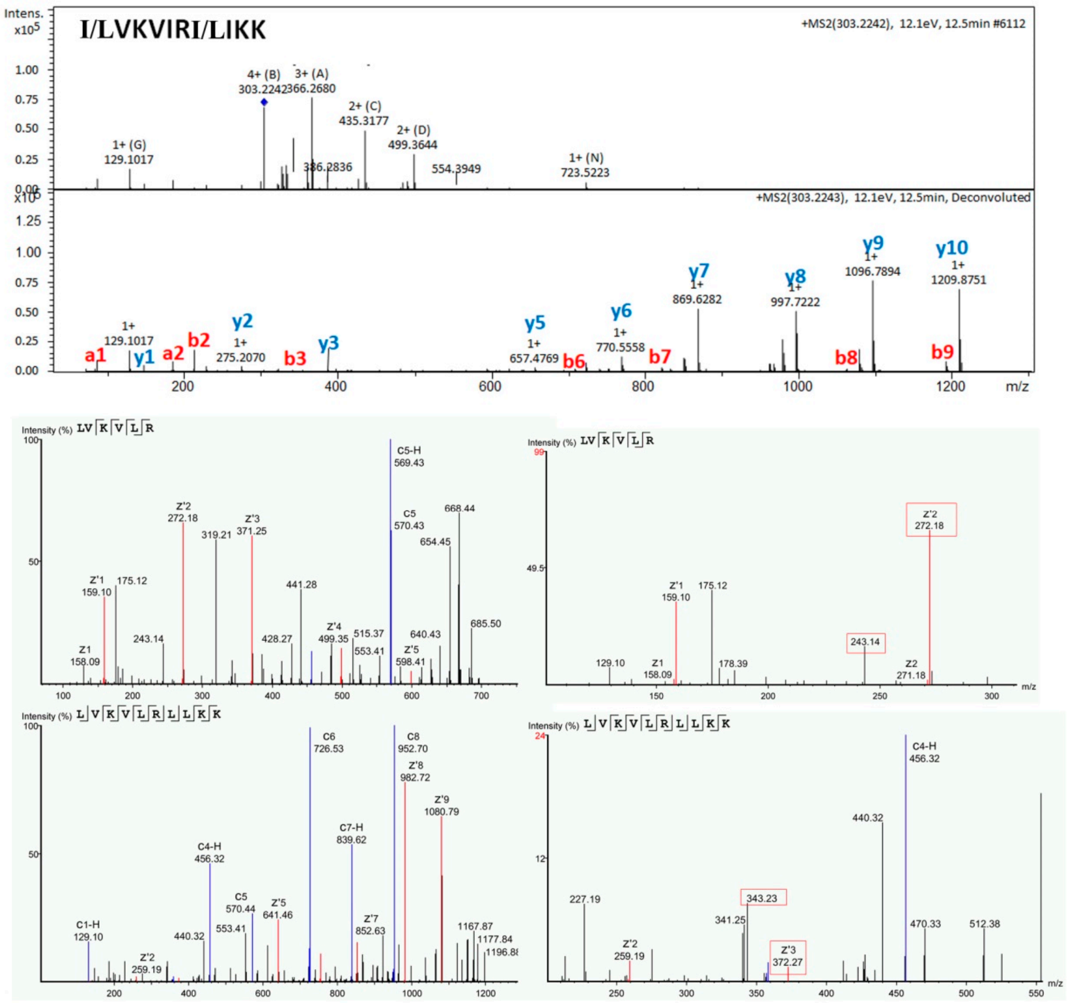

Supplementary Figure 38. Mass spectra and de novo sequencing analysis of I/LVKVIRI/LIKK

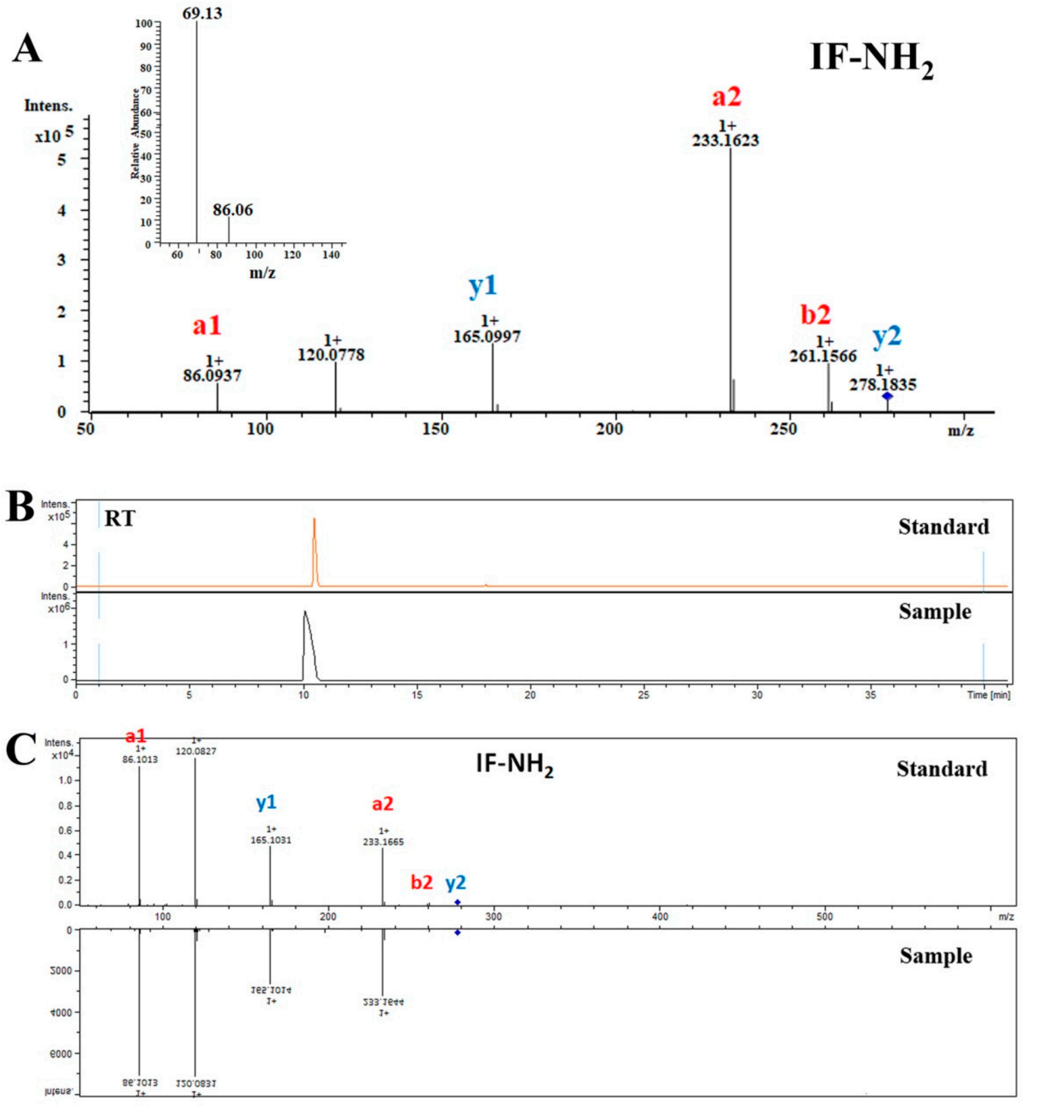

Supplementary Figure 39. Mass spectra and de novo sequencing analysis of IF-NH2

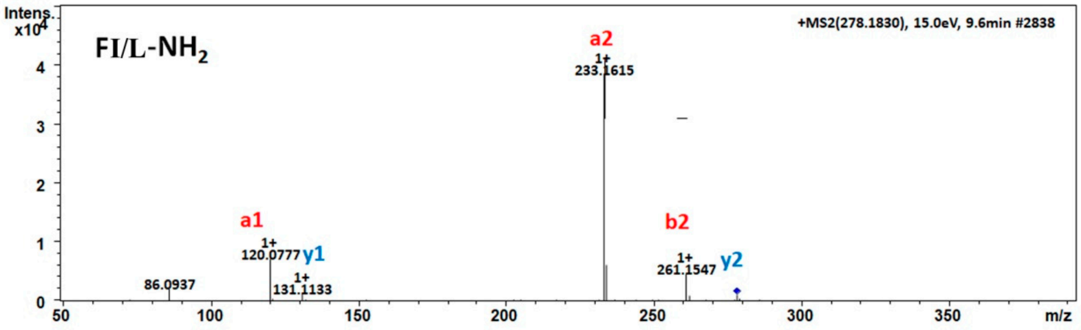

Supplementary Figure 40. Mass spectra and de novo sequencing analysis of

FI/L-NH2

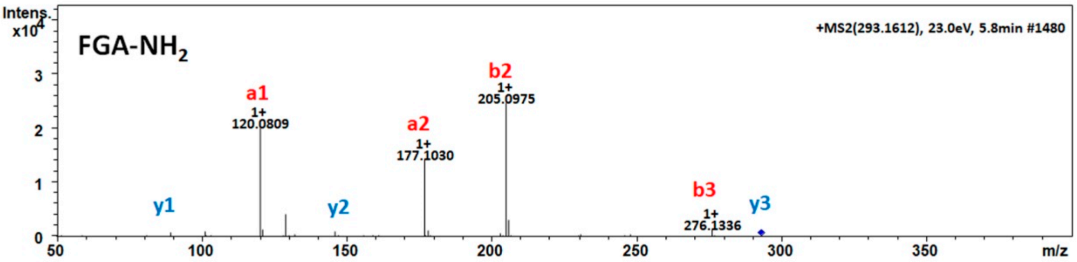

Supplementary Figure 41. Mass spectra and de novo sequencing analysis of FGA-NH2

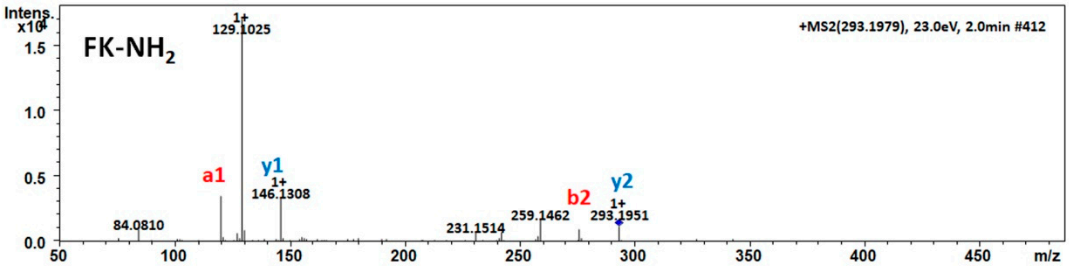

Supplementary Figure 42. Mass spectra and de novo sequencing analysis of FK-NH2

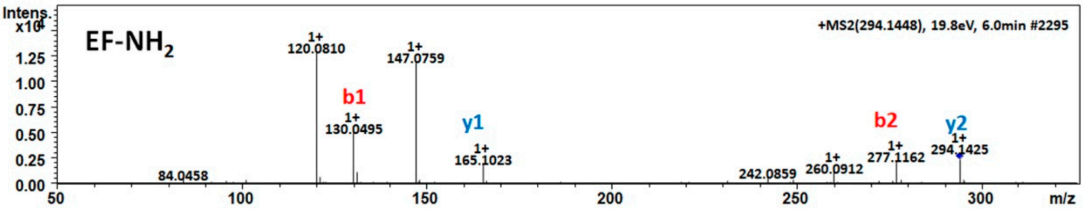

Supplementary Figure 43. Mass spectra and de novo sequencing analysis of EF-NH2

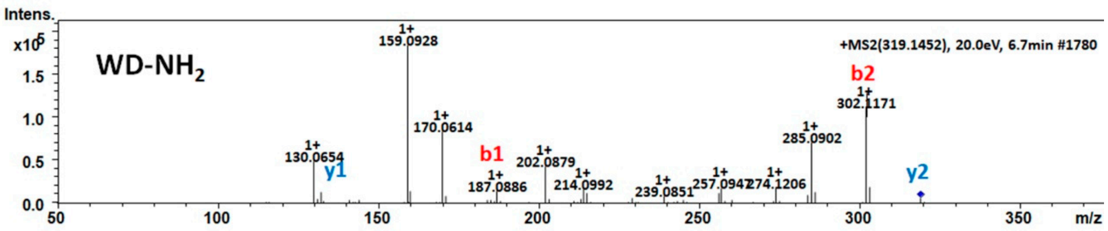

Supplementary Figure 44. Mass spectra and de novo sequencing analysis of WD-NH$_2$

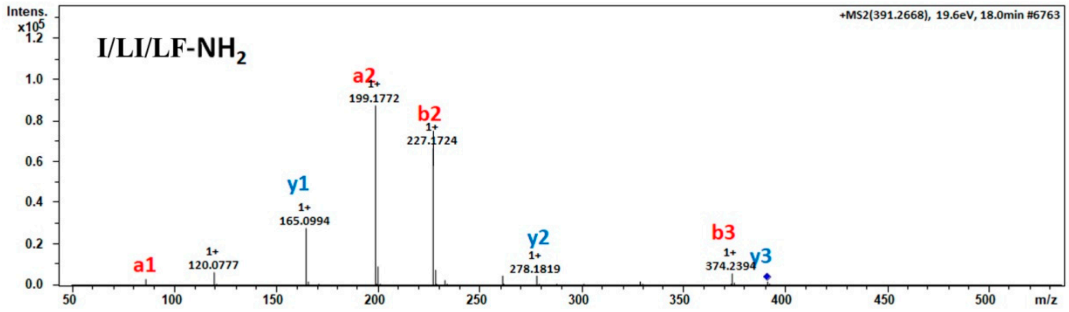

Supplementary Figure 45. Mass spectra and de novo sequencing analysis of I/LI/LF-NH$_2$

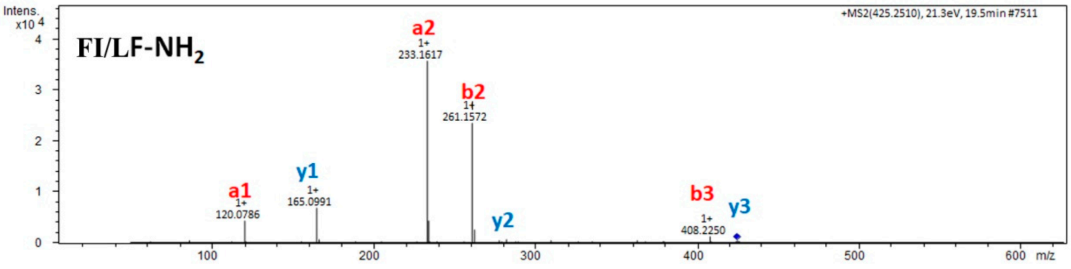

Supplementary Figure 46. Mass spectra and de novo sequencing analysis of FI/LF-NH$_2$

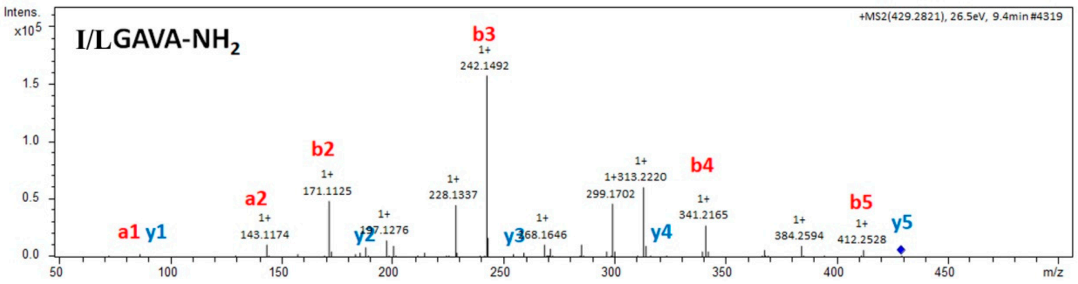

Supplementary Figure 47. Mass spectra and de novo sequencing analysis of I/LGAVA-NH2

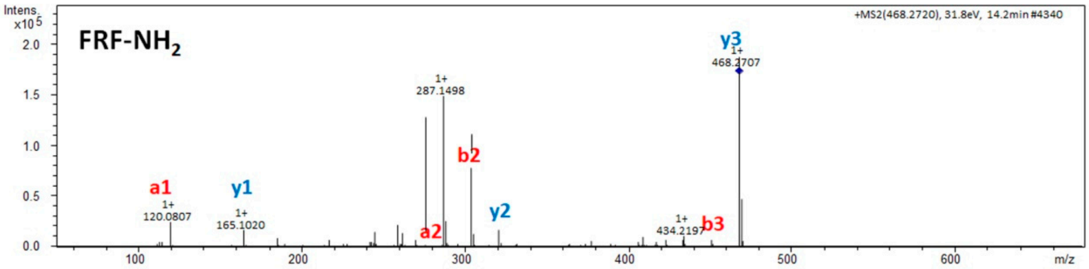

Supplementary Figure 48. Mass spectra and de novo sequencing analysis of FRF-NH2

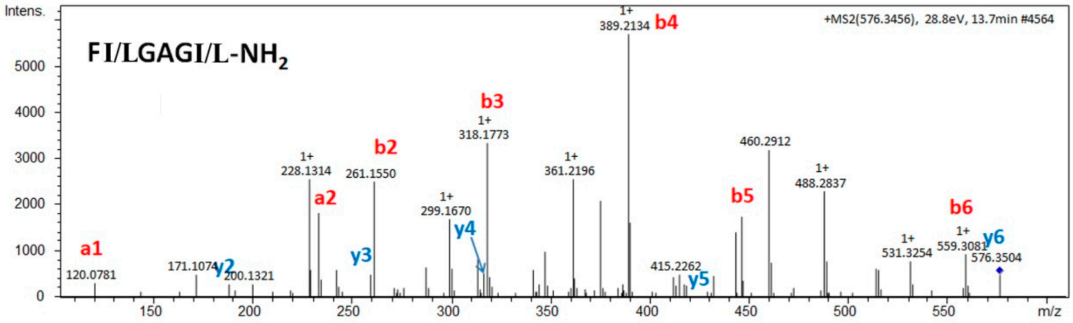

Supplementary Figure 49. Mass spectra and de novo sequencing analysis of FI/LGAGI/L-NH2

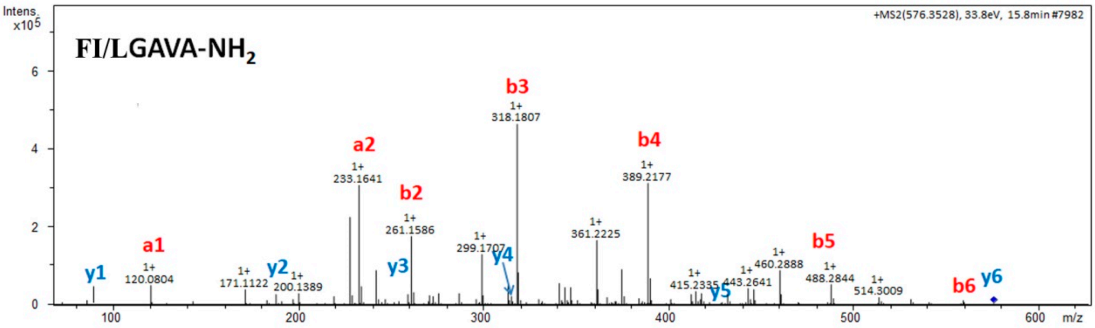

Supplementary Figure 50. Mass spectra and de novo sequencing analysis of FI/LGAVA-NH2

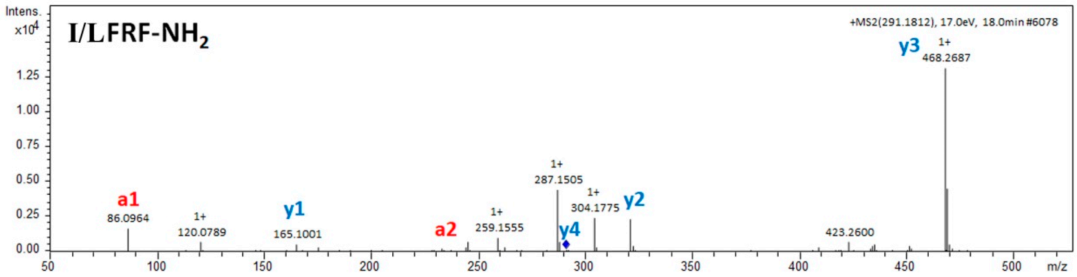

Supplementary Figure 51. Mass spectra and de novo sequencing analysis of I/LFRF-NH2

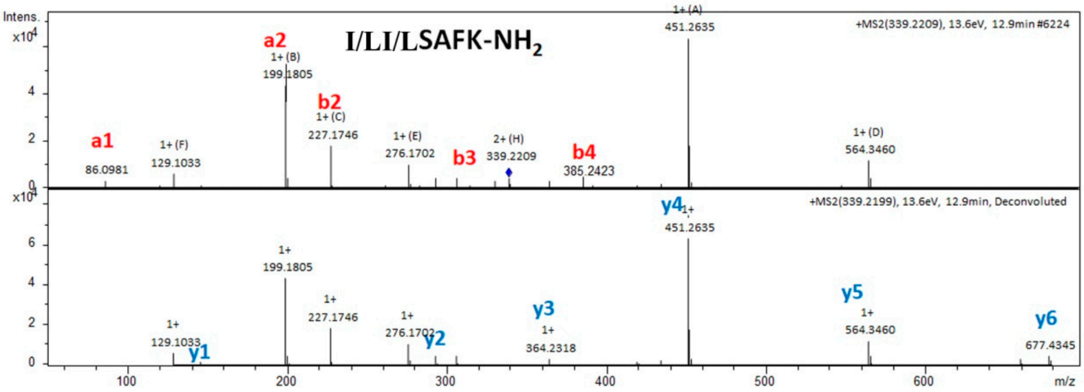

Supplementary Figure 52. Mass spectra and de novo sequencing analysis of I/LI/LSAFK-NH2

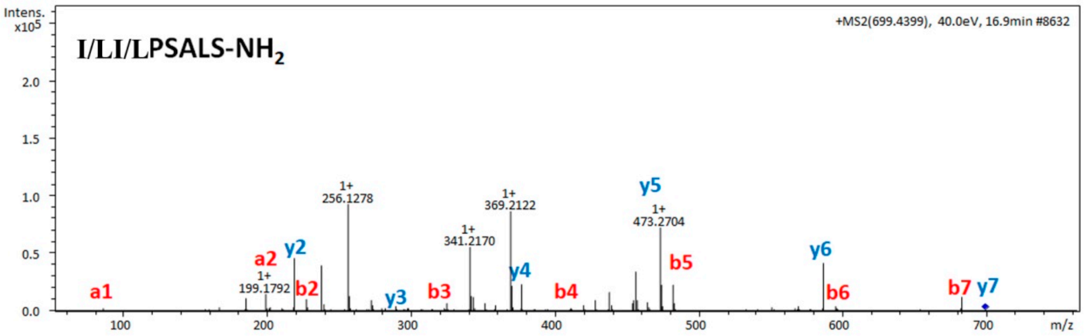

Supplementary Figure 53. Mass spectra and de novo sequencing analysis of I/LI/LPSAI/LS-NH2

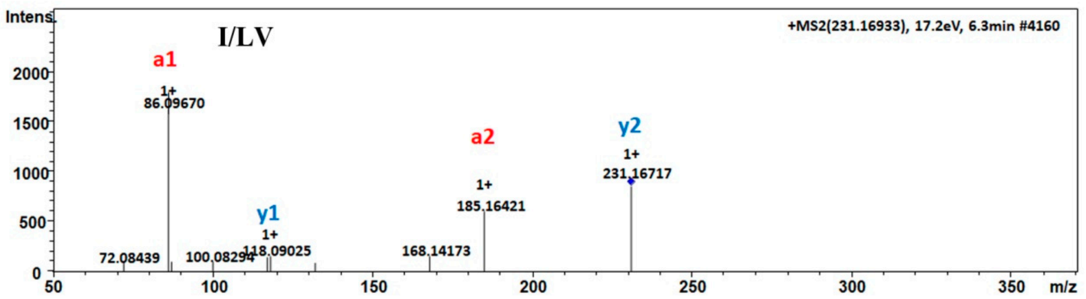

Supplementary Figure 54. Mass spectra and de novo sequencing analysis of I/LV

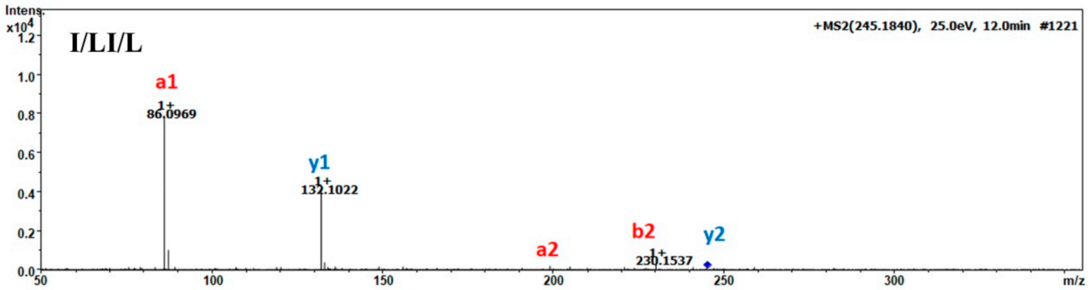

Supplementary Figure 55. Mass spectra and de novo sequencing analysis of I/LI/L

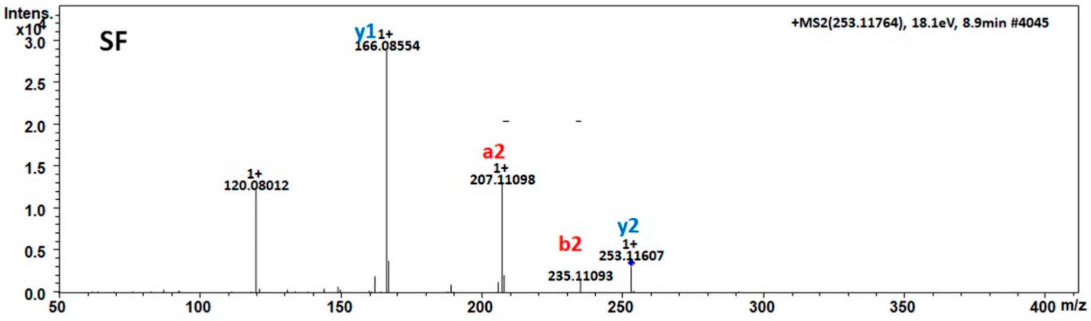

Supplementary Figure 56. Mass spectra and de novo sequencing analysis of SF

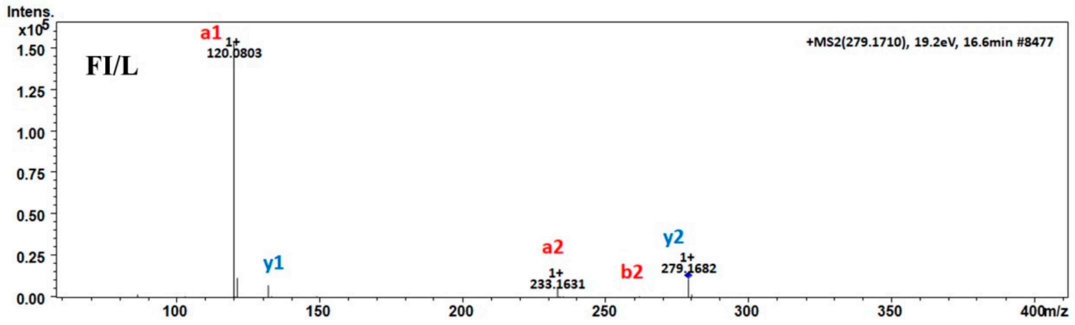

Supplementary Figure 57. Mass spectra and de novo sequencing analysis of FI/L

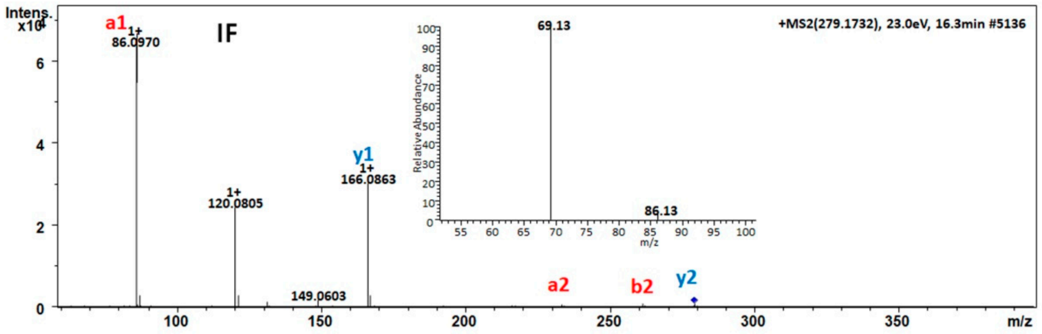

Supplementary Figure 58. Mass spectra and de novo sequencing analysis of IF

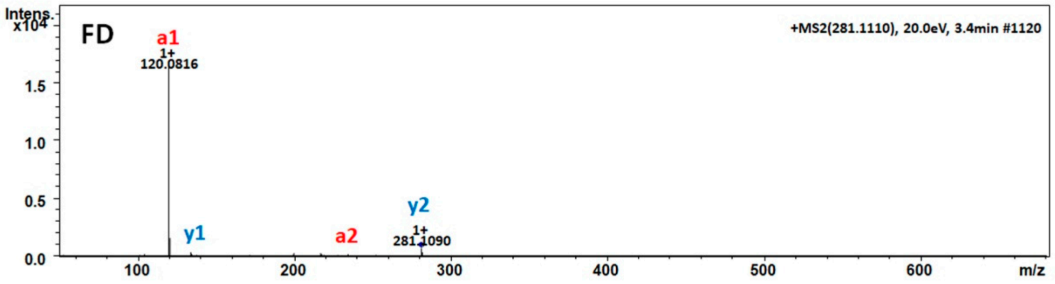

Supplementary Figure 59. Mass spectra and de novo sequencing analysis of FD

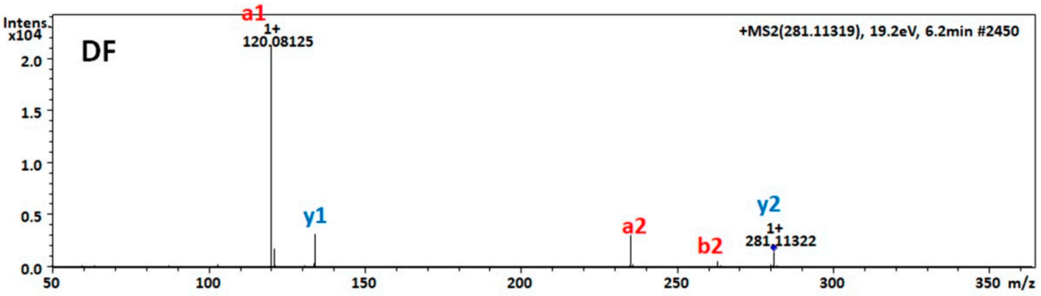

Supplementary Figure 60. Mass spectra and de novo sequencing analysis of DF

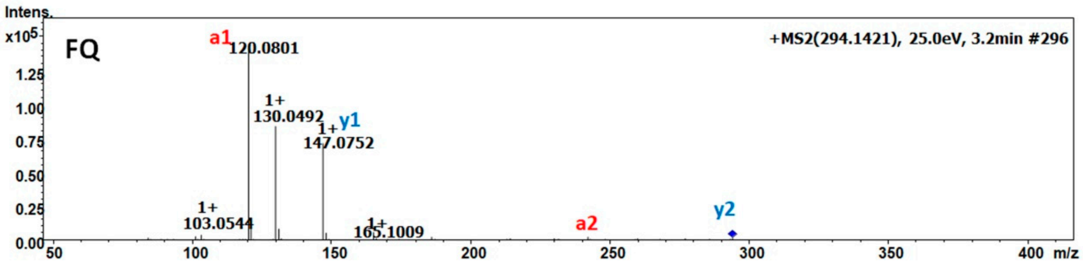

Supplementary Figure 61. Mass spectra and de novo sequencing analysis of FQ

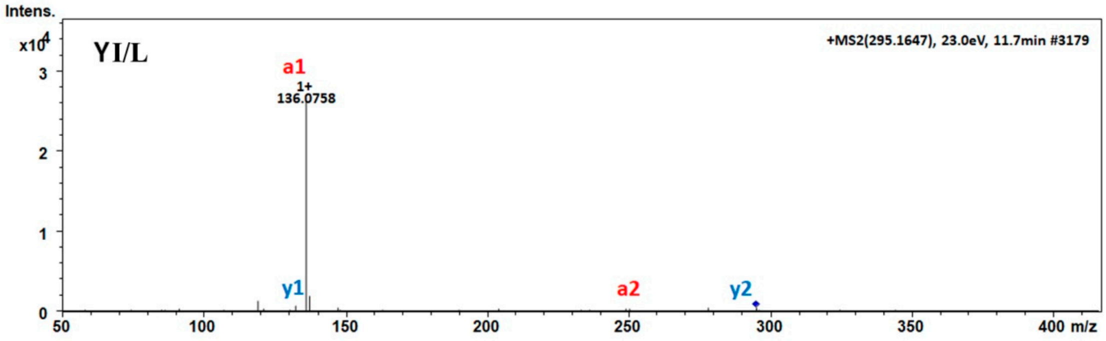

Supplementary Figure 62. Mass spectra and de novo sequencing analysis of YI/L

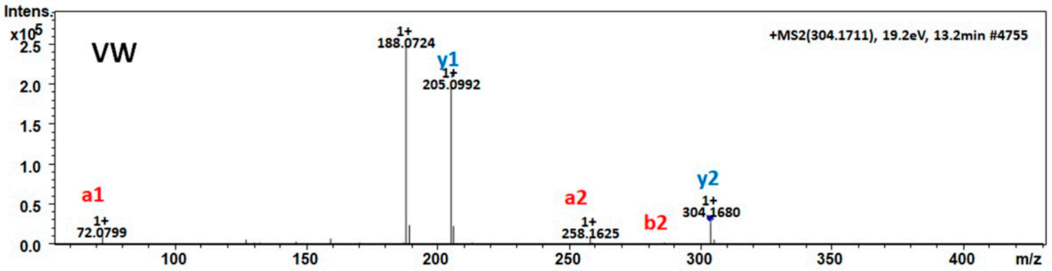

Supplementary Figure 63. Mass spectra and de novo sequencing analysis of VW

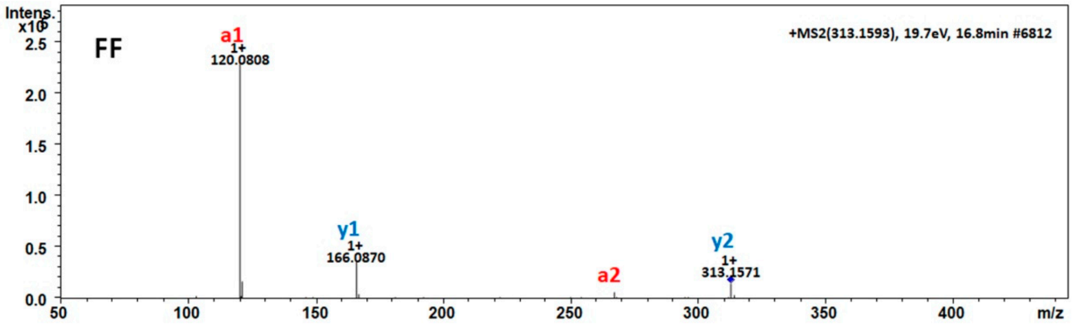

Supplementary Figure 64. Mass spectra and de novo sequencing analysis of FF

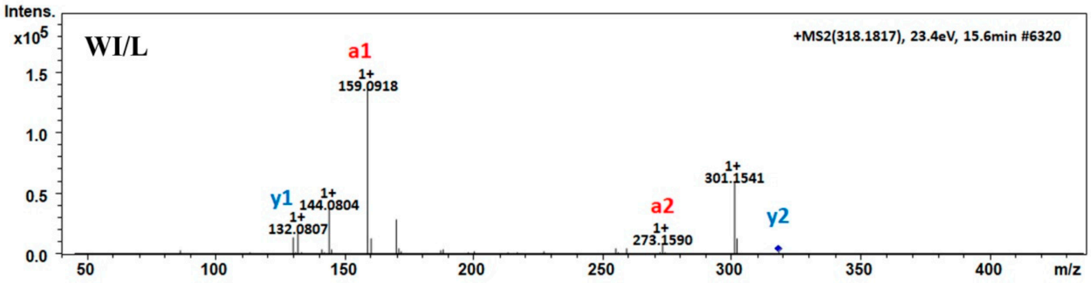

Supplementary Figure 65. Mass spectra and de novo sequencing analysis of WI/L

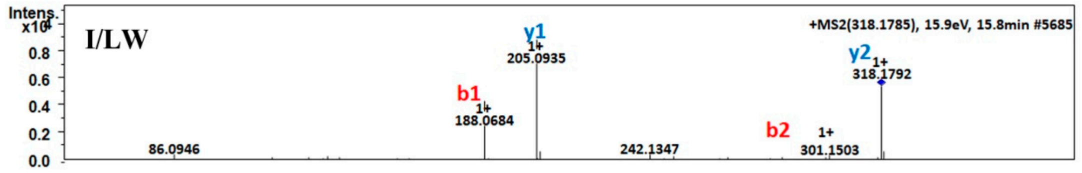

Supplementary Figure 66. Mass spectra and de novo sequencing analysis of I/LW

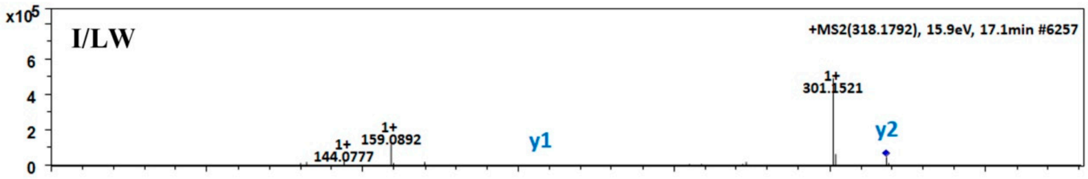

Supplementary Figure 67. Mass spectra and de novo sequencing analysis of I/LW

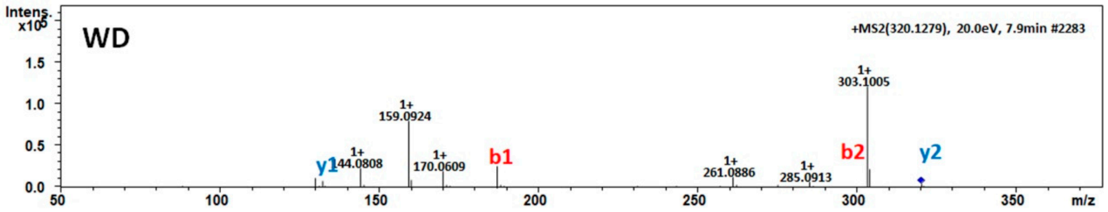

Supplementary Figure 68. Mass spectra and de novo sequencing analysis of WD

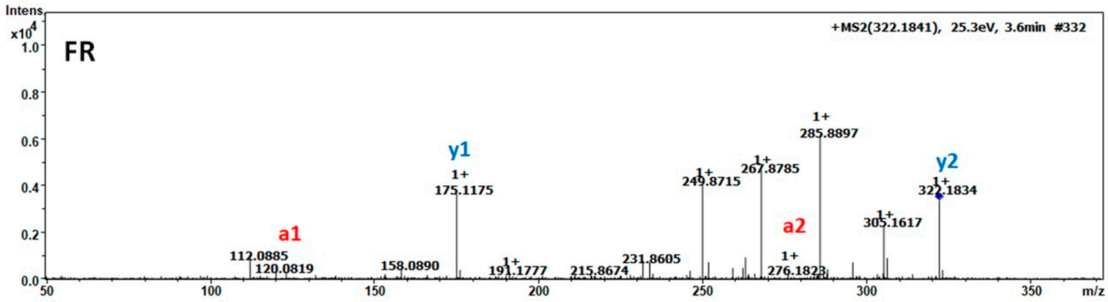

Supplementary Figure 69. Mass spectra and de novo sequencing analysis of

FR

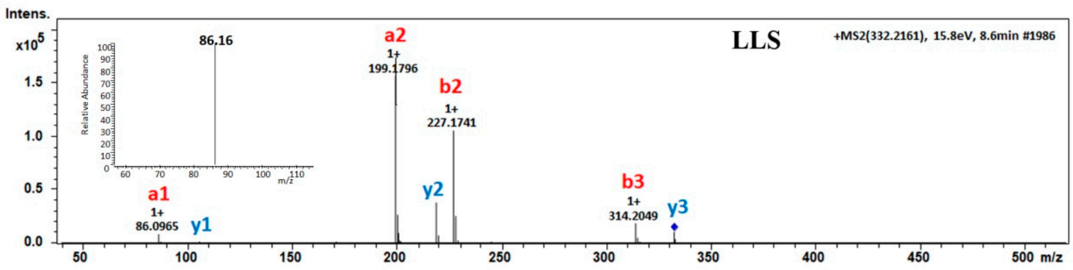

Supplementary Figure 70. Mass spectra and de novo sequencing analysis of LLS

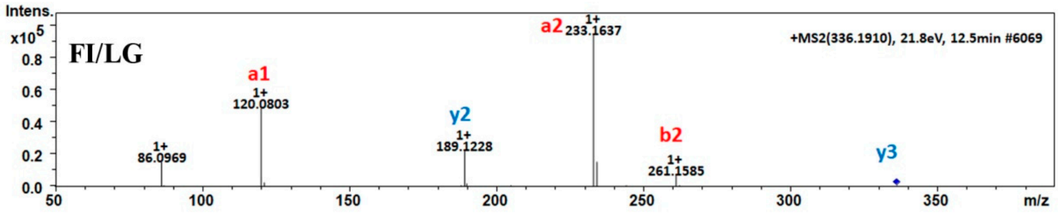

Supplementary Figure 71. Mass spectra and de novo sequencing analysis of FI/LG

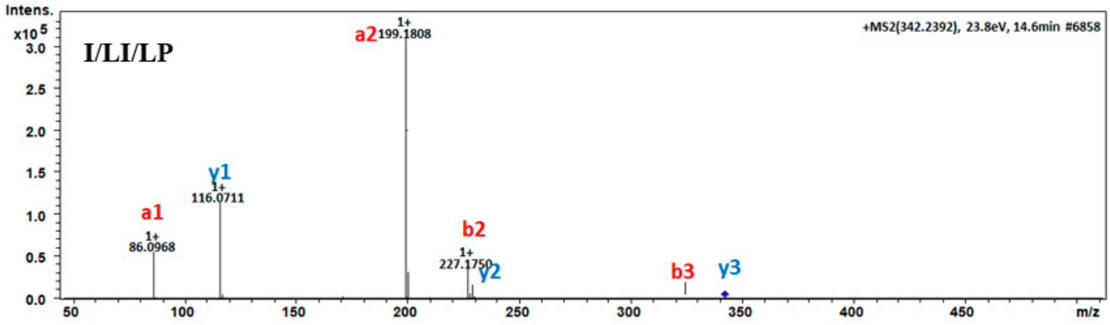

Supplementary Figure 72. Mass spectra and de novo sequencing analysis of I/LI/LP

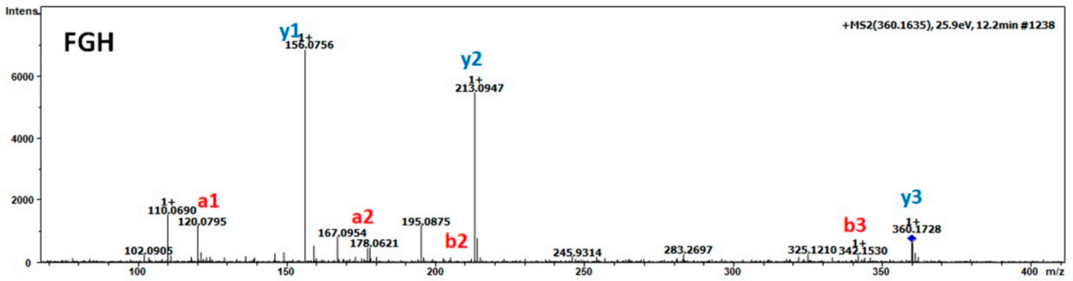

Supplementary Figure73. Mass spectra and de novo sequencing analysis of FGH

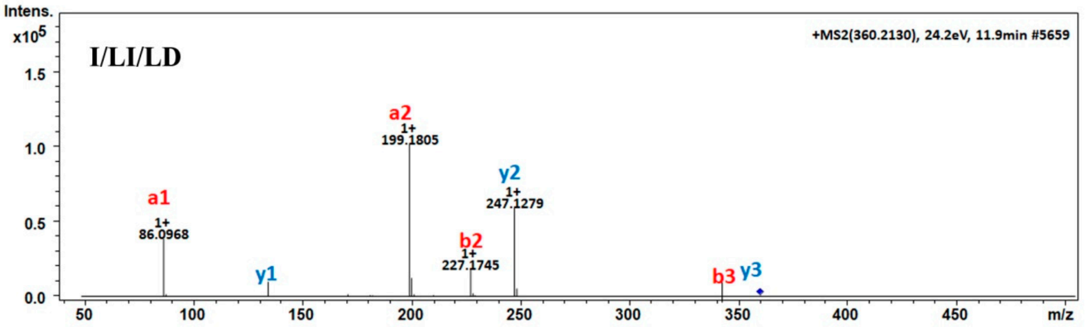

Supplementary Figure74. Mass spectra and de novo sequencing analysis of I/LI/LD

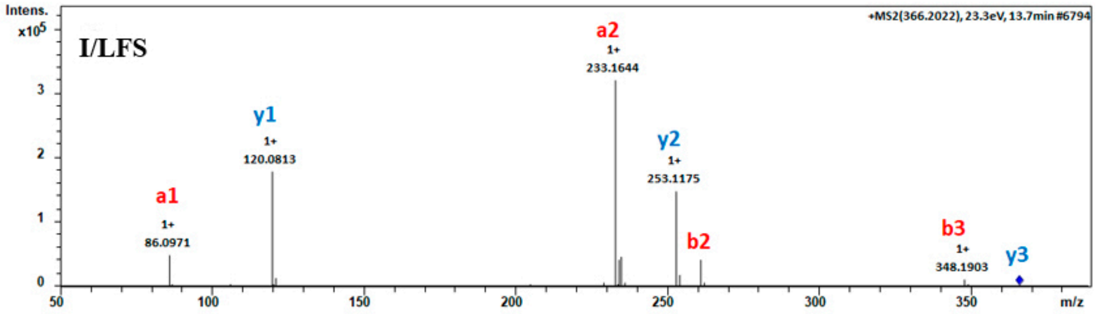

Supplementary Figure75. Mass spectra and de novo sequencing analysis of I/LFS

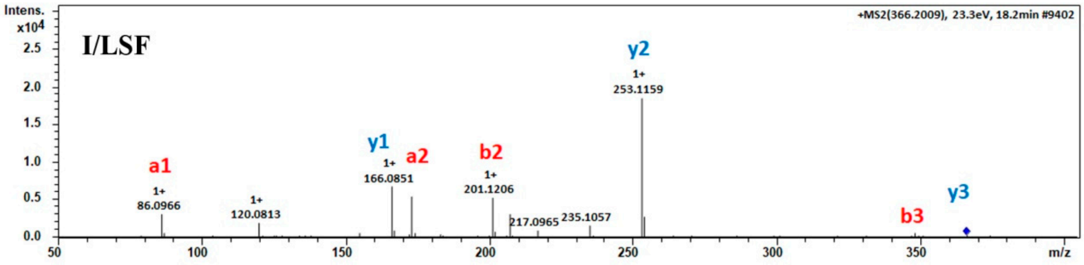

Supplementary Figure76. Mass spectra and de novo sequencing analysis of I/LSF

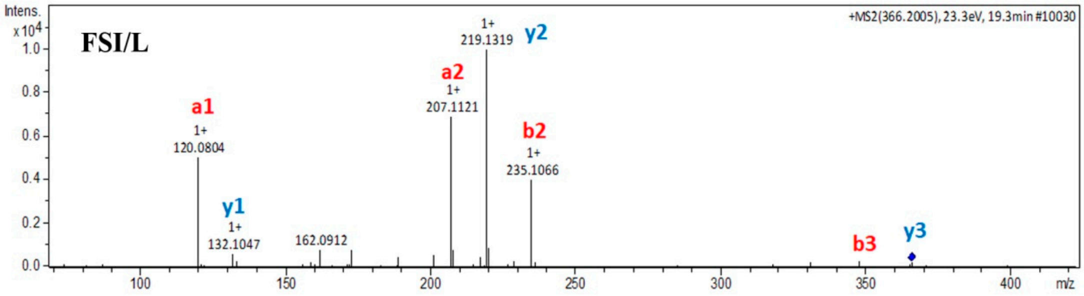

Supplementary Figure77. Mass spectra and de novo sequencing analysis of FSI/L

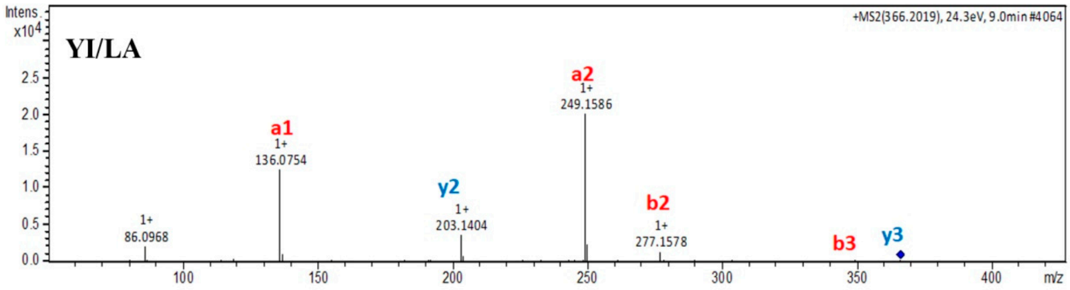

Supplementary Figure78. Mass spectra and de novo sequencing analysis of YI/LA

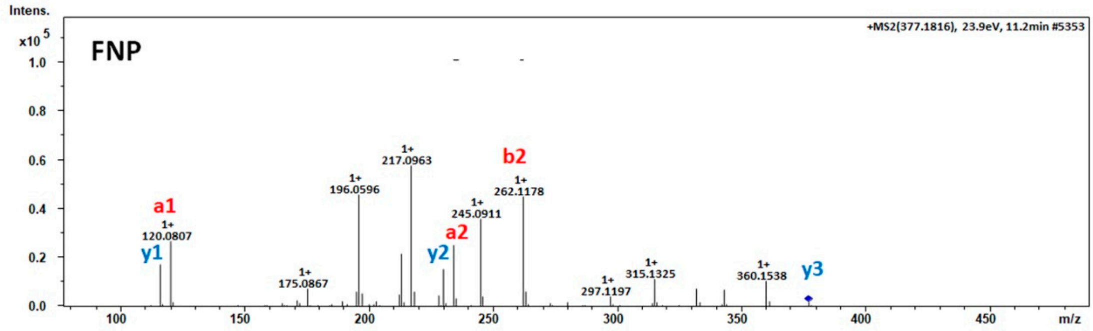

Supplementary Figure 79. Mass spectra and de novo sequencing analysis of FNP

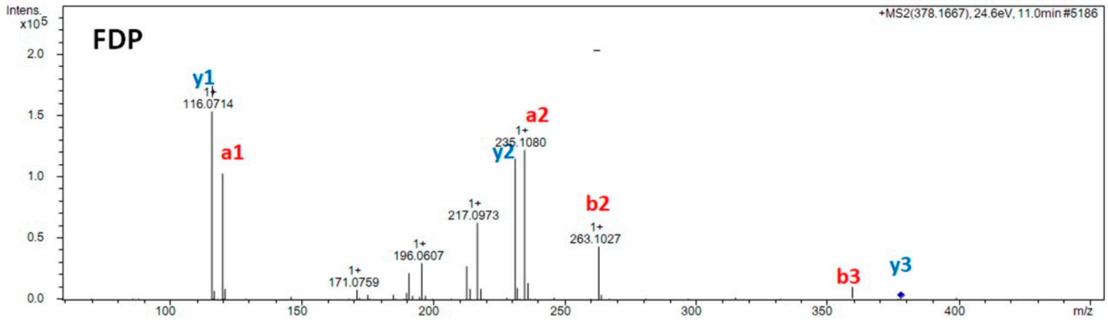

Supplementary Figure 80. Mass spectra and de novo sequencing analysis of FDP

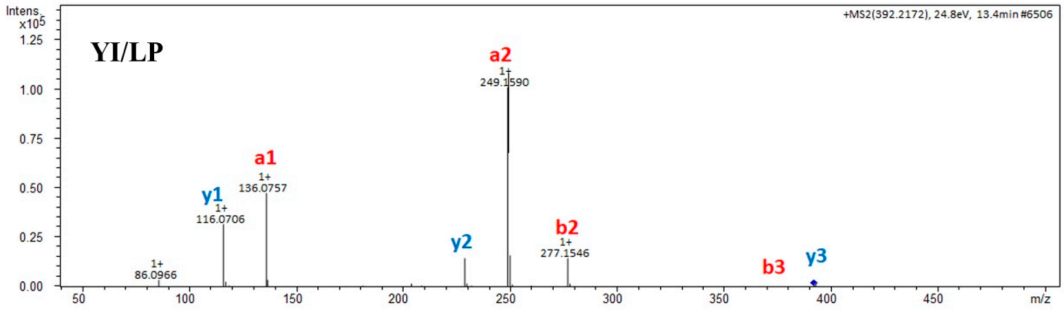

Supplementary Figure 81. Mass spectra and de novo sequencing analysis of YI/LP

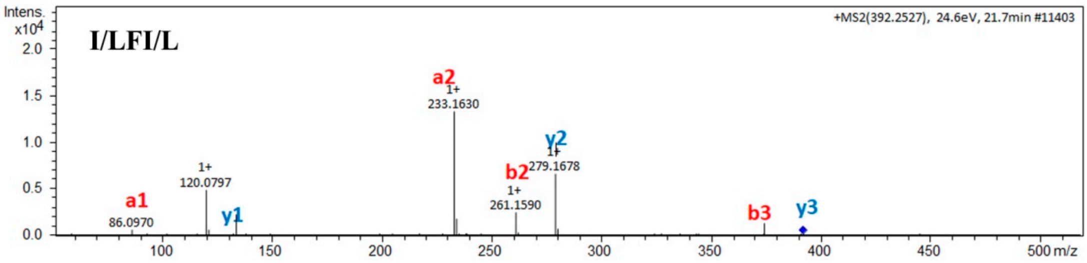

Supplementary Figure 82. Mass spectra and de novo sequencing analysis of I/LFI/L

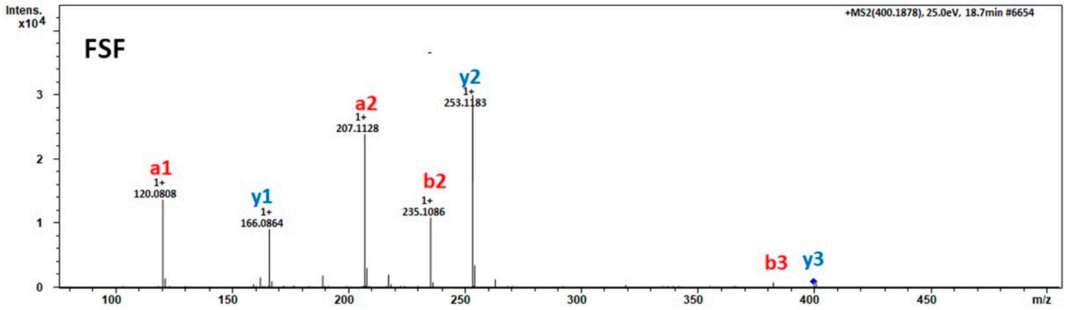

Supplementary Figure 83. Mass spectra and de novo sequencing analysis of FSF

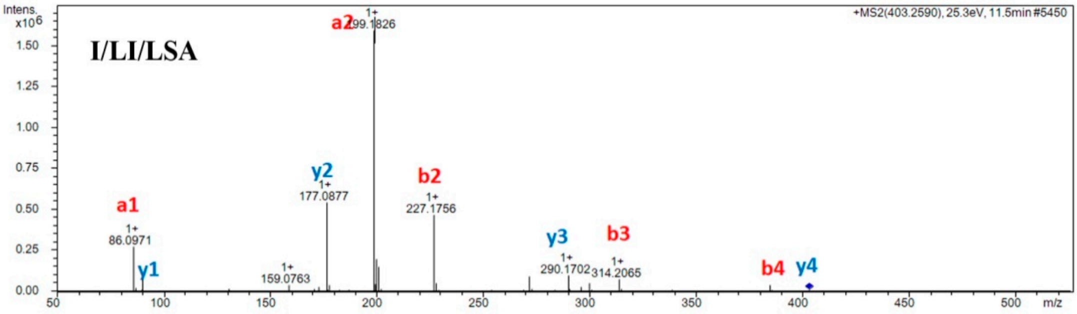

Supplementary Figure 84. Mass spectra and de novo sequencing analysis of I/LI/LSA

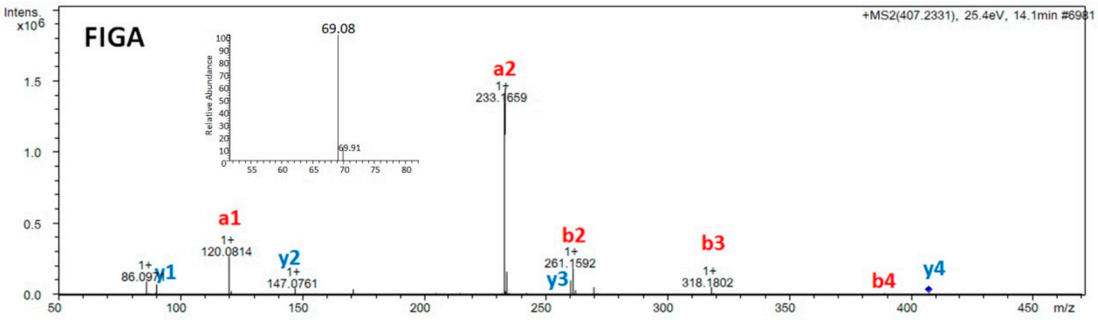

Supplementary Figure 85. Mass spectra and de novo sequencing analysis of FIGA

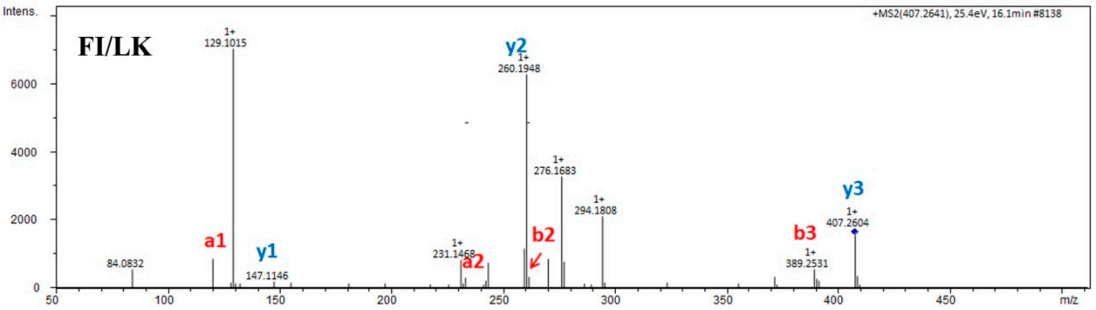

Supplementary Figure73. Mass spectra and de novo sequencing analysis of FI/LK

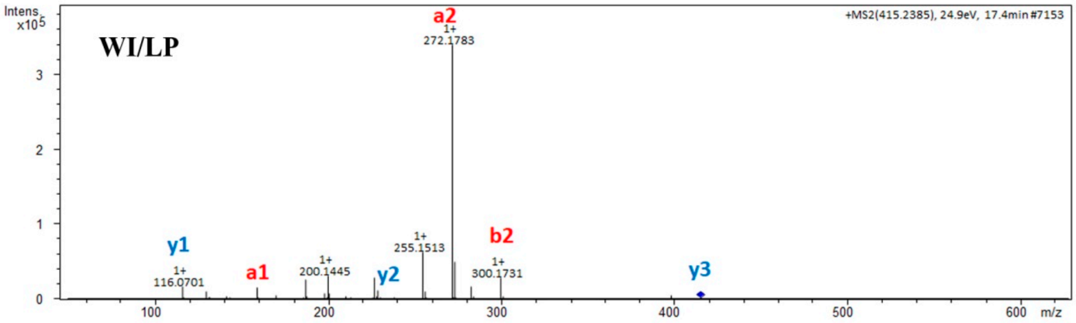

Supplementary Figure 87. Mass spectra and de novo sequencing analysis of WI/LP

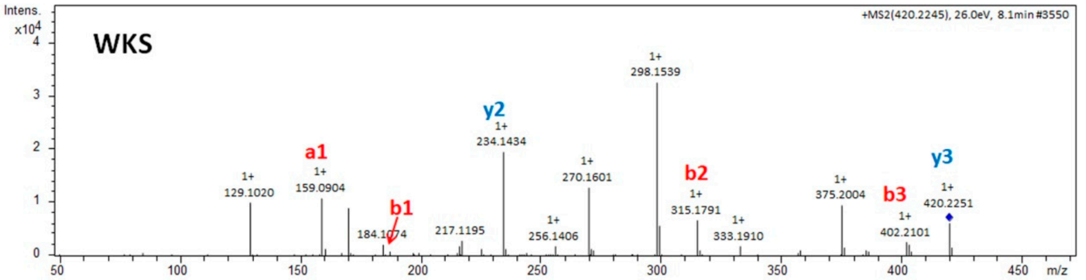

Supplementary Figure 88. Mass spectra and de novo sequencing analysis of WKS

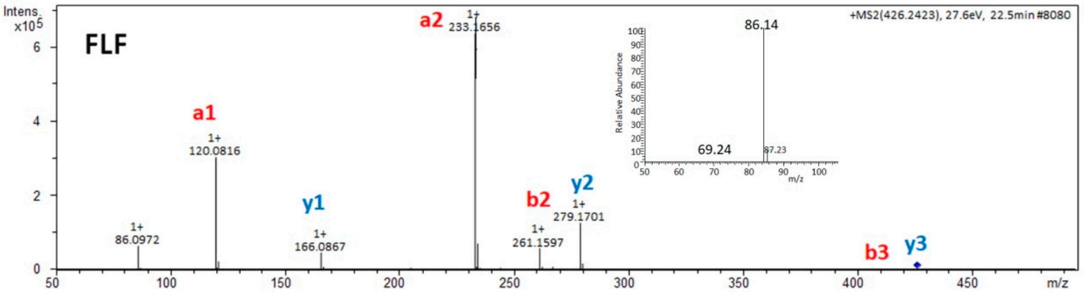

Supplementary Figure 89. Mass spectra and de novo sequencing analysis of FLF

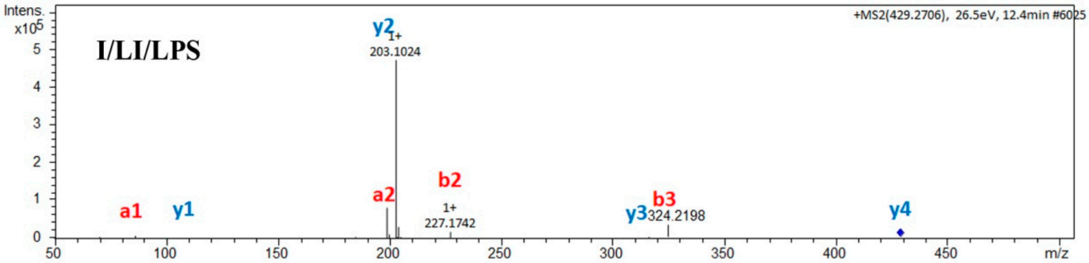

Supplementary Figure 90. Mass spectra and de novo sequencing analysis of I/LI/LPS

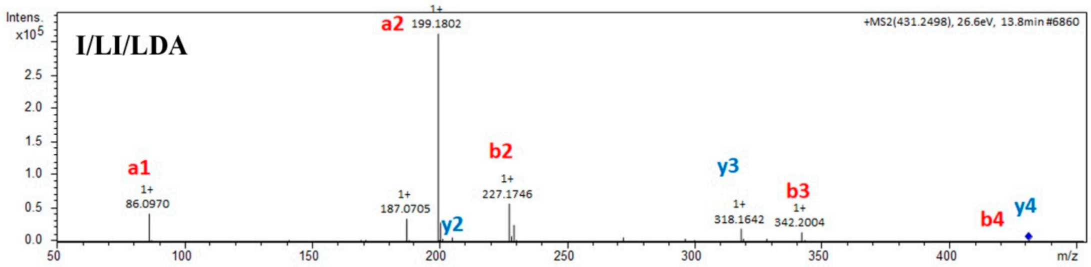

Supplementary Figure 91. Mass spectra and de novo sequencing analysis of I/LI/LDA

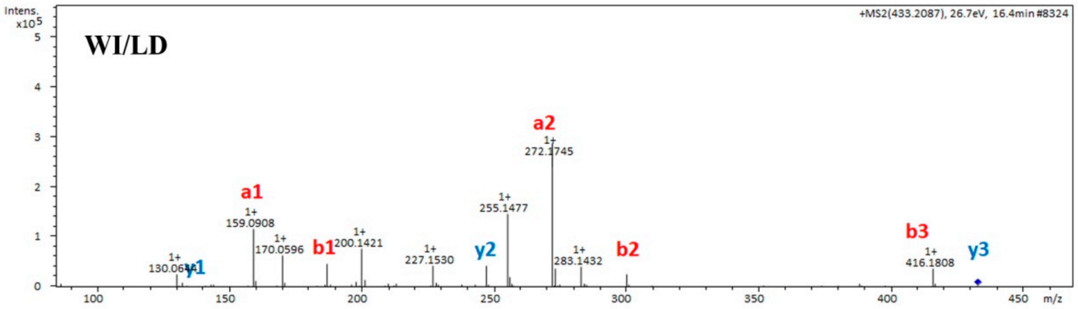

Supplementary Figure 92. Mass spectra and de novo sequencing analysis of WI/LD

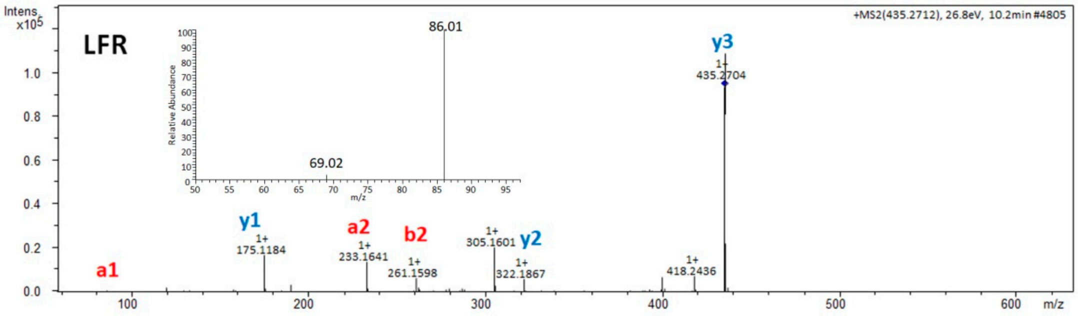

Supplementary Figure 93. Mass spectra and de novo sequencing analysis of LFR

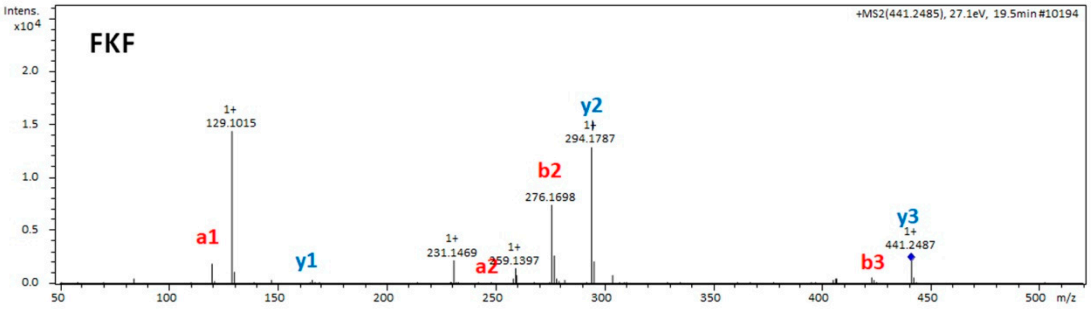

Supplementary Figure 94. Mass spectra and de novo sequencing analysis of FKF

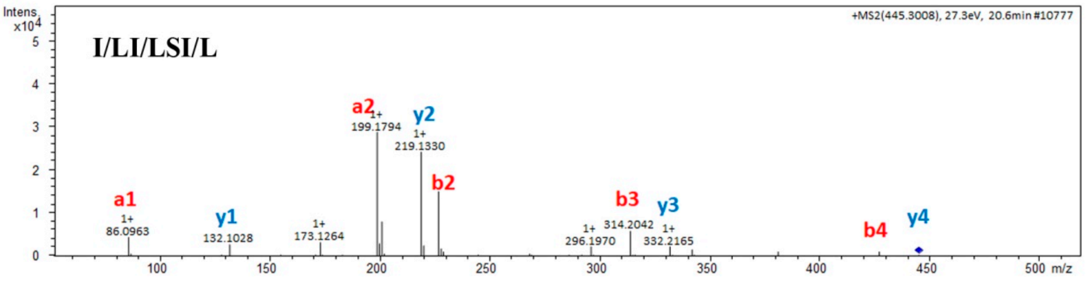

Supplementary Figure 95. Mass spectra and de novo sequencing analysis of I/LI/LSI/L

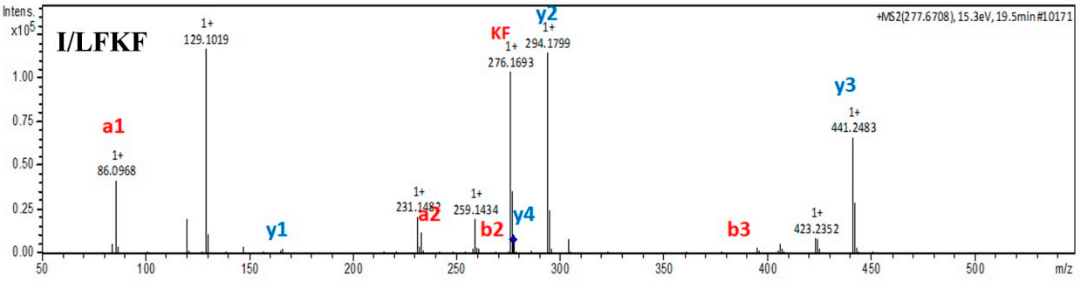

Supplementary Figure 96. Mass spectra and de novo sequencing analysis of I/LFKF

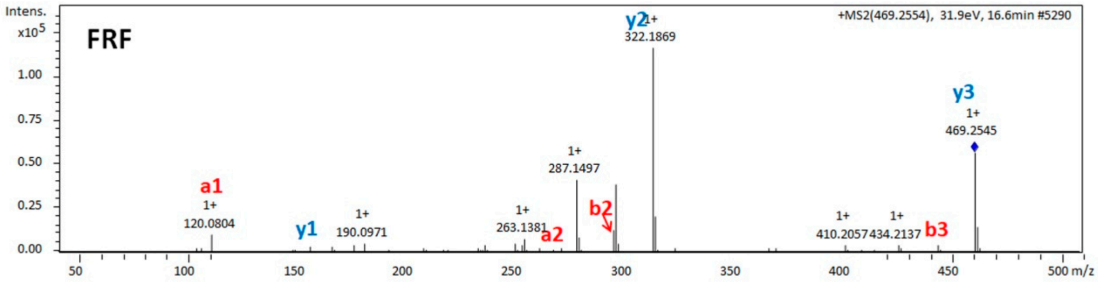

Supplementary Figure 97. Mass spectra and de novo sequencing analysis of FRF

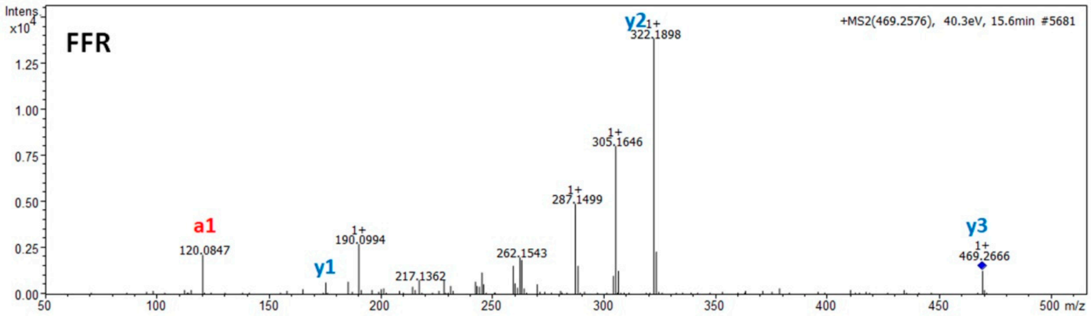

Supplementary Figure 98. Mass spectra and de novo sequencing analysis of FFR

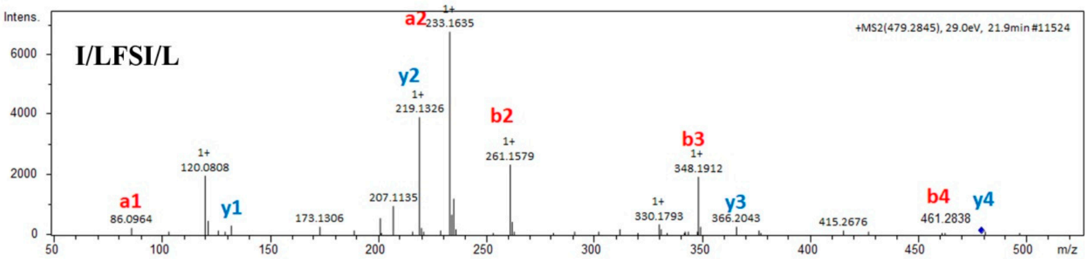

Supplementary Figure 99. Mass spectra and de novo sequencing analysis of I/LFSI/L

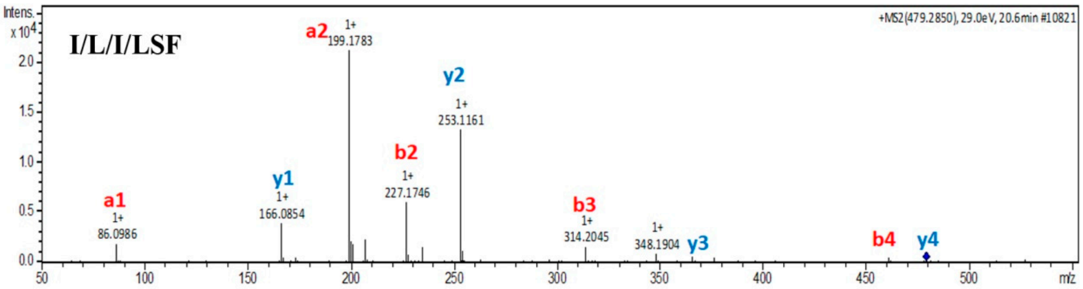

Supplementary Figure 100. Mass spectra and de novo sequencing analysis of I/LI/LSF

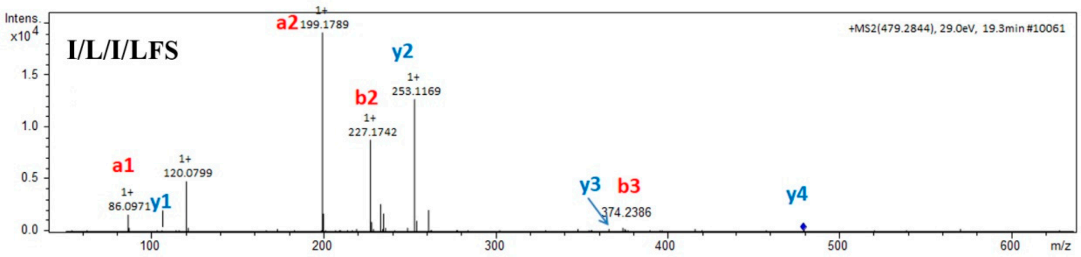

Supplementary Figure 101. Mass spectra and de novo sequencing analysis of I/LI/LFS

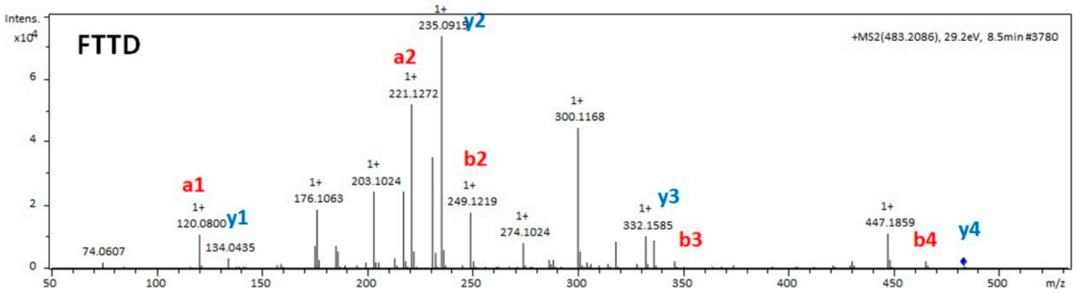

Supplementary Figure 102. Mass spectra and de novo sequencing analysis of FTTD

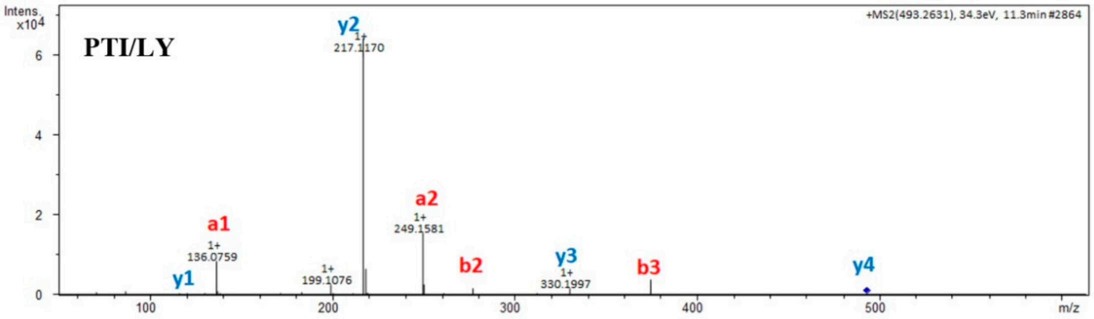

Supplementary Figure 103. Mass spectra and de novo sequencing analysis of PTI/LY

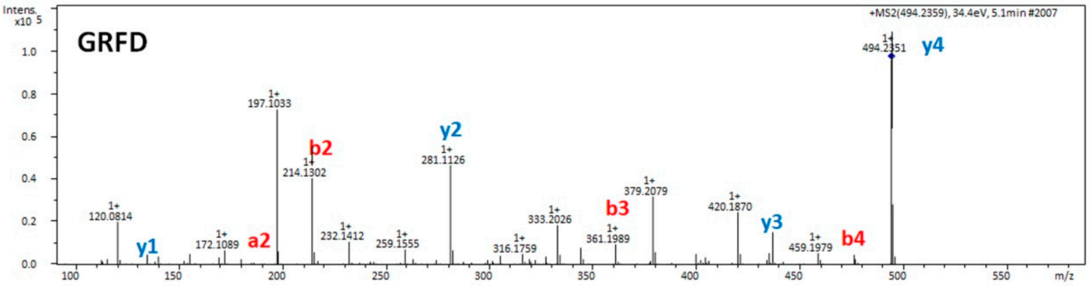

Supplementary Figure 104. Mass spectra and de novo sequencing analysis of GRFD

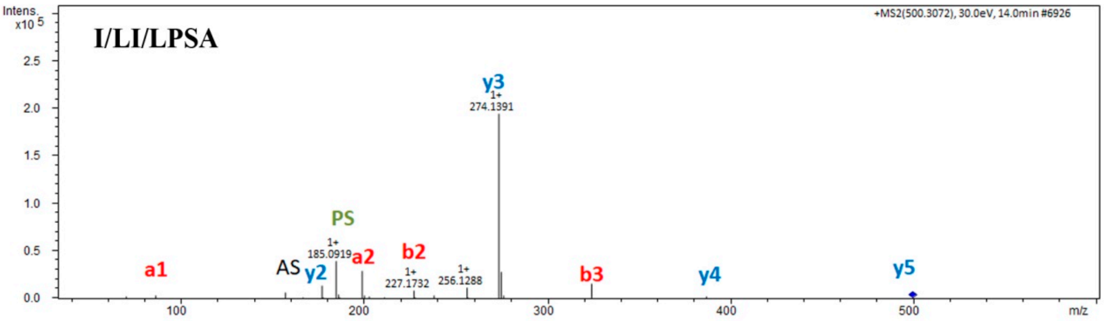

Supplementary Figure 105. Mass spectra and de novo sequencing analysis of I/LI/LPSA

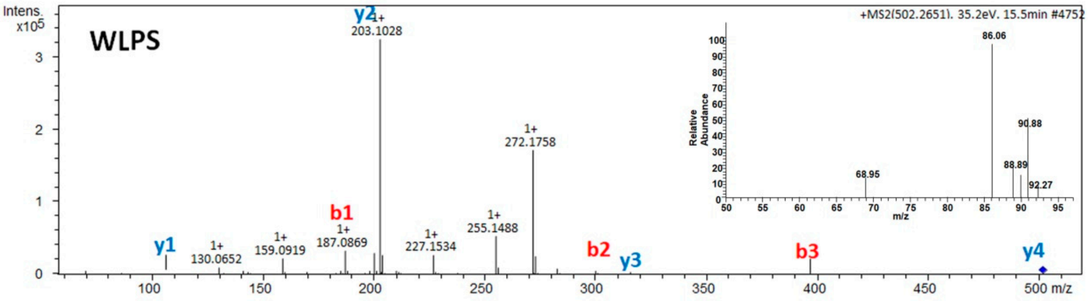

Supplementary Figure 106. Mass spectra and de novo sequencing analysis of WLPS

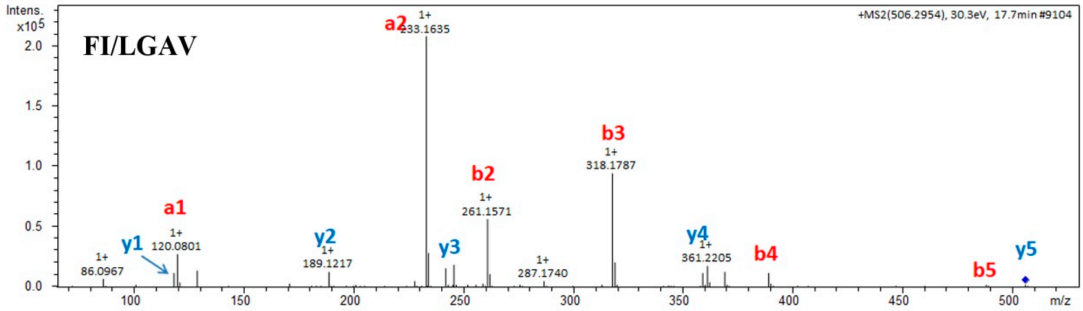

Supplementary Figure 107. Mass spectra and de novo sequencing analysis of FI/LGAV

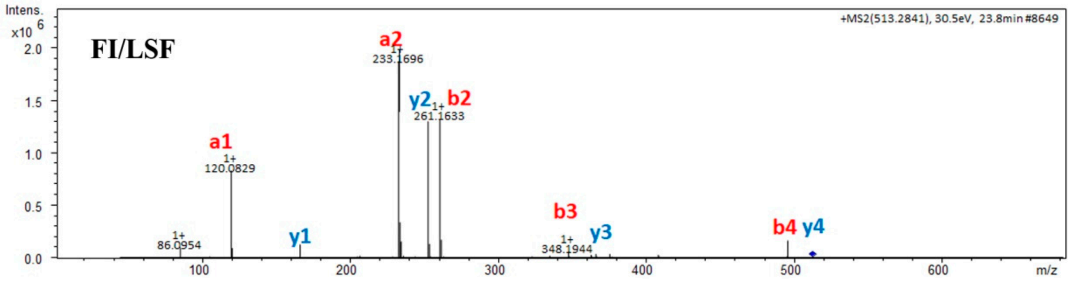

Supplementary Figure 108. Mass spectra and de novo sequencing analysis of FI/LSF

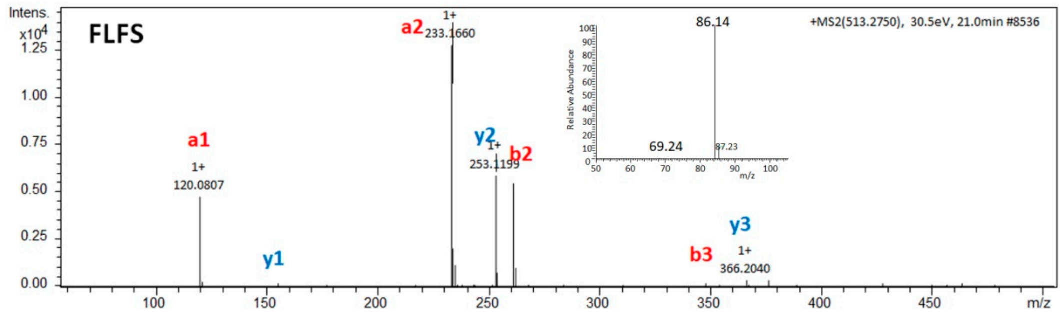

Supplementary Figure 109. Mass spectra and de novo sequencing analysis of FLFS

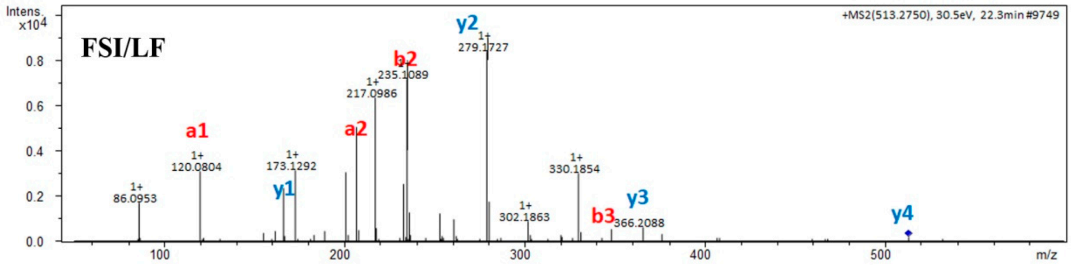

Supplementary Figure 110. Mass spectra and de novo sequencing analysis of FSI/LF

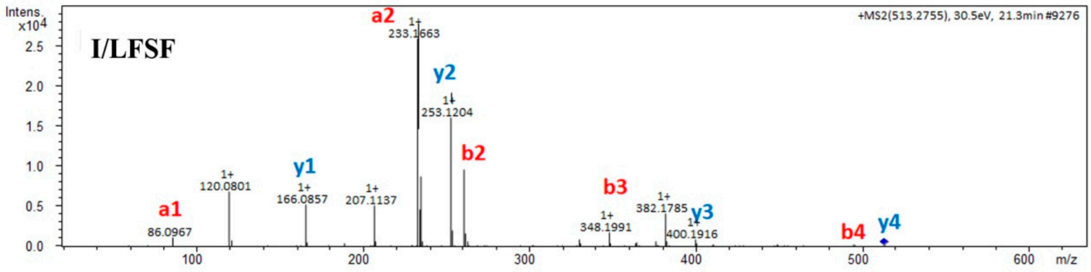

Supplementary Figure 111. Mass spectra and de novo sequencing analysis of I/LFSF

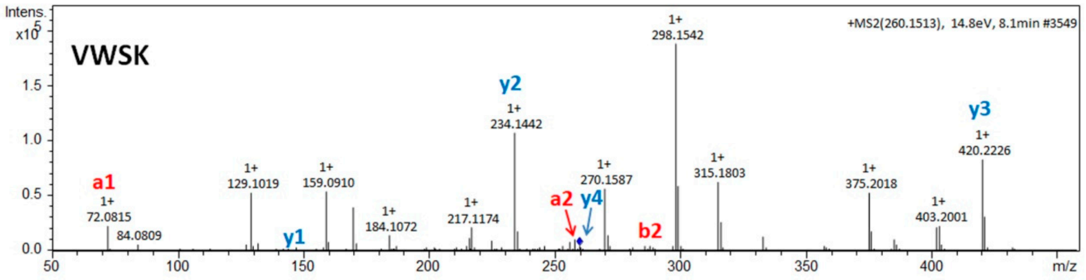

Supplementary Figure 112. Mass spectra and de novo sequencing analysis of VWSK

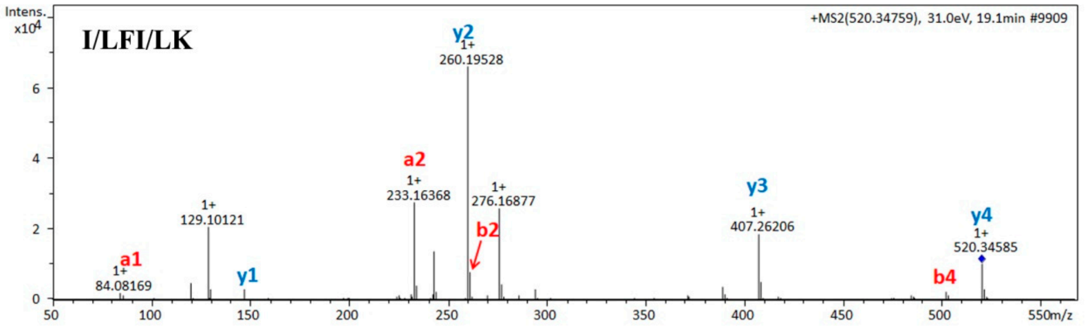

Supplementary Figure 113. Mass spectra and de novo sequencing analysis of I/LFI/LK

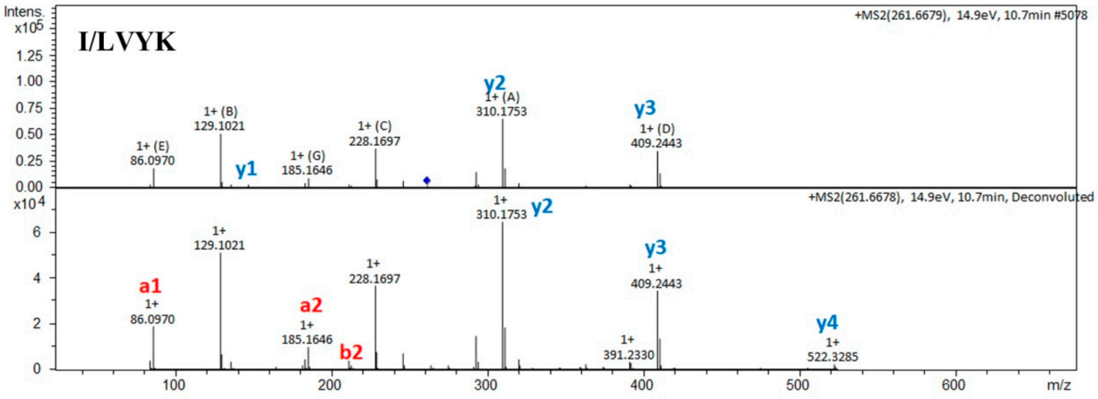

Supplementary Figure 114. Mass spectra and de novo sequencing analysis of I/LVYK

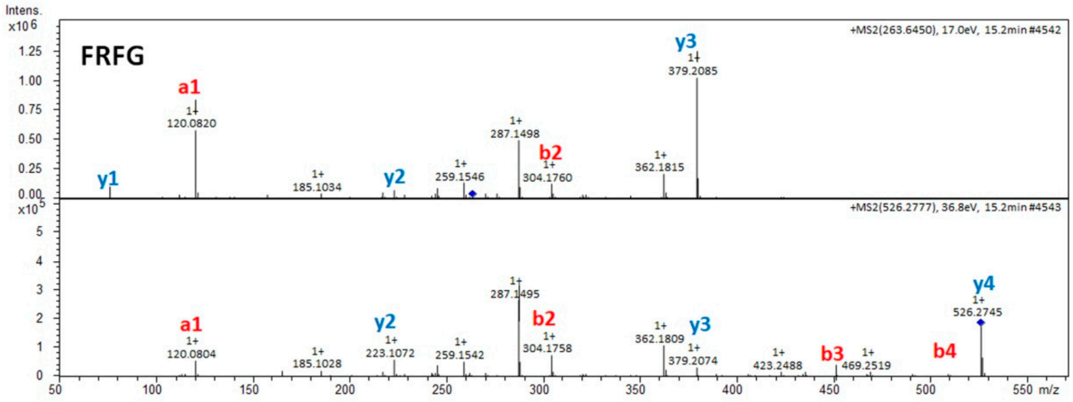

Supplementary Figure 115. Mass spectra and de novo sequencing analysis of FRFG

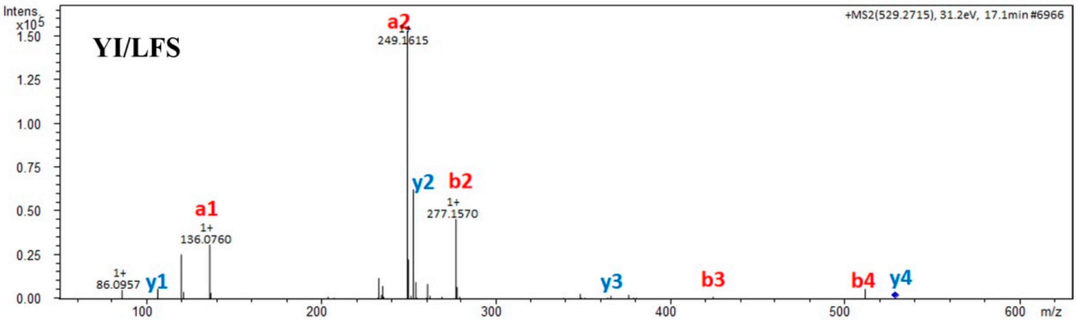

Supplementary Figure 116. Mass spectra and de novo sequencing analysis of YI/LFS

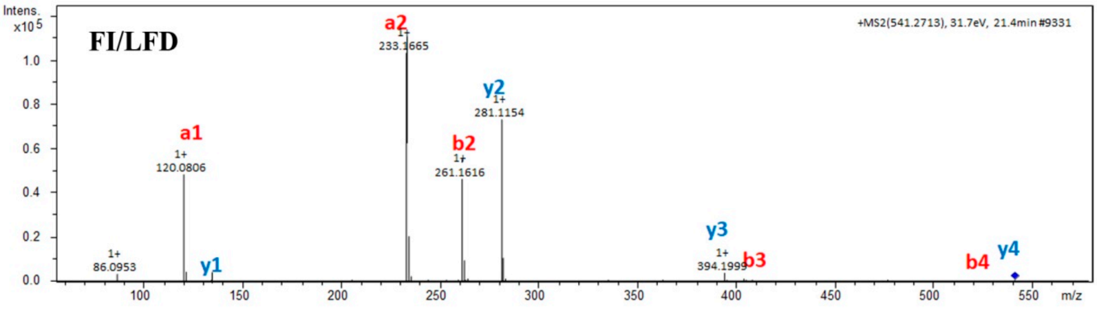

Supplementary Figure 117. Mass spectra and de novo sequencing analysis of FI/LFD

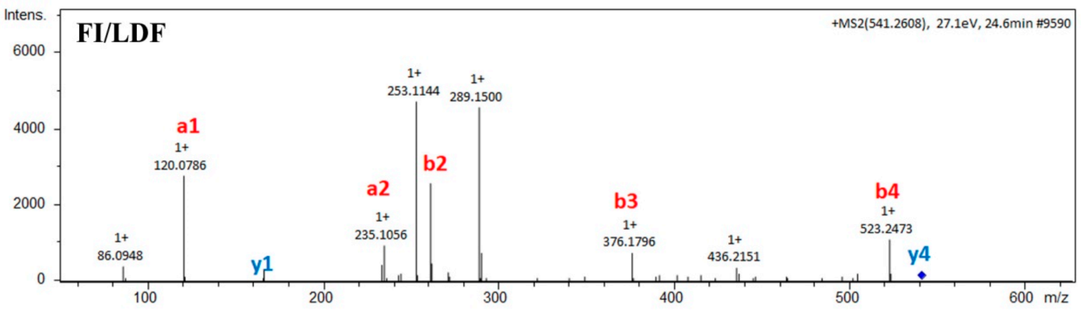

Supplementary Figure 118. Mass spectra and de novo sequencing analysis of FI/LDF

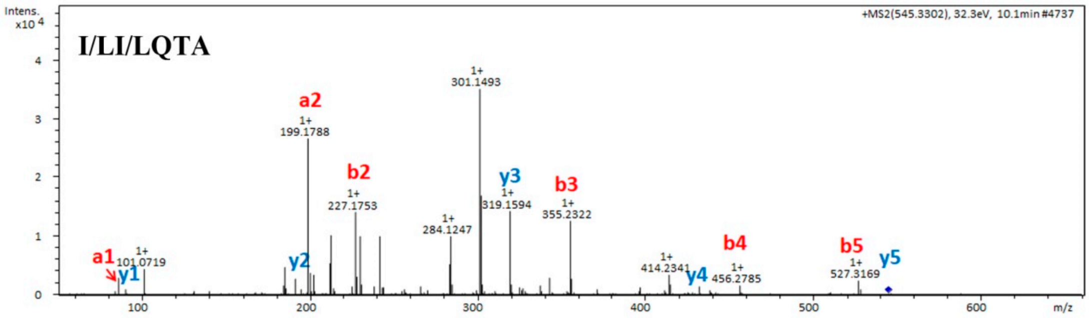

Supplementary Figure 119. Mass spectra and de novo sequencing analysis of I/LI/LQTA

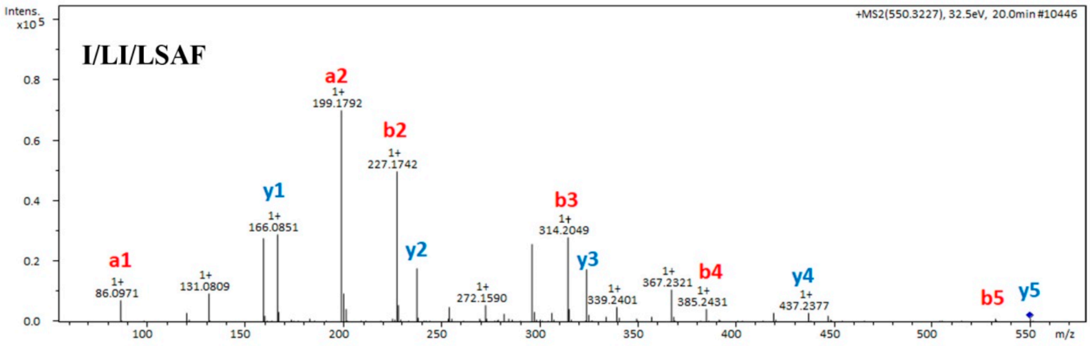

Supplementary Figure 120. Mass spectra and de novo sequencing analysis of I/LI/LSAF

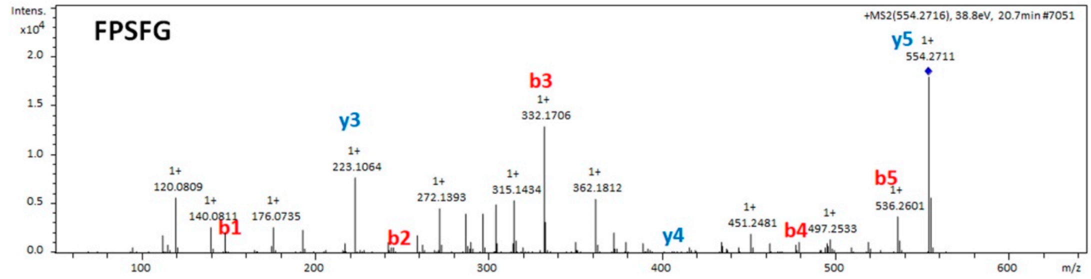

Supplementary Figure 121. Mass spectra and de novo sequencing analysis of FPSFG

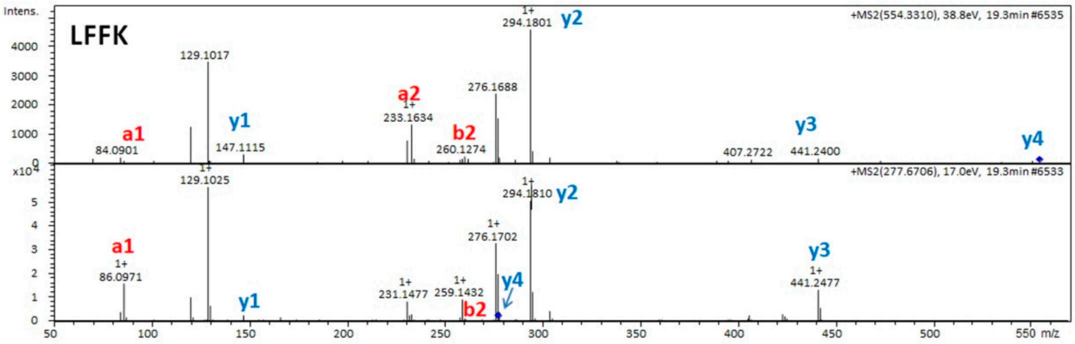

Supplementary Figure 122. Mass spectra and de novo sequencing analysis of LFFK

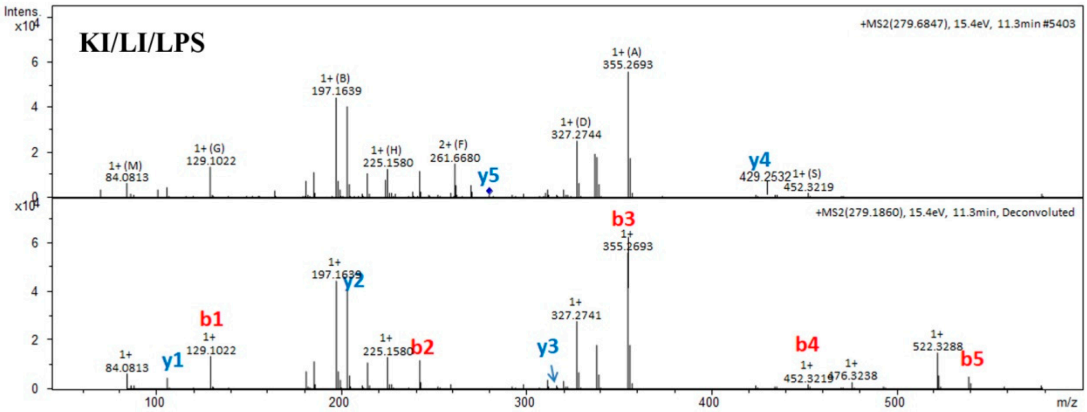

Supplementary Figure 123. Mass spectra and de novo sequencing analysis of KI/LI/LPS

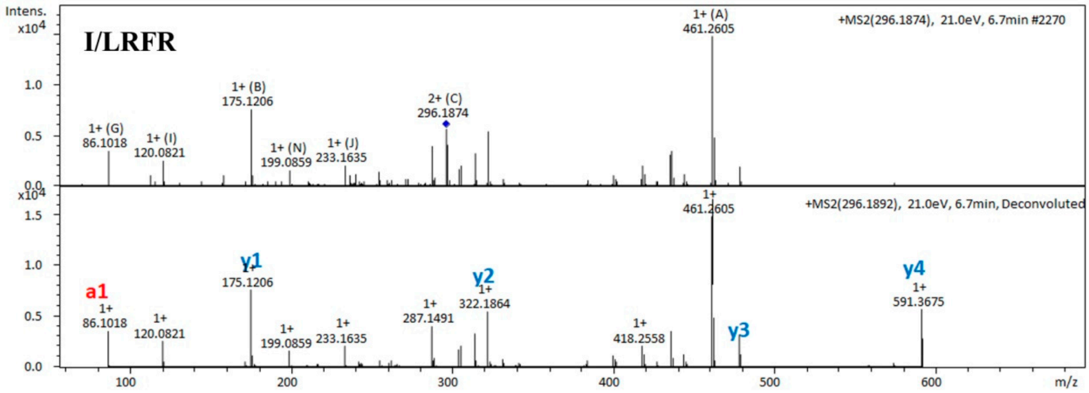

Supplementary Figure 124. Mass spectra and de novo sequencing analysis of I/LRFR

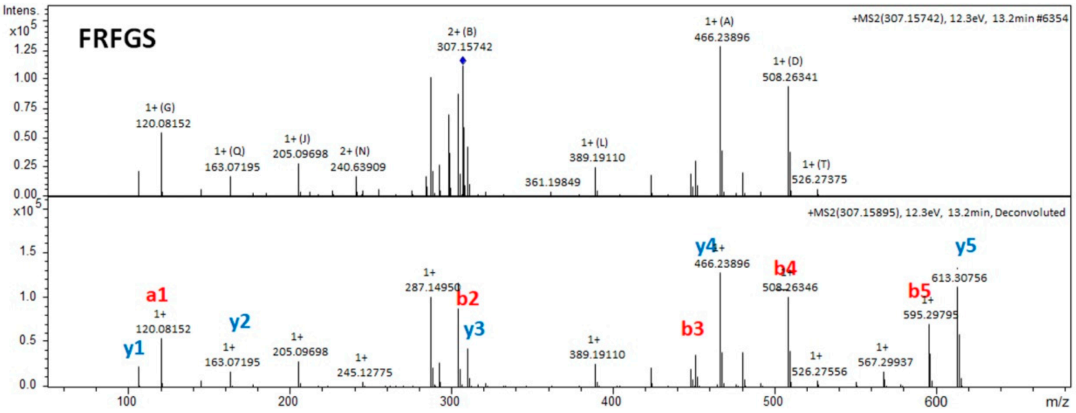

Supplementary Figure 125. Mass spectra and de novo sequencing analysis of FRFGS

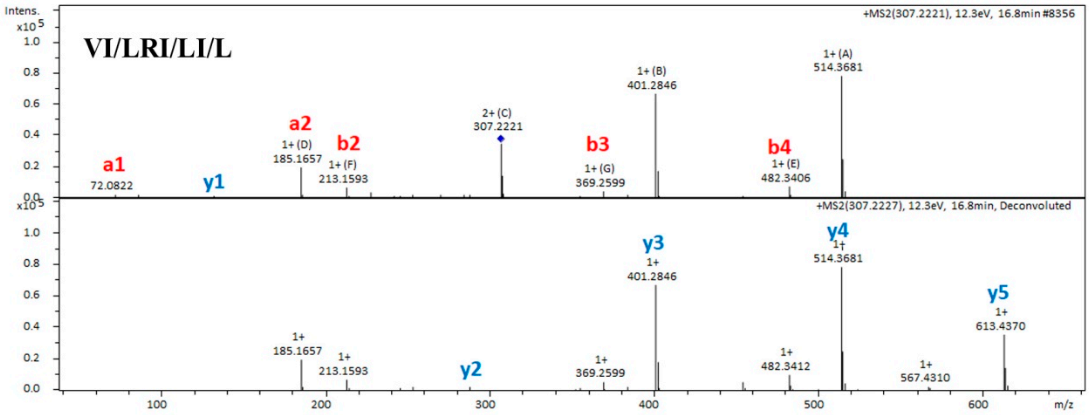

Supplementary Figure 126. Mass spectra and de novo sequencing analysis of

VI/LRI/LI/L

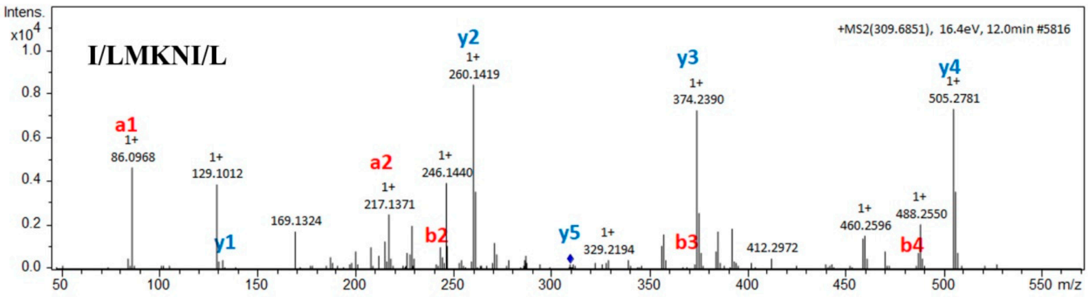

Supplementary Figure 127. Mass spectra and de novo sequencing analysis of I/LMKNI/L

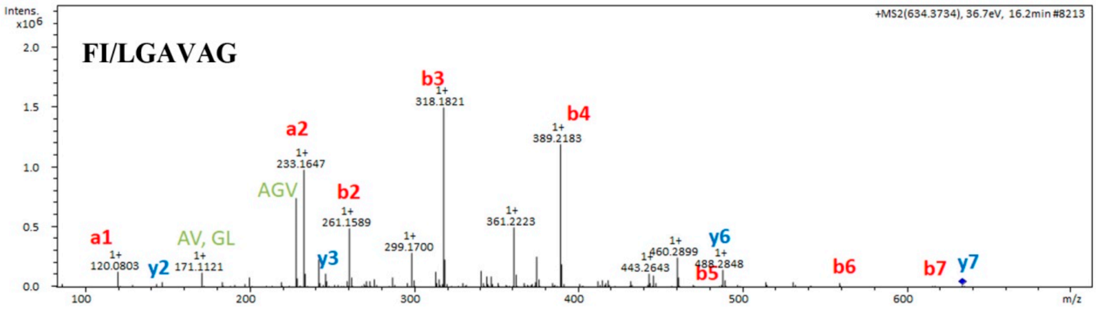

Supplementary Figure 128. Mass spectra and de novo sequencing analysis of FI/LGAVAG

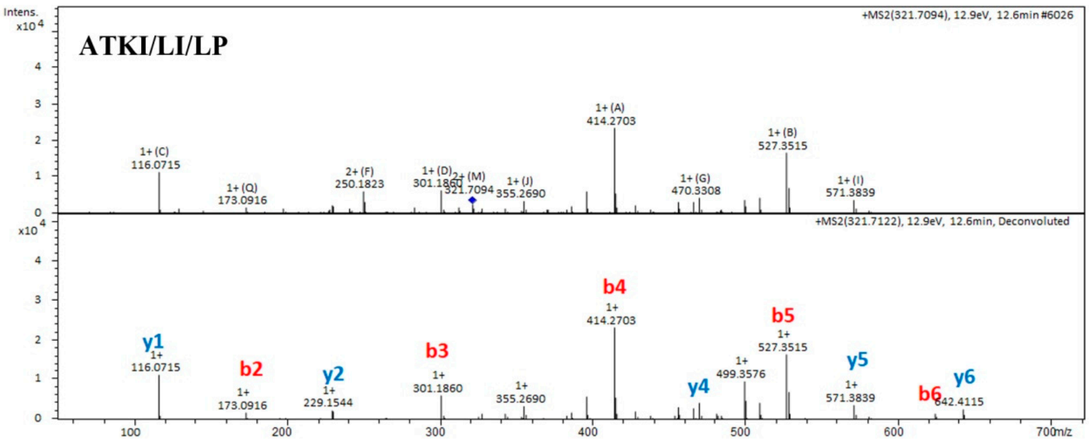

Supplementary Figure 129. Mass spectra and de novo sequencing analysis of ATKI/LI/LP

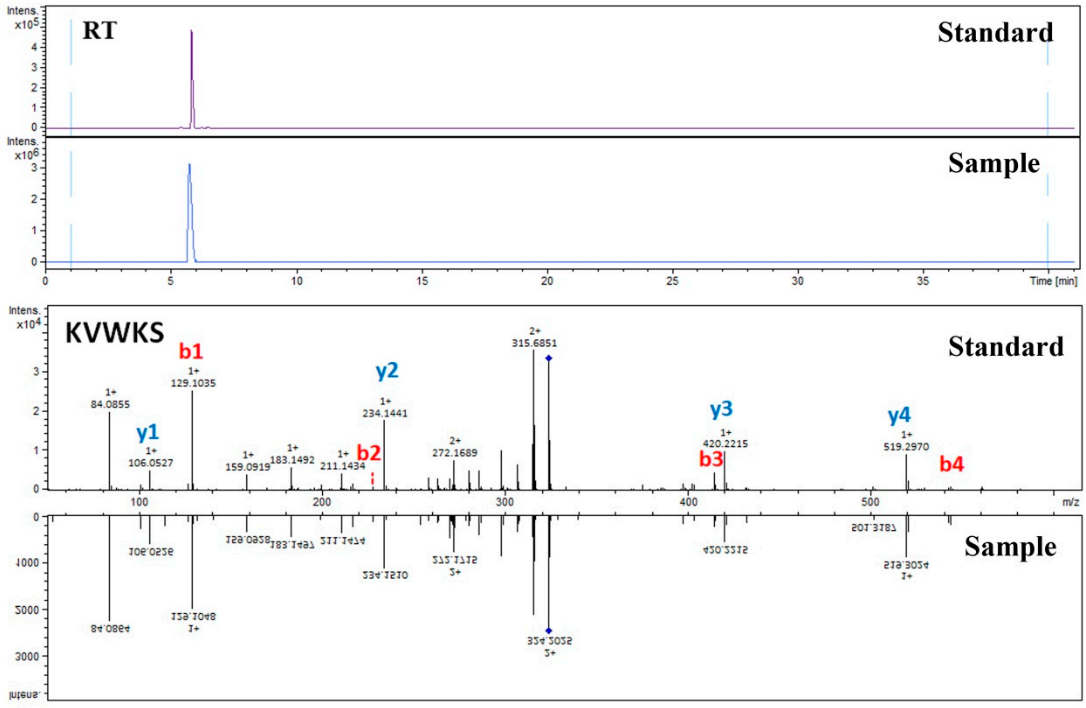

Supplementary Figure 130. Mass spectra and de novo sequencing analysis of KVWKS

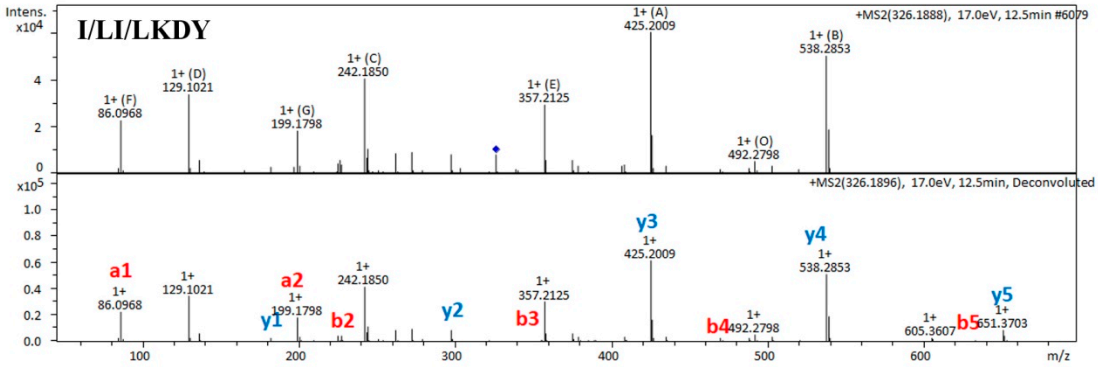

Supplementary Figure 131. Mass spectra and de novo sequencing analysis of I/LI/LKDY

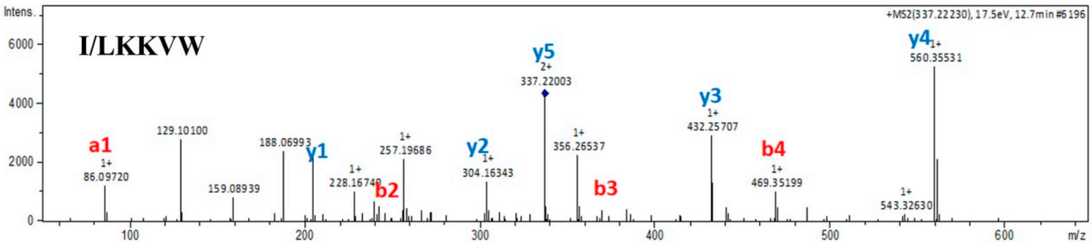

Supplementary Figure 132. Mass spectra and de novo sequencing analysis of I/LKKVW

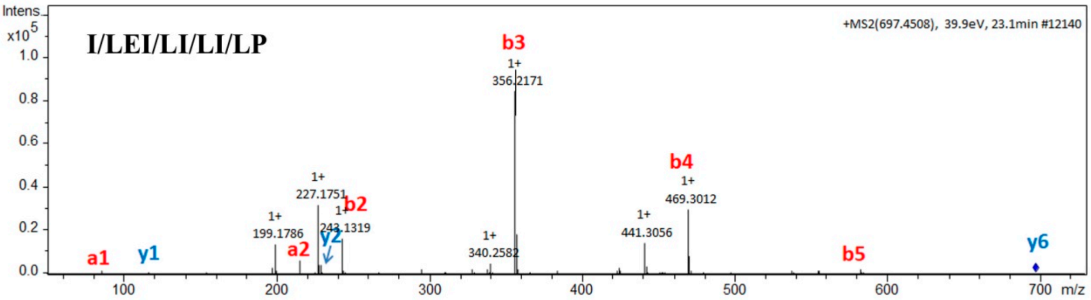

Supplementary Figure 133. Mass spectra and de novo sequencing analysis of I/LEI/LI/LI/LP

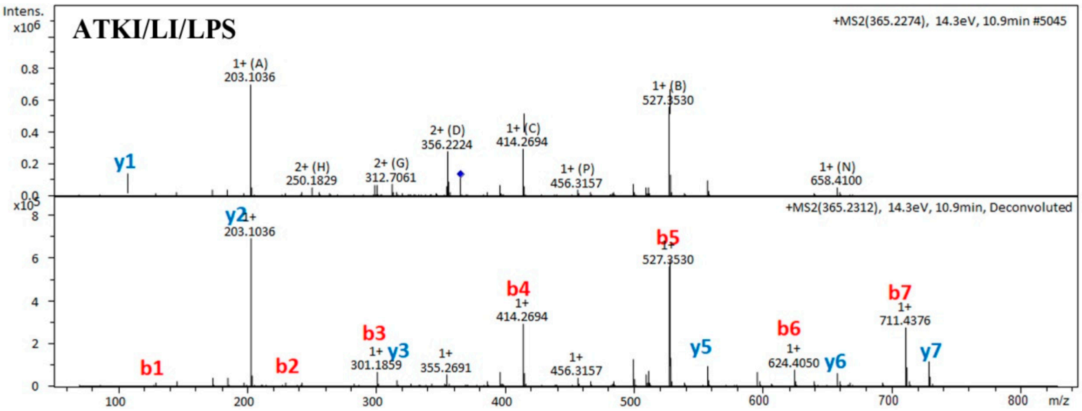

Supplementary Figure 134. Mass spectra and de novo sequencing analysis of ATK I/LI/LPS

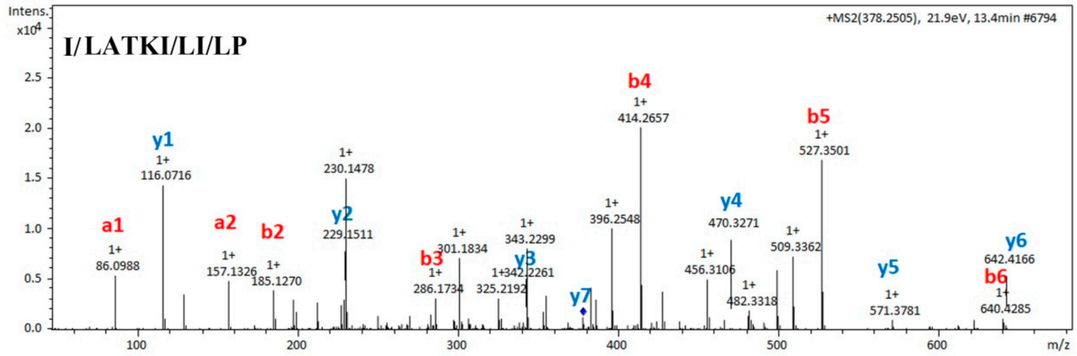

Supplementary Figure 135. Mass spectra and de novo sequencing analysis of

I/LATK I/LI/LP

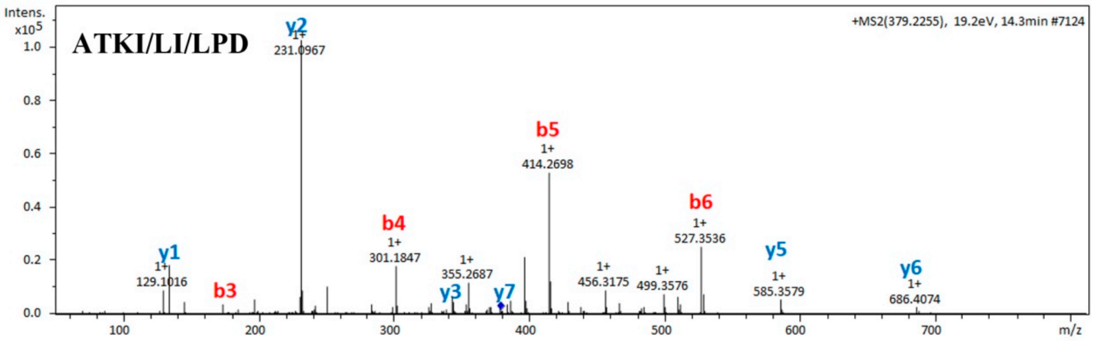

Supplementary Figure 136. Mass spectra and de novo sequencing analysis of

ATK I/LI/LPD

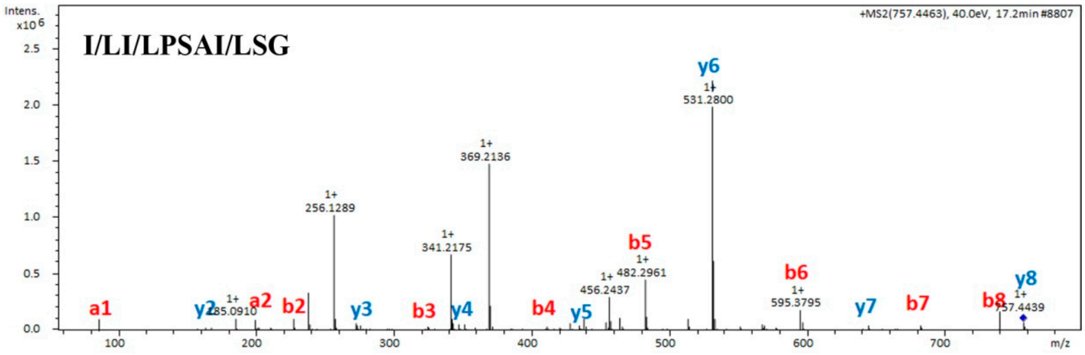

Supplementary Figure 137. Mass spectra and de novo sequencing analysis of

I/LI/LPSAI/LSG

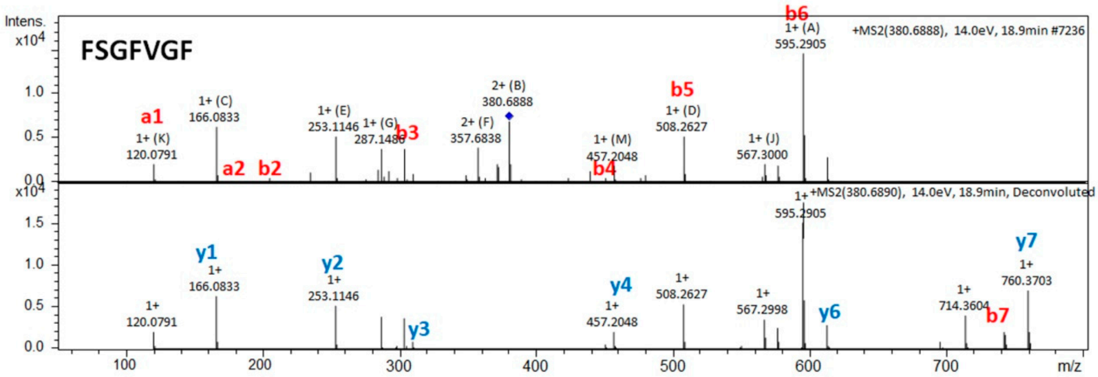

Supplementary Figure 138. Mass spectra and de novo sequencing analysis of FSGFVGF

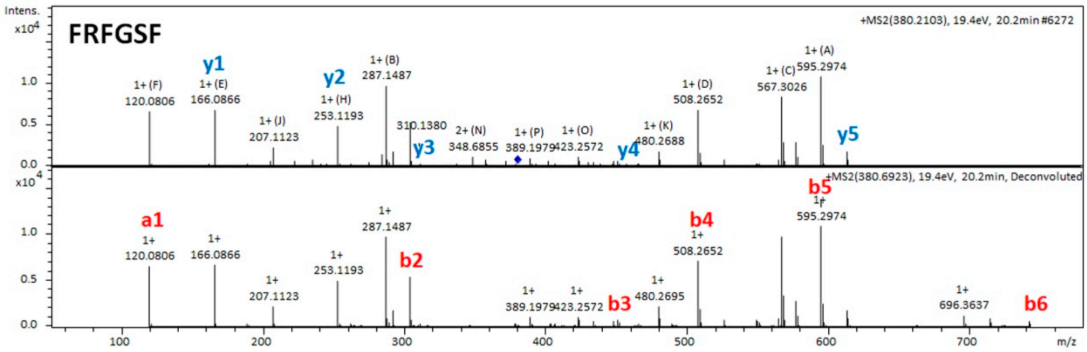

Supplementary Figure 139. Mass spectra and de novo sequencing analysis of FRFGSF

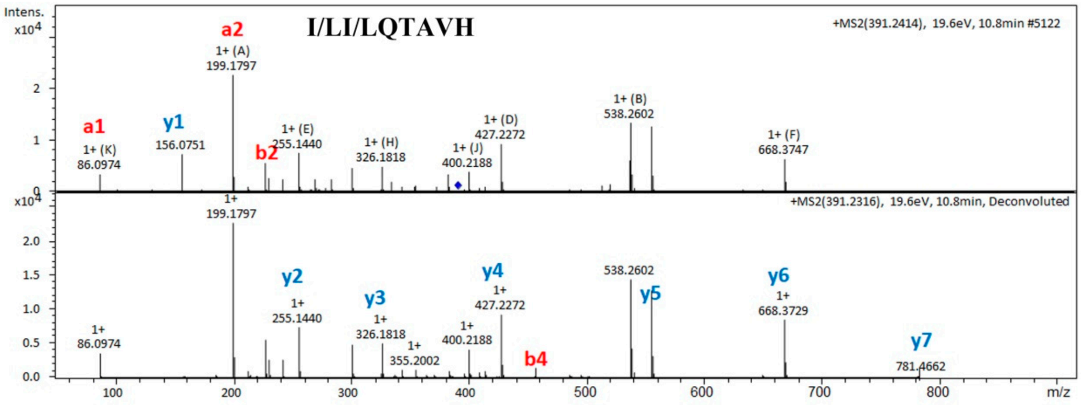

Supplementary Figure 140. Mass spectra and de novo sequencing analysis of I/LI/LQTAVH

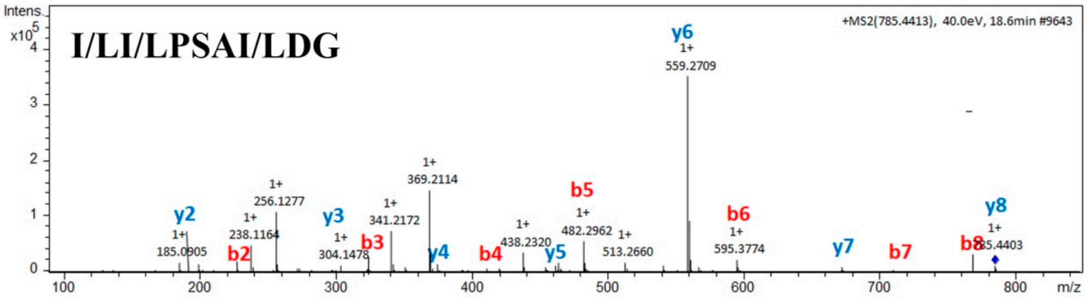

Supplementary Figure 141. Mass spectra and de novo sequencing analysis of

I/LI/LPSAI/LDG

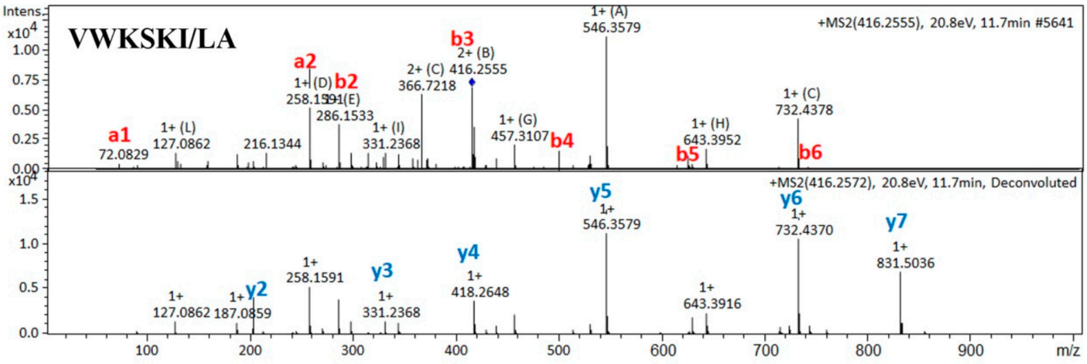

Supplementary Figure 142. Mass spectra and de novo sequencing analysis of

VWKSKI/LA

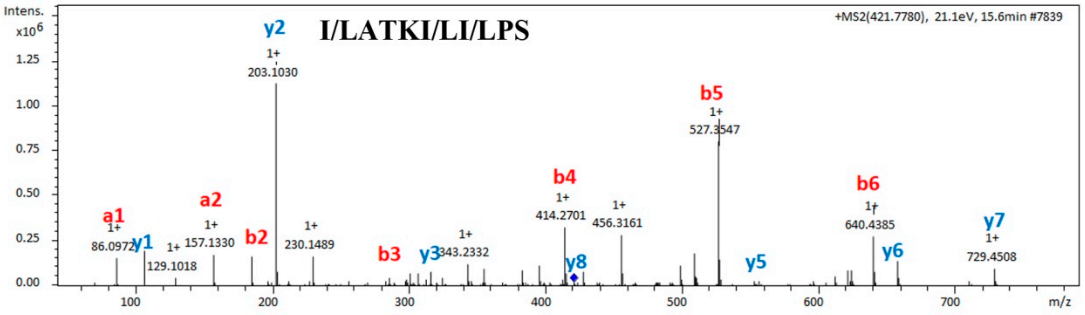

Supplementary Figure 143. Mass spectra and de novo sequencing analysis of

I/LATKI/LI/LPS

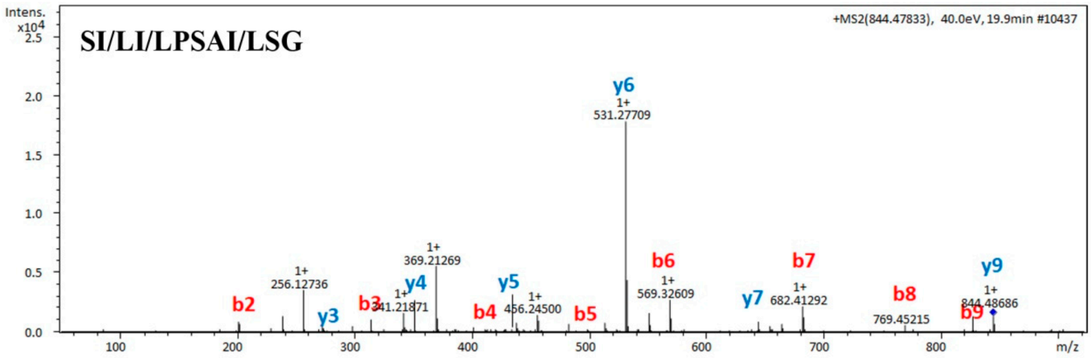

Supplementary Figure 144. Mass spectra and de novo sequencing analysis of

SI/LI/LPSAI/LSG

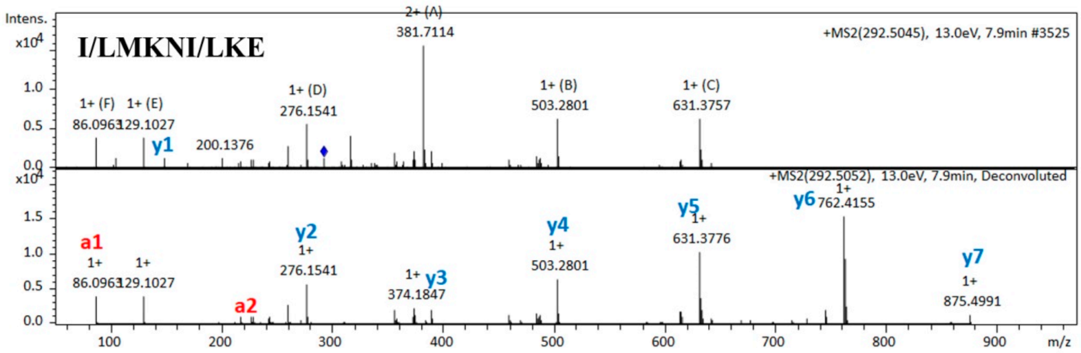

Supplementary Figure 144. Mass spectra and de novo sequencing analysis of

I/LMKNI/LKE

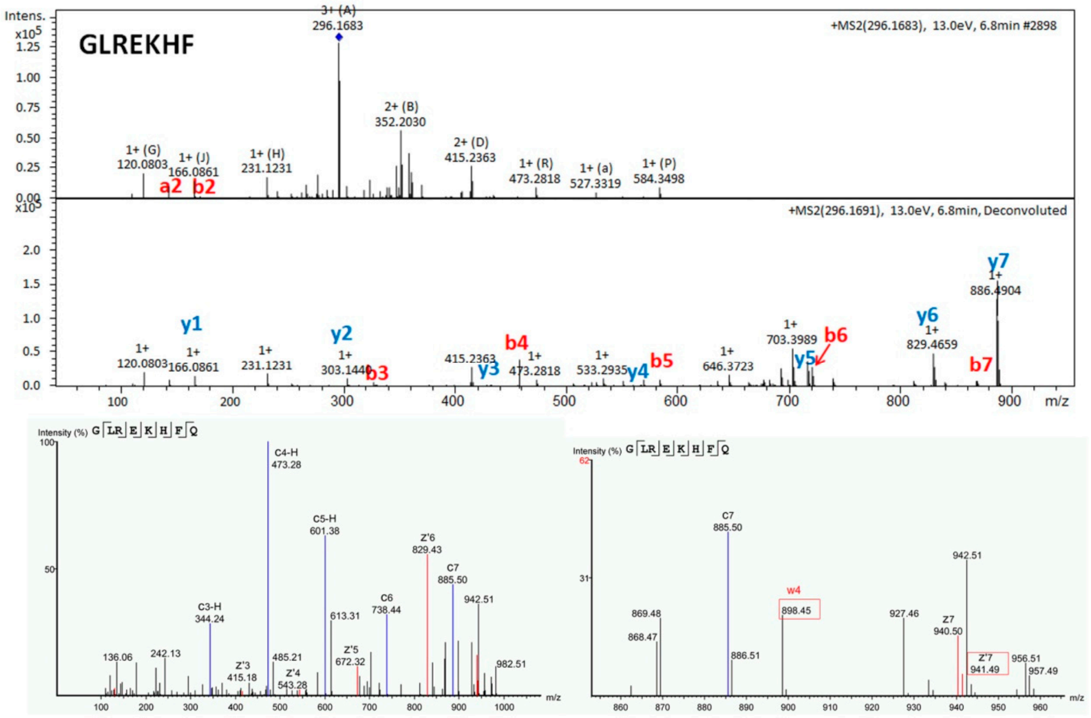

Supplementary Figure 146. Mass spectra and de novo sequencing analysis of GLREKHF

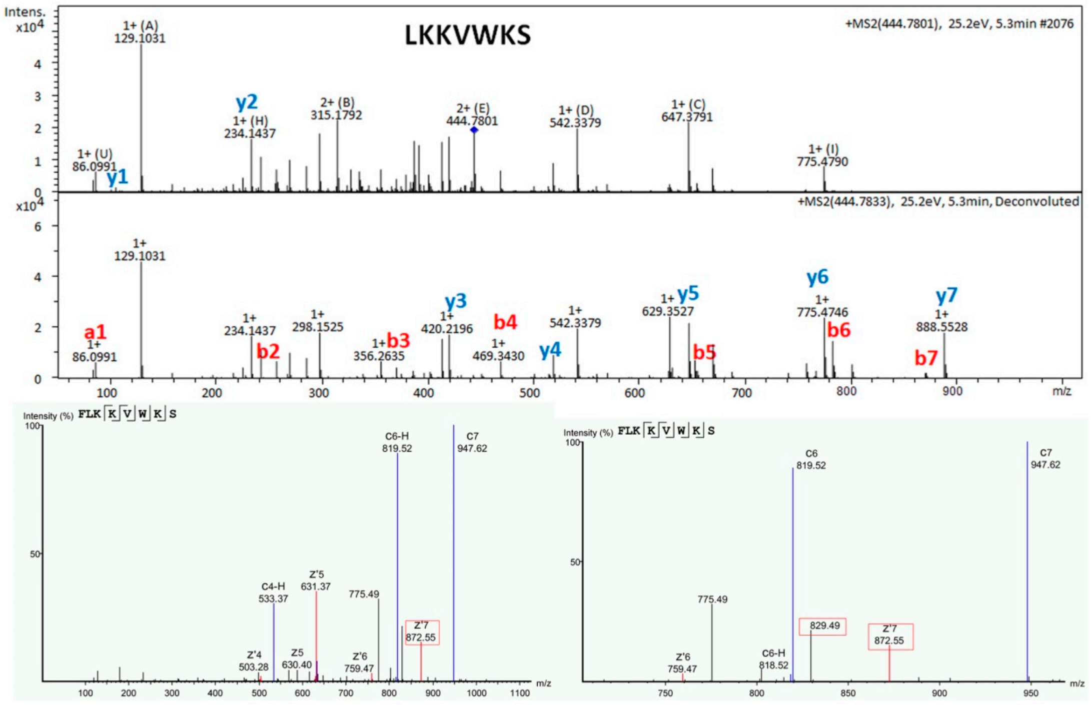

Supplementary Figure 147. Mass spectra and de novo sequencing analysis of LKKVWKS

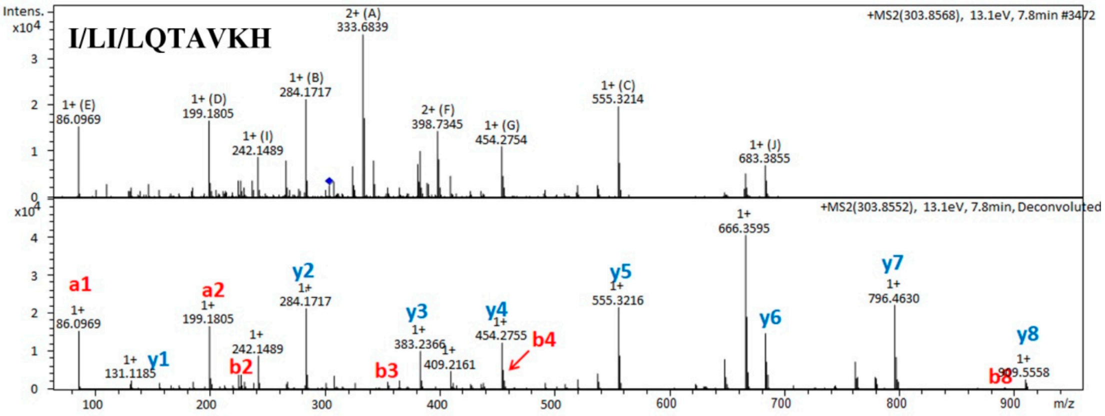

Supplementary Figure 148. Mass spectra and de novo sequencing analysis of

I/LI/LQTAVKH

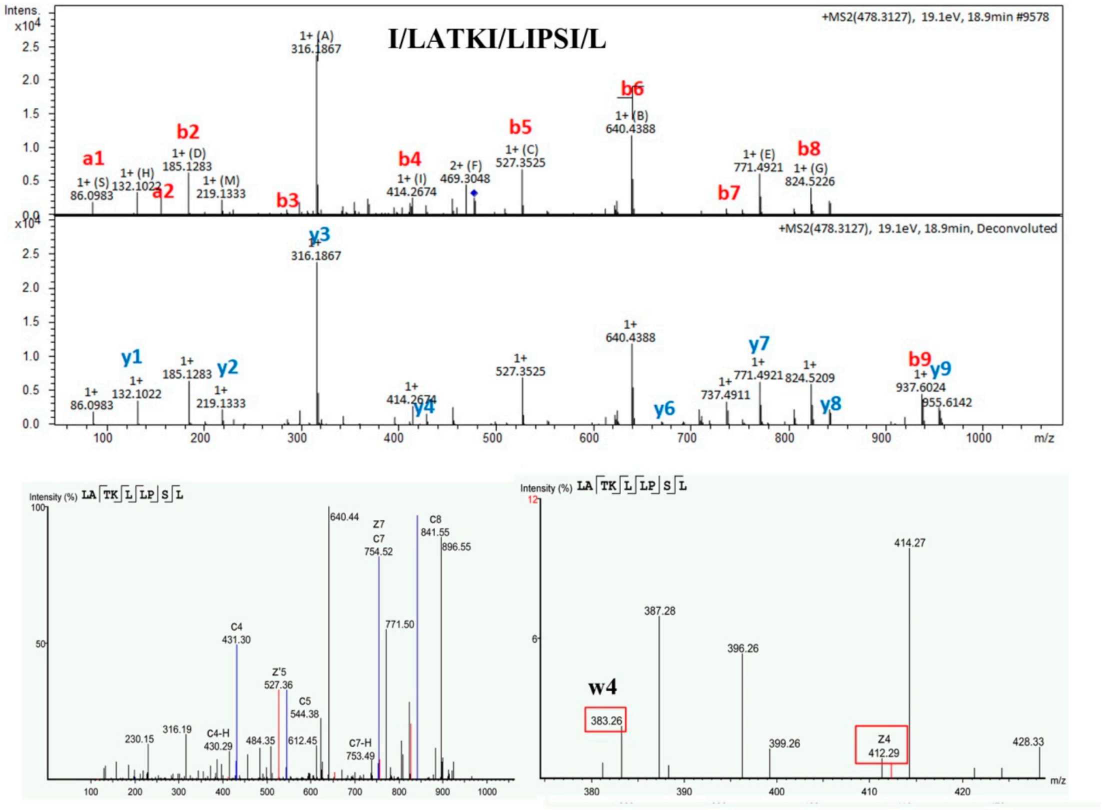

Supplementary Figure 149. Mass spectra and de novo sequencing analysis of

I/LATKI/LIPSI/L

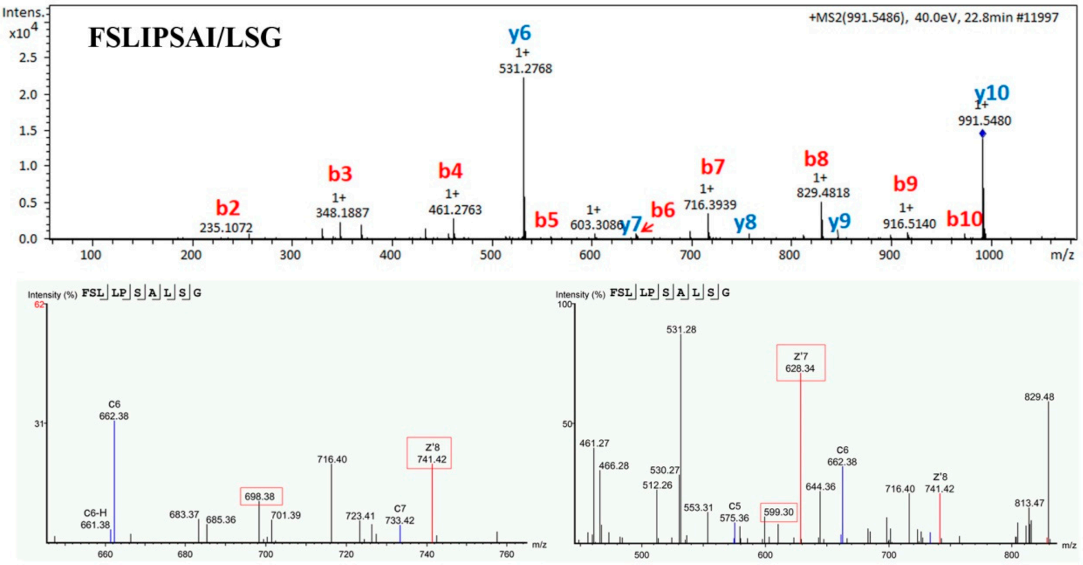

Supplementary Figure 150. Mass spectra and de novo sequencing analysis of FSLIPSAI/LSG

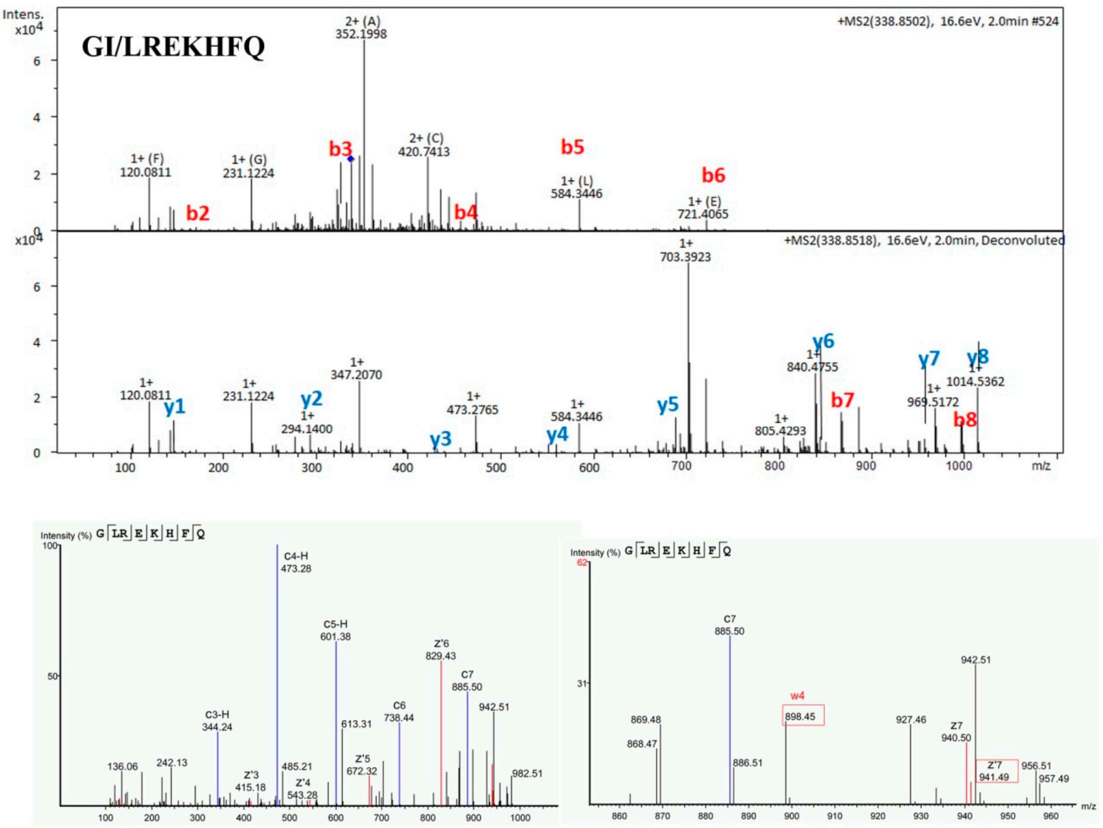

Supplementary Figure 151. Mass spectra and de novo sequencing analysis of

GI/LREKHFQ

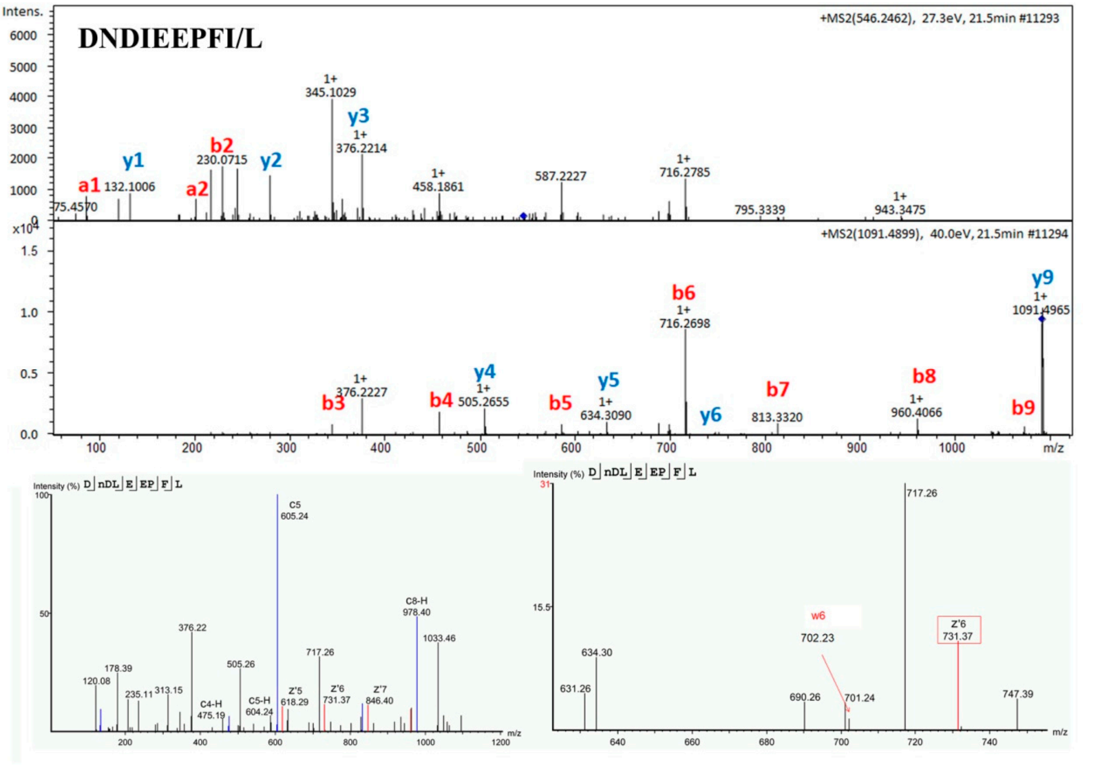

Supplementary Figure 152. Mass spectra and de novo sequencing analysis of

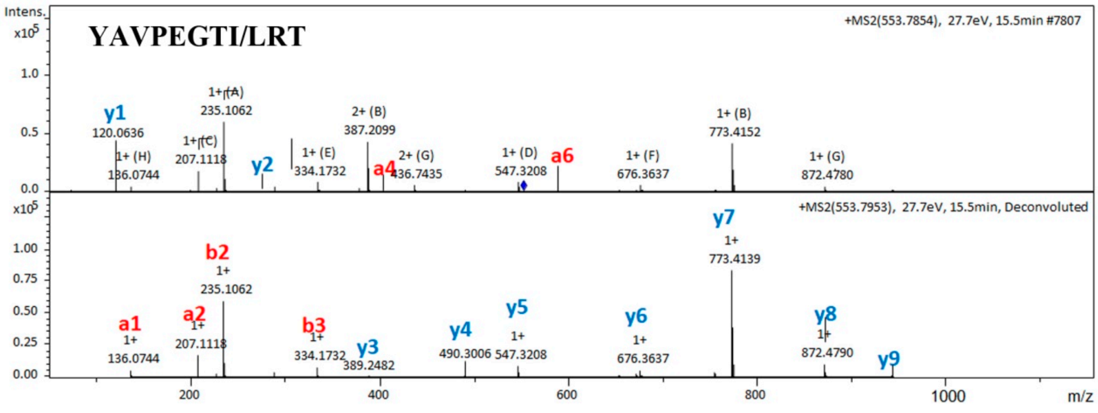

Supplementary Figure 153. Mass spectra and de novo sequencing analysis of

YAVPEGTI/LRT

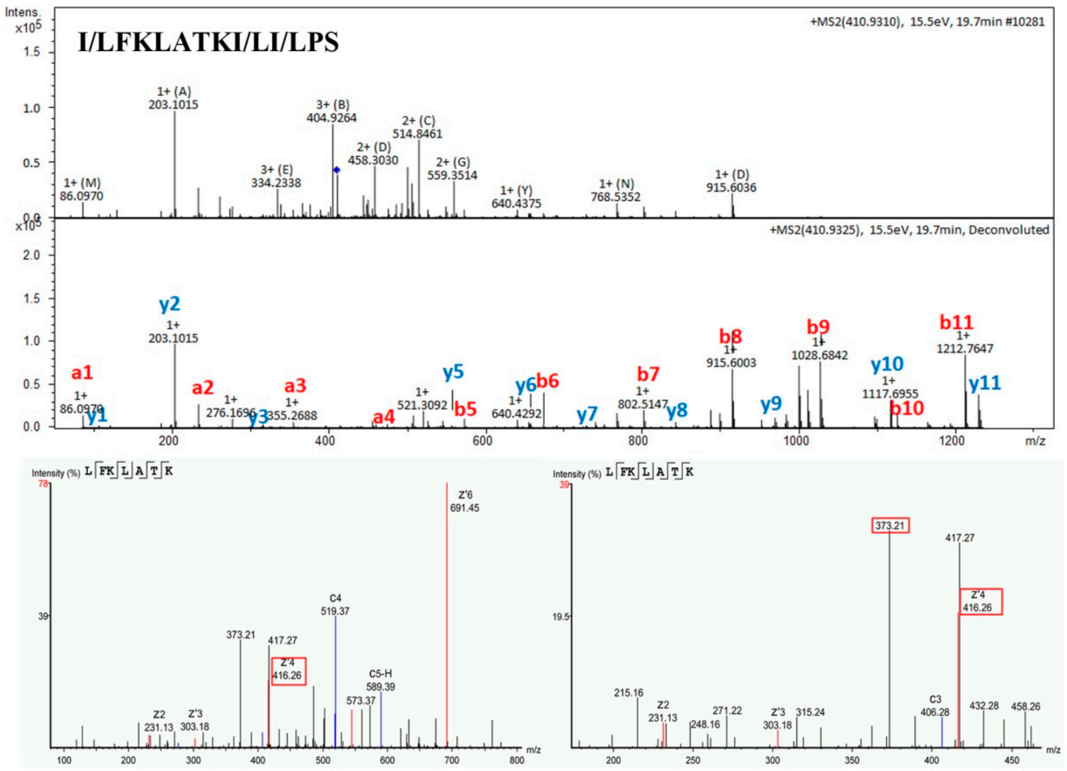

Supplementary Figure 154. Mass spectra and de novo sequencing analysis of I/LFKLATKI/LI/LPS

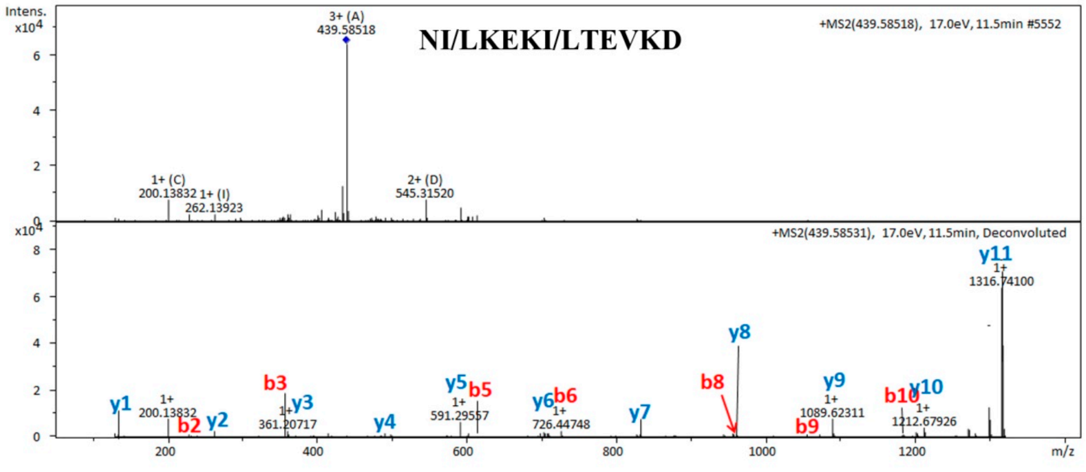

Supplementary Figure 155. Mass spectra and de novo sequencing analysis of NI/LKEKI/LTEVKD

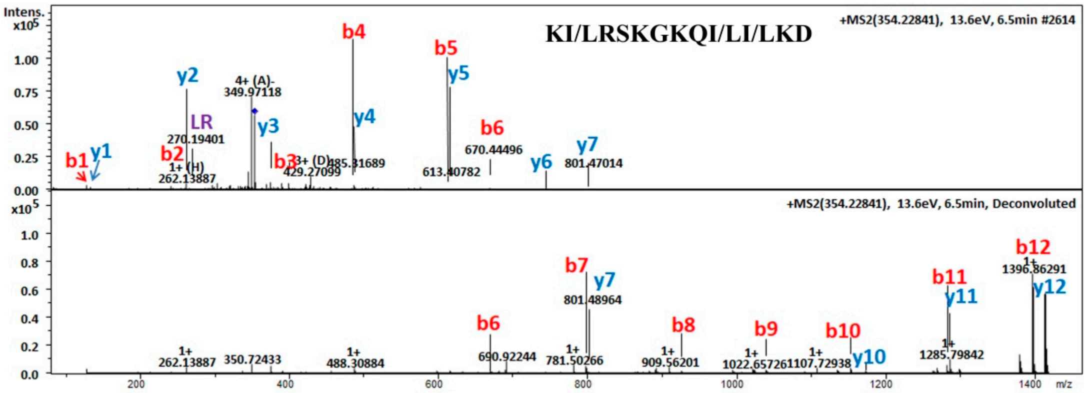

Supplementary Figure 156. Mass spectra and de novo sequencing analysis of

KI/LRSGKKQI/LI/LKD
